# miRNA delivery via nanowires restores functional responses in aged T cells

**DOI:** 10.1016/j.celbio.2026.100514

**Published:** 2026-07-03

**Authors:** Zhonghao Dai, Shaylyn Grier, Zhe Zhong, Ameya Dravid, Joscelyn Mejías, Jean L. Koff, Andrés J. García, Ankur Singh

**Affiliations:** 1 Wallace H. Coulter Department of Biomedical Engineering, Georgia Institute of Technology, Atlanta, GA 30332, USA; 2 Petit Institute for Bioengineering and Biosciences, Georgia Institute of Technology, Atlanta, GA 30332, USA; 3 George W. Woodruff School of Mechanical Engineering, Georgia Institute of Technology, Atlanta, GA 30332, USA; 4 Winship Cancer Center, Emory School of Medicine, Emory University, Atlanta, GA 30322, USA; 5 Lead contact

## Abstract

Aging reshapes CD8+ T cell immunity by depleting naive populations, narrowing repertoire diversity, and promoting dysfunctional states, thereby weakening responses to infection or vaccination. This study presents a polyethyleneimine (PEI)-functionalized silicon nanowire platform for high-viability, non-viral delivery of single or multiplexed microRNAs (miRNAs) into resting aged CD8+ T cells. Nanowires enable transient modulation of transcriptional programs governing T cell activation, metabolism, proliferation, and differentiation. Delivering a single miRNA influences early activation and differentiation stages, while multiplexed miRNA delivery leads to a broader boost in activation responses, effector functions, metabolic health, and cell proliferation. *In vivo*, *ex vivo*-treated aged CD8+ T cells exhibit enhanced early effector priming following antigen exposure. Similarly, in aged human CD8+ T cells, multiplexed miRNA enhances activation and cytotoxic responses and sustains an effector-primed state during early activation and expansion. This method consistently improves function across donors aged from healthy individuals to those with current or past cancer.

## In brief

Aging reshapes CD8+ T cells, reducing their ability to activate, expand, and respond to infection or vaccination. Singh and colleagues use biomaterial-functionalized silicon nanowires to deliver microRNAs into aged T cells with high efficiency and viability. Multiplexed delivery transiently enhances activation, metabolic fitness, proliferation, and cytotoxic function in mouse and human cells, including patient samples, highlighting a non-viral strategy to retune immune responses and strengthen immunity in older adults.

## Graphical abstract

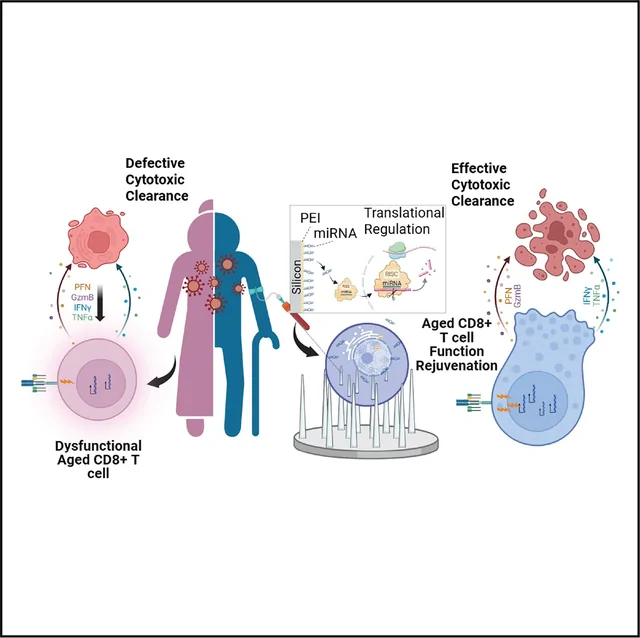

## INTRODUCTION

Aging induces a multifaceted decline in adaptive immunity, particularly affecting CD8+ T cells.^1^ The aging T cell compartment exhibits thymic involution, characterized by a shrinking naive T cell population, expansion of memory cell clones, and an accumulation of senescent and partially differentiated cells, along with a decrease in T cell receptor (TCR) diversity.^2–5^ These changes are accompanied by cell-intrinsic modifications that collectively restrict cell proliferation, cytokine production, and long-term memory development.^6–8^ External aging factors, including disrupted cytokine environment, impaired antigen-presenting cell function, and diminished co-stimulatory signals, further hinder T cell priming and differentiation.^9,10^ Overall, these factors produce a T cell compartment that is less responsive to infections and prone to dysfunctional activation. In humans, this manifests as markedly increased infection susceptibility and poorer vaccine responses in older adults (≥65 years).^11,12^

At the level of transcriptional regulation, the T-box transcription factor T-bet (Tbx21) serves as a key determinant of CD8+ T cell destiny.^13–15^ T-bet integrates signals from TCR, inflammatory cytokines, particularly interleukin (IL)-12 and interferon (IFN)-γ, and metabolic cues to promote effector programs, including IFN-γ production, expression of cytotoxic granules, and rapid clonal expansion. A balanced induction of T-bet supports timely effector responses during acute infection while allowing contraction and memory formation following pathogen clearance.^16–18^ In aged CD8+ T cells, this equilibrium is disrupted: impaired induction of T-bet and downstream effector modules can hinder early pathogen elimination in acute infections.^19,20^ Conversely, long-term dysregulated or excessive effector skewing in other contexts may accelerate terminal differentiation and deplete the reservoir of memory (T_Memory_) and long-lived precursors.^21–23^ Memory T cells, which are characterized by the CD62L^hi^CD44^hi^ phenotype, are essential for strong recall responses due to their proliferative capacity, ability to home to lymph nodes, and potential to generate diverse effector progeny. Consequently, the decline in T_Memory_ representation with age compromises both immediate and secondary immune responses.^24^

The effects of these age-related changes vary depending on the context of the disease. In acute infections, the aged CD8+ T cell population often struggles to generate the rapid, robust effector responses necessary to control rapidly replicating pathogens, resulting in higher pathogen loads and more severe illness.^25,26^ In chronic infections and cancer, continuous exposure to antigens leads to exhaustion, characterized by persistent expression of inhibitory receptors, decreased proliferation, and a gene expression profile that remains epigenetically fixed and dysfunctional.^3,6,27^ Importantly, sustained control in chronic conditions relies on a subset of stem-like, T cell factor 1 (TCF1)+ progenitor cells that maintain the exhausted T cell pool and respond to checkpoint therapy.^28^ However, age-related loss or altered programming of these progenitors diminishes the ability to achieve long-term control or immunotherapy rescue.^29^ Therefore, approaches that can restore or balance transcriptional regulators, such as modulating T-bet and associated pathways, may enhance quick pathogen clearance and maintain progenitor cells crucial for managing chronic infections and fostering anti-tumor responses.

MicroRNAs (miRNAs) are potent and compact regulators of transcriptional networks, presenting an attractive mechanism to modulate T cell fate without inducing permanent genomic alterations.^30–33^ In aged CD8+ T cells, several miRNAs are dysregulated in ways that contribute to impaired activation, effector differentiation, and maintenance of homeostasis. For example, miR-181a declines in naive CD8+ T cells with age in both humans and mice; this reduction raises the threshold for TCR signaling by failing to repress phosphatases, resulting in weaker activation, reduced IL-2 production, impaired expansion of antigen-specific clones, and diminished vaccine responses.^34,35^ Concurrently, miR-146a, which is often upregulated with age, dampens TCR-proximal signaling, reduces expression of antiviral cytokines (IFN-γ, IL-2, tumor necrosis factor α [TNF-α]), and suppresses the production of cytotoxic molecules (e.g., perforin, granzyme B).^36–38^ Another miRNA, miR-29, has been shown to act as a brake on effector differentiation. miR-29 directly targets the master transcription factors T-bet and suppresses IFN-γ transcription, thereby limiting Th1 differentiation.^39,40^ miR-29 also defines age-related differences in naive CD8+ T cell phenotype.^41^ Finally, components of the miR-17–92 cluster (miR-17, miR-19, miR-20a, and miR-106a) are downregulated in aged memory/effector CD8+ populations, a shift that potentially reduces proliferative and metabolic fitness given their roles in promoting survival, glycolysis, and expansion.^38,42^ Therefore, restoring youthful miRNA expression in aged CD8+ T cells is critical for recovering the signaling and transcriptional programs that support effective activation, effector function, and homeostasis. We hypothesize that delivery of key miRNAs can improve aged T cell activation, cytokine production, and proliferative potential, while also reducing exhaustion, ultimately improving immunity to levels closer to those of young CD8+ T cells.

Nonetheless, delivery of small RNAs into primary CD8+ T cells, especially from aged hosts, has proven technically challenging due to reduced membrane fluidity, increased susceptibility to transfection-induced stress, and inherent resistance to uptake, rendering viral and non-viral methods either inefficient or toxic in aged cells.^43–47^ T cell-targeted delivery of nucleic acids is most efficient when the cells are in an activated, dividing state.^48,49^ However, the T cell population in aged individuals often comprises a high percentage of quiescent or exhausted cells, which are less susceptible to nucleic acid uptake. The efficiency of lipid-nanoparticle-based delivery systems depends not only on endosomal acidification, which protonates ionizable lipids and facilitates cargo release, but also on factors such as the ionizable-lipid chemistry of formulations (pK_a_, helper-lipid composition, and cholesterol/polyethylene glycol [PEG] composition), the uptake and intracellular trafficking route of nanoparticles, and its capacity to destabilize endosomal membranes through fusion, phase transitions, or budding/collapse before lysosomal degradation.^50–52^ Studies show that T cells, especially aged ones, have a lower endosomal acidification rate compared with other cell types, which can reduce the effectiveness of these pH-triggered mechanisms.^53–55^

Cationic biomaterial-functionalized nanowires represent a versatile class of biomaterials with broad potential in biomedical applications. By coating nanowire surfaces with positively charged polymers such as polyethyleneimine (PEI), chitosan, or other cationic biopolymers, their surface chemistry can be tailored to enhance interactions with negatively charged biomolecules, including nucleic acids, proteins, and cell membranes.^56–59^ This functionalization promotes strong electrostatic adsorption, enabling nanowires to serve as efficient vehicles for gene delivery, biosensing, and immune modulation.^60–65^ We have adapted a high-aspect-ratio silicon nanowire platform, covalently functionalized with PEI, to surmount these barriers.^66^ The high aspect ratio of nanowires and tunable surface chemistry enable direct and localized delivery of nucleic acids, proteins, or small molecules across refractory membranes, while minimizing cellular stress and toxicity. Unlike conventional viral or chemical transfection methods, nanowires function as passive, cell-interfacing scaffolds that preserve viability and phenotype, making them advantageous for manipulating primary immune cells and other hard-to-transfect populations. Moreover, their cytocompatibility, scalable fabrication, and amenability to multiplexed functionalization position silicon nanowires as a broadly applicable platform for precision modulation of cell states, with implications for regenerative medicine, immunotherapy, and translational bioengineering.

In this study, we employed PEI-functionalized nanowires to transiently enhance functional responses in aged murine CD8+ T cells through the programmable delivery of miRNA. We initially established efficient, high-viability delivery in aged cells and demonstrated that specific single miRNA inputs can modulate the T cell transcriptome at baseline and sustain this modulation through early activation. This biasing influences differentiation toward either memory- or effector-leaning states without disrupting resting subset frequencies. Building upon this finding, we implemented a multiplexed-miRNA cocktail comprising miR-29 antisense oligonucleotide (ASO), miR-130, miR-150, and miR-181a. Delivery of these multiplexed miRNAs to aged T cells correlates with accelerated activation kinetics, a shift toward an effector phenotype, enhanced cytotoxic capacity, and increased proliferation and expansion. Moreover, upon adoptive transfer into aged recipient mice challenged with influenza, the multiplexed miRNA-programmed aged CD8+ T cells exhibited stronger early effector priming *in vivo* compared with saline and inert-miR controls. Importantly, primary aged human CD8+ T cells programmed with the same single- and multiplexed miRNAs targeting transcriptional networks and signaling pathways governing T cell functions demonstrated consistent shifts toward early functional priming at both early and extended post-activation stages, without a concomitant rise in exhaustion phenotype following repeated stimulations. Furthermore, across multiple donors, including lymphoma survivors years after completion of chemoimmunotherapy and patients recently diagnosed with solid tumor cancers, multiplex miRNA delivery restored activation and effector molecule expression, maintaining consistent improvements across various clinical backgrounds. These results delineate a transient, non-viral, multiplexed approach for *ex vivo* reconditioning of aged CD8+ T cells, suggesting a translational pathway to enhance vaccine responses and improve adoptive cell therapies in older individuals while preserving the flexibility to balance effector functions with progenitor preservation.

## RESULTS

### Functionalized nanowires deliver miRNA to aged murine CD8+ T cells with high viability and delivery efficiency

Efficient, non-viral delivery of nucleic acids to primary T cells, especially those from aged donors, remains technically challenging due to reduced membrane fluidity and heightened susceptibility to transfection-induced stress. We first attempted to deliver miRNA to aged T cells using state-of-the-art nucleofection. In the resting state, ~30% miR-29-fluorescein isothiocyanate (FITC) was delivered to aged T cells (18- to 24-month old C57BL/6 mice, Figure S1A). Even with pre-activation of the aged T cells with anti-CD3/CD28 Dynabeads, only ~50% of the miRNA was delivered, and the nucleofection procedure killed nearly 50% of resting aged T cells. To confirm the aged phenotype of the murine CD8+ T cells, we performed senescence-associated β-galactosidase (SA-β-gal) staining in young and aged CD8+ T cells at baseline and after activation. Aged CD8+ T cells showed a modest increase in SA-β-gal signal at day 0, and this difference became more pronounced after 48 h activation (Figure S1B), supporting the use of this model for subsequent delivery studies. In contrast, pre-activation of young T cells (from 3- to 6-month-old mice) followed by nucleofection delivered ~75% miR-29-FITC to young T cells. The delivery efficiency to resting young T cells was only 20%, levels consistent with our recent findings.^66^ Therefore, the major roadblock to achieving efficient miRNA delivery into primary aged T cells is that pre-activation does not sufficiently overcome the delivery barrier in this population.

Consequently, we adapted our previously reported silicon nanowires, covalently functionalized with PEI, to provide a cationic surface that complexes and drives intracellular delivery of miRNAs to aged CD8+ T cells without pre-activation.^66^ Scanning electron microscopy (SEM) revealed a uniform array of nanowires, separated by 3 μm, with basal diameters of approximately 150 nm, tapering to 20 nm at the tip, and a height of 3.5 μm (Figure 1A). We previously demonstrated that this geometry provides sufficient rigidity for cell interfacing while ensuring high viability and T cell health,^66^ as it prevents puncturing through the entire cell diameter. A spacing of 0.11 nanowires per μm^2^ (Figure 1A) enables broader tips and bases, as well as biofunctionalization, such as loading multiple miRNAs. We note that covalent conjugation of PEI is essential because silicon nanowires coated with physically adsorbed PEI reduced T cell viability to 20%–30%,^66^ likely due to the cytotoxic release of free PEI. However, covalent conjugation of PEI (10% weight/volume [w/v]) via an N-hydroxysuccinimide (NHS)-silane linker resulted in >90% delivery of miRNA into naive young CD8+ T cells while maintaining over 90% viability.^66^ Successful covalent conjugation of PEI onto the silicon nanowire surface has been established in our prior study^66^ through multiple complementary characterization approaches, including validation of the covalent functionalization, comparison of nanowires that are covalently conjugated versus surface coated with PEI for delivery efficiency and cell viability, scanning transmission electron microscopy and energy-dispersive X-ray spectroscopy, zetapotential measurements following PEI functionalization and miRNA loading, and microscopy-based assessment of nanowire-cell interactions.

The nanowires act as nanoscale conduits, physically penetrating the plasma membrane under the effect of 50 × *g* centrifugal force, as optimized by us against larger centrifugation forces,^66^ and directly releasing miRNA cargo into the cytosol. We initially examined how the nanowire interfaces physically with aged and young T cells and whether it could access the cell interior. Confocal imaging of 6-carboxyfluorescein (6-FAM) labeled miRNA attached to PEI-coated nanowires revealed similar numbers of nanowire-cell contacts in both young and aged CD8+ T cells (~5 ± 1 nanowires per cell), suggesting comparable levels of penetration with nanowires (Figures 1B and S2A). The 3D confocal reconstructions and orthogonal sections (Figure 1C) further revealed that PEI-coated nanowires can enter the cell membrane and reach the cytosol of both aged and young T cells in a similar manner.

Flow cytometry confirmed effective delivery of 6-FAM-miR-29 in both age groups using PEI nanowires: >90% of CD8+ T cells were 6-FAM+ without any significant reduction in viability compared with nanowire-free controls (Figures 1D and S2B). In these studies, we did not focus on naive T cells, as their frequency in aged mice (20%–30% of total splenic T cells) is markedly reduced compared with younger mice (60%–75% of total splenic T cells). The median fluorescence intensity (MFI) increased ~5-fold with nanowire delivery, and there was no age-related decrease in cellular loading (Figure S2C). These findings demonstrate that nanowires facilitate the delivery of miRNA to aged CD8+ T cells at levels comparable to those in young cells, which is an important factor for *ex vivo* programming of aged T cells.

To determine whether nanowire-mediated uptake relies on traditional endocytic pathways, we used chemical inhibitors dynasore^67^ and chlorpromazine^68^ to block endocytosis and compared the delivery of 6-FAM-miR-29 via nanowires, nucleofection, Lipofectamine, and 2D culture. Inhibition of endocytosis did not significantly decrease nanowire-driven uptake and had little impact on nucleofection, while Lipofectamine-mediated delivery was notably reduced under the same conditions (Figure 1E). These findings indicate that nanowire-based miRNA delivery is largely independent of classical endocytic mechanisms.

Extending to multiplexed miRNA delivery, we co-loaded nanowires with 6-FAM-miR-29 and Cy5-miR-130 and compared their co-delivery to nucleofection-based co-delivery. The nanowires successfully delivered >90% of both miRNAs (6-FAM-miR-29-ASO and Cy5-miR-130-mimic) to aged T cells, while preserving high cell viability (Figure 1F). In contrast, nucleofection showed significantly lower delivery rates and reduced cell viability. Quantitative analysis, normalized to the average single-cell signal relative to nanowires, revealed that nucleofection reached only 40% of the delivery efficiency for each miRNA (Figure 1G). These findings demonstrate that cytocompatible, PEI-coated silicon nanowires overcome the delivery challenges associated with aged T cells, enabling effective single- or multiplexed miRNA uptake without compromising T cell health. This method provides a reliable platform for *ex vivo* transcriptional programming of aged CD8+ T cells, highlighting the broader potential of nanowires as biomaterial scaffolds for manipulating challenging-to-transfect immune cells.

### Single miRNA delivery modulates transcription, activation, and responses in aged CD8+ T cells

We next investigated whether a single miRNA input could modify the baseline transcriptional state and early fate decisions of aged T cells similarly to those of naive T cells, as previously demonstrated.^66^ Here, we selected five candidate miRNAs—miR-29, its antisense inhibitor (miR-29-ASO), miR-130, miR-150, and miR-181a—based on prior reports linking them to T cell activation dynamics and their dysregulation during immune aging. Each miRNA has been implicated in distinct aspects of T cell biology: miR-29 directly targets Tbx21 (encoding T-bet), miR-130 regulates IL-2 responsiveness, miR-150 influences differentiation and survival pathways, and miR-181a modulates TCR sensitivity thresholds.^32,35,38,41,69^ Thus, this panel of individual functional miRNAs enabled us to assess whether tuning distinct regulatory axes could reset the transcriptional tone of aged CD8+ T cells.

We first examined subset composition immediately after nanowire incubation (48 h on nanowires), using no-miR and inert-miR controls to determine sequence-specific effects (Figure S3A). In our prior study^66^ with young mouse and human CD8+T cells, the inert-miR nanowire condition did not induce an active-miRNA-associated change in proliferation, activation, differentiation, and effector readouts, supporting its use as a negative control in current studies. Across aged CD8+ T cell groups, nanowire exposure and miRNA loading did not alter subset frequencies, which remained ~35% T_Naive_ (CD44−CD62L+), ~45% T_Memory_ (CD44+CD62L+), and ~20% T_Effector_ (CD44+CD62L−). Young CD8+ T cells measured at the same post-transfection time point displayed a more naive-skewed profile (~72% T_Naive_, ~23% T_Memory_, and ~3.5% T_Effector_), reflecting their inherently less differentiated baseline relative to aged T cells (Figures 2A and 2B). We then quantified expression of T-bet, a transcription factor tightly linked to effector programming.^41^ Consistent with prior reports, basal T-bet levels were higher in young CD8+ T cells relative to aged counterparts across subsets (Figure 2C), reflecting relatively preserved effector programming capacity in young cells versus impaired transcriptional induction in aged counterparts. In aged T cells, among the five tested functional miRNAs, only miR-29 mimic and miR-29-ASO produced marked, reciprocal effects on T-bet expression in aged CD8+ T cells: the mimic reduced T-bet by ~30%, while the ASO increased it by ~25% relative to inert controls (Figure 2C). These differences were also observed across subsets (Figure S3B), suggesting a broad modulation of effector-associated transcriptional tone. In contrast, miR-130, miR-150, and miR-181a, although previously implicated in T cell activation and effector responses, did not significantly alter T-bet expression or shift baseline transcriptional tone. This specificity underscores that only miRNAs directly linked to the T-bet regulatory axis (here, miR-29^41^) are capable of rewiring the transcriptional preset in aged CD8+ T cells. Accordingly, we focused subsequent analyses on miR-29 mimic and miR-29-ASO as functionally informative regulators within this axis.

We asked whether these baseline adjustments translate into altered early differentiation trajectories upon activation. Following 48 h nanowire delivery and a subsequent 48 h activation period, inert-miR and no-miR groups showed similar trajectories. Notably, nanowire-mediated delivery of miR-29-mimic increased memory fractions by 10% and reduced effector subsets by 10%, whereas miR-29-ASO moderately favored differentiation into effector phenotype by 5% (Figure 2D). Importantly, miRNA-mediated modulation of T-bet persisted after activation. Relative to blank nanowire-exposed cells, miR-29 mimic reduced T-bet expression in all subsets by ~25%, whereas miR-29-ASO increased T-bet expression by ~25% (Figure S3C). These findings indicate that pre-activation miRNA programming remains detectable through the early activation window rather than being erased by TCR stimulation. Together, these data show that nanowire-mediated delivery of a single miRNA can alter transcriptional tone and bias the early differentiation trajectory of aged CD8+ T cells.

We examined whether these changes extended to activation kinetics and downstream effector programs after TCR stimulation. T cell activation involves coordinated regulation of surface markers, co-stimulatory molecules, and trafficking receptors that together determine the amplitude and timing of subsequent effector responses.^70,71^ We therefore asked whether single miRNA delivery could recalibrate this activation cascade in aged CD8+ T cells. Following 48 h of nanowire incubation and subsequent 48 h of activation with anti-CD28/anti-CD3 beads, aged CD8+ T cells exhibited coordinated shifts in activation marker profiles that mirrored the directionality of T-bet modulation (Figures 2E and S4A). Nanowire-mediated delivery of miR-29 mimics reduced the frequency and per-cell intensity of CD69, an early TCR-proximal activation marker, and CD28, a crucial co-stimulatory receptor that facilitates IL-2 production and clonal expansion, by 20% and 10%, respectively. At the same time, miR-29 mimic induced higher expression of CD62L, a lymphoid-homing receptor whose retention reflects a more quiescent or memory-leaning state.^72^ In contrast, miR-29-ASO promoted the reciprocal phenotype with expanded CD69+ and CD28+ fractions by 10%, higher MFI of both molecules by >20%, and a diminished CD62L+ compartment, consistent with a more effector-primed activation set point. These reciprocal shifts indicate that single miRNA delivery does not merely alter a single isolated marker but modulates the broader activation-associated profile of aged CD8+ T cells.

Effector function mirrored these activation phenotypes at both intracellular and secreted levels. Intracellular staining revealed that miR-29-mimic reduced the frequency of the cytotoxic molecule perforin by 50% and pro-inflammatory cytokine TNF-α by >80%, as well as reduced normalized per-cell expression of granzyme B, perforin, IFN-γ, and TNF-α. In contrast, miR-29-ASO increased the frequency of cytotoxic molecule perforin by 50% and pro-inflammatory cytokine TNF-α by 17%, as well as enhanced expression of granzyme B, perforin, IFN-γ, and TNF-α (Figures 2F and 2G). These findings suggest that modulation of the miR-29 axis establishes distinct thresholds for functional output in aged CD8+ T cells: the mimic biases cells toward a more restrained state, while the ASO favors their transition to a stronger early effector state deployment. Supernatant cytokine measurements provided orthogonal confirmation of this pattern, with secreted IFN-γ reduced by ~60% and TNF-α reduced by ~50% in the miR-29-mimic group, whereas IFN-γ increased by ~40% and TNF-α by ~20% in the miR-29-ASO group relative to controls (Figure S4B). This indicates that intracellular modulation of transcriptional and activation-associated state translates into measurable changes in extracellular effector release.

We further examined whether these effects were restricted to a specific differentiation compartment. Subset-resolved analysis of activation and effector marker expression showed that miRNA-mediated regulation was not limited to a single differentiation stage but was observed across naive, memory, and effector groups. miR-29 mimic and miR-29-ASO oppositely affected activation-related markers CD44, CD69, CD28, and CD62L in each CD8+ T cell subset (Figures S5A–S5D). The trends aligned with those observed in the overall population: miR-29-ASO increased activation and co-stimulatory marker levels while decreasing CD62L, and miR-29 mimic had the opposite effect. Likewise, effector molecule expression was also modulated independently of the subset. The frequency and per-cell expression of granzyme B, perforin, IFN-γ, and TNF-α followed the same order (miR-29 mimic < inert < miR-29-ASO) in memory and effector T cells (Figures S6A–S6D). These results confirm that miRNA programming influences the transcriptional and functional states of CD8+ T cells across multiple differentiation stages. Overall, these data show that nanowire-delivered single miRNA inputs can modulate both activation behavior and effector capacity in aged CD8+ T cells. By linking early activation markers (CD69, CD28, and CD62L) to transcriptional regulation (T-bet) and functional outputs (cytotoxic proteins and cytokines), we demonstrate that a single miRNA axis provides a tunable means to modulate aged T cell responses.

### Multiplexed miRNA delivery shifts aged CD8+ T cells to a strong effector-dominant state

We next investigated whether combining miRNAs targeting complementary bottlenecks in aged CD8+ T cell functionality produces an enhanced, potentially combinatorial effect relative to individual inputs, resulting in broader and more coordinated modulation. Our approach is based on the rheostat framework established earlier: antagonizing miR-29 to alleviate repression of effector programs, and augmenting miR-130 to expedite effector acquisition. Additionally, we incorporated two miRNAs targeting distinct bottlenecks in aged T cell function: miR-150, which promotes proliferation and terminal effector differentiation by restraining memory-associated pathways linked to c-Myb regulation, and miR-181a, a critical regulator of TCR sensitivity that declines with age, thereby raising activation thresholds; its restoration decreases the signaling threshold and rescues cytokine output. We hypothesized that a four-component multiplexed cocktail—miR-29-ASO, miR-130, miR-150, and miR-181a (hereafter All-miR)—would produce an enhanced, combinatorial effect in biasing aged CD8+ T cells toward an effector-associated state relative to either single miRNA treatments or the paired miR-29-ASO/miR-130 condition (ASO-mimic), by concurrently augmenting transcriptional activity, priming co-stimulatory pathways, and increasing signaling sensitivity.

We first examined whether nanowire-mediated multiplexed miRNA delivery accelerates and amplifies reprogramming in aged CD8+ T cells toward the effector state. Post nanowire exposure, CD8+ T cells shifted to more effector type than memory type (Figures 3A and 3B), with both a combination of miR-29-ASO + miR-130 (called ASO-mimic), as well as All-miR (miR-29-ASO + miR-130 + miR-150 + miR-181a). Consistent with these phenotypic changes, analysis of T-bet expression revealed a graded increase across the multiplexed conditions, following the hierarchy inert-miR < ASO-mimic < All-miR (Figure 3C). In pan-CD8+ T cells, the All-miR condition elevated normalized T-bet expression to approximately 2-fold above inert control and above the population-average level measured in young CD8+ T cells. This observation should be interpreted in the context of subset composition. Young murine CD8+ T cells have a larger naive compartment, and naive cells intrinsically express lower levels of T-bet than activated memory or effector cells. Thus, the apparent elevation above the young average in All-miR-treated aged cells is best understood as reflecting an engineered shift toward a more effector-biased baseline, rather than implying that these cells are intrinsically superior to young counterparts. Importantly, this elevated transcriptional tone was established entirely within the nanowire culture window, without altering the proportions of resting subsets.

We subsequently inquired whether this modified pre-activation state would endure during initial TCR stimulation and manifest as a more pronounced effector-biased differentiation pathway. Upon 48 h of TCR stimulation with anti-CD28/anti-CD3 beads, multiplex delivery further reinforced effector-biased differentiation trajectories of aged CD8+ T cells. Analysis on T cell differentiation trajectory revealed that both miRNA conditions reduced memory and expanded effector populations, with the All-miR condition producing the largest effector-skewing shift (T_Memory_: ~22%, T_Effector_: ~75%) compared with ASO-mimic (T_Memory_: ~30%, T_Effector_: ~66%) and inert control (T_Memory_: ~38%, T_Effector_: ~57%) (Figure 3E). This pattern suggests that multiplex miR programming creates an activation environment that rapidly drives aged CD8+ T cells away from a memory phenotype and consolidates them within effector-biased states. This interpretation was further supported by T-bet measurements after activation. Across the total CD8+ pool, as well as within the T_Memory_ and T_Effector_ compartments, T-bet expression remained elevated in a graded manner following the same ordering observed at baseline: inert-miR < ASO-mimic < All-miR (Figure 3D). In the All-miR condition, T-bet was increased by approximately 60% over inert control, narrowing or, in selected readouts, surpassing the average values measured in young CD8+ T cells. Again, however, this should not be interpreted as evidence of a fully restored or permanently youthful transcriptional state. Rather, these data indicate that multiplexed miRNA delivery sustained a stronger effector-associated transcriptional bias through the early activation period.

We quantified early activation dynamics to ascertain whether multiplexed miRNA delivery influences not only differentiation trajectories but also the immediate responsiveness of aged CD8+ T cells to anti-CD3/CD28 stimulation. Compared with the inert control, the ASO-mimic condition increased the proportion of CD69+ cells by ~12% and CD28 expression by ~12%, whereas the CD62L− compartment expanded by ~10%, indicating a shift away from a lymphoid-homing, quiescent state. The multiplex All-miR condition further accentuated these changes, enhancing the populations of CD69+ and CD28+ cells by 12% and 20%, respectively, and reducing CD62L expression by ~20% (Figure 3F). This stepwise pattern indicates that the integration of miR-150 and miR-181a into the ASO-mimic backbone did not simply duplicate the effect observed in the paired condition but rather expanded it to encompass multiple activation-associated features. Expression levels reflected frequency shifts; when compared with inert control, All-miR delivery increased the MFI of CD44, CD69, and CD28 by approximately 40%–50%, whereas CD62L MFI decreased, exhibiting the most significant change (Figure 3G). Notably, aged CD8+ T cells treated with All-miR retained a high frequency of CD28+ cells and elevated CD28 expression per cell, features typically associated with more responsive T cells.^73,74^ Although the absolute magnitude of CD28 expression remained lower than that of young cells, it was markedly higher than in the inertmiR control group. The concordance between changes in responder frequency and changes in per-cell marker intensity indicates that multiplexed miRNA delivery elevated the overall activation set point of aged CD8+ T cells in a coordinated manner. Rather than acting on a singular axis, this approach appears to concurrently modulate multiple checkpoints within the activation cascade, thereby establishing a more robust and synchronized entry into the effector program.

### Effector responses increased in tandem with the heightened activation profiles of aged CD8+ T cells

Intracellular staining demonstrated a marked expansion of the cytotoxic compartment: a greater proportion of aged CD8+ T cells produced granzyme B, perforin, IFN-γ, and TNF-α following ASO-mimic programming (granzyme B+: ~95%, perforin+: ~85%, IFN-γ+: ~25%, and TNF-α+: ~50%), with the All-miR condition also producing pronounced increases (granzyme B+: ~95%, perforin+: 85%, IFN-γ+: 30%, and TNF-α+: 60%) compared with the inert control (granzyme B+: 90%, perforin+: 50%, IFN-γ+: 15%, and TNF-α+: 15%) (Figure 3H). Notably, the proportion of cells producing IFN-γ and TNF-α nearly doubled relative to the inert control, indicating that multiplex delivery markedly enhances pro-inflammatory effector capacity. This frequency enhancement was paralleled by per-cell output, as normalized MFIs for each effector increased with ASO-mimic treatment and further with All-miR, ranging from a 2-fold increase for TNF-α, perforin, and granzyme B, to a 3-fold increase for IFN-γ (Figure 3I). This dual improvement underscores two aspects of enhancement: multiplex delivery not only recruits a larger number of CD8+ T cells into the effector pool but also amplifies the amounts of cytotoxic molecules produced per responding cell. When compared against young CD8+ T cells, those treated with the All-miR protocol reached comparable levels of effector molecule production, effectively narrowing the functional gap associated with aging across multiple categories, such as granzyme B and TNF-α. Interestingly, however, young CD8+ T cells in this system demonstrated the highest absolute levels of IFN-γ and TNF-α. This observation contrasts with some reports suggesting that aged CD8+ T cells are hyper-inflammatory; however, this discrepancy can be attributed to key differences in subset composition and signal quality. Young CD8+ T cells possess a larger naive pool that expands vigorously upon stimulation; coupled with intact TCR and cytokine signaling pathways, they are able to generate higher total cytokine output. By contrast, aged CD8+ T cells are skewed toward memory-like states that, while partially pre-activated, are constrained by reduced TCR sensitivity and impaired signal transduction, resulting in limited capacity for cytokine induction.

Subset-resolved and media secretion analysis further confirmed that the impact of multiplexed miRNA delivery extended across all differentiation stages of aged CD8+ T cells. Both ASO-mimic and All-miR conditions increased the expression of CD44, CD69, and CD28 and decreased CD62L intensity relative to inert controls across naive, memory, and effector subsets (Figures S7A–S7D). The hierarchical pattern of activation strength (inert-miR < ASO-mimic < All-miR) was maintained in nearly all subsets, indicating that the multiplex cocktail induces a broad elevation of activation tone rather than a subset-specific shift. The effects on effector molecule expression demonstrated a similarly pervasive trend (Figures S8A–S8D). Expression levels of granzyme B, perforin, IFN-γ, and TNF-α increased stepwise from inert to ASO-mimic to All-miR, with the latter approaching or surpassing the levels observed in young CD8+ T cells within both the memory and effector compartments. Measurements of secreted cytokines provided orthogonal confirmation of this intracellular phenotype: culture supernatants from All-miR-treated aged CD8+ T cells contained higher levels of IFN-γ and TNF-α than inert-miR controls, with ASO-mimic again resulting in an intermediate increase (Figures S8E and S8F).

To more directly determine whether the full four-component cocktail was required or whether similar benefits could be obtained with fewer miRNAs, we next deconvoluted the multiplexed program using reduced-complexity combinations comprising either pairwise or three-component subsets of the All-miR cocktail. Specifically, we compared inert-miR controls with the two-miRNA combinations miR-29-ASO + miR-130 and miR-150 + miR-181a, as well as the three miRNA combinations miR-29-ASO + miR-130 + miR-150 and miR-29-ASO + miR-130 + miR-181a, alongside the full All-miR condition (Figures S9–S11). These experiments were important because they addressed a central interpretive question raised by the multiplexed phenotype: whether the apparent benefit of the cocktail was driven by a single dominant pair or instead reflected broader contributions from all four components.

Several significant findings have emerged from this deconvolution analysis. First, nearly all tested reduced combinations demonstrated improvements in activation-associated endpoints compared with inert-miR controls, suggesting that the activity of the cocktail was not attributable to a single binary interaction. In the analysis of T cell differentiation, the proportion of T_Effector_ cells increased from ~20% in the inert-miR group to ~30% with either the miR-29-ASO + miR-130 or miR-150 + miR-181a pairs, ~35% with the three-component combinations, and reaching ~40% with the full All-miR cocktail (Figure S9). Contributions from all four components were also evident across various activation-associated markers. For example, the proportion of CD69+ cells rose from ~60% in the inert-miR group to 70% with miR-29-ASO + miR-130, 80% with miR-150 + miR-181a and three-component combinations, and ~87% with the All-miR combinations (Figure S10A). The intensity of CD69 expression exhibited a similar trend (Figure S10B), with normalized MFI increased by roughly 45% in the reduced-combination groups and nearly 50% in the All-miR condition. CD28 expression followed a comparable pattern; although the fraction of CD28+ cells remained constant across miRNA combinations (Figure S10C), CD28 MFI was most substantially elevated in the All-miR condition by around 55% relative to the inert-miR control, surpassing the 15%–30% range of increase observed in reduced miRNA combinations (Figure S10D). This indicates a robust reinforcement of co-stimulatory readiness in the full cocktail condition. Concurrently, CD62L MFI decreased progressively—from ~25% in pairwise groups to ~40% in three-component groups, and ~50% in the All-miR condition—relative to control, consistent with a more effector-biased activation state. Collectively, these data suggest that multiple reduced subsets of the miRNA cocktail can measurably shift aged CD8+ T cells toward an effector-associated phenotype, as reflected by enhanced effector differentiation and activation markers. However, none of these subsets replicated the full magnitude and consistency of the All-miR phenotype across differentiation and activation metrics.

Furthermore, the comparative analysis of effector function readouts proved particularly informative in identifying which responses were most sensitive to the complexity of the cocktail. Notably, perforin secretion demonstrated one of the most distinct graded responses (Figures S11A and S11B). The proportion of perforin-secreting cells increased from approximately 40% in the inert-miR group to about 55%–65% in the pairwise and three-component groups, respectively, and exceeded 70% with the full All-miR cocktail. Perforin expression exhibited a similar pattern; reduced combinations showed approximately 30%–60% increases in MFI, with the All-miR group reaching nearly 80%. Secretion of effector cytokines IFN-γ and TNF-α displayed similar progressive responses (Figures S11C and S11D). Aged T cells treated with inert-miR showed low cytokine secretion, with 17% for IFN-γ and 30% for TNF-α, whereas treatment with reduced miRNA cocktails resulted in significant upregulation—roughly 30%–40% for IFN-γ and 40%–50% for TNF-α. Nonetheless, none of these reduced combinations matched the levels achieved with the full All-miR treatment, which reached 45% and 55% for IFN-γ and TNF-α, respectively. Cytokine expression (MFI) further underscored the superior efficacy of the full cocktail, achieving the highest cytokine levels across all groups (approximately 70% increase for IFN-γ, approximately 50% for TNF-α relative to the inert-miR group). Overall, these findings underscore the importance of multiplexed miRNA delivery, as the comprehensive four-miRNA mixture produced the most integrated enhancement across activation, differentiation, and effector-related parameters.

The collective observations—including higher responder frequency, increased effector intensity, broader extracellular cytokine release, and the superior performance of the full cocktail in the reduced-combination analysis—suggest that multiplexed miRNA delivery induces a stronger and more cohesive early effector program in aged CD8+ T cells compared with the tested reduced subsets. Although we do not claim complete equivalence with young CD8+ T cells across all measures, the All-miR condition consistently narrows the functional gap and, in several instances, elevates aged cells to levels comparable to those of young cells. These results establish that expanding beyond the miRNA mimic pair to include a multiplexed strategy yields a qualitatively superior form of reprogramming, accelerating, and fortifying the transition of aged CD8+ T cells into an effector-dominant state.

### Multiplexed miRNA delivery boosts translation-linked metabolism and proliferation in aged CD8+ T cells

To assess whether the activation- and effector-associated gains induced by multiplexed miRNA delivery were associated with alterations in cellular intrinsic fitness, we analyzed translation-linked metabolic activity and proliferative progression following nanowire programming. We conducted SCENITH (single-cell energetic metabolism by profiling translation inhibition) analysis after 48 h of nanowire administration and 48 h of TCR stimulation, initially measuring puromycin incorporation under the no-inhibition control condition (Figure 4A). The quantification of puromycin MFI demonstrated increasing translation output from the miR-29 mimic (~1.2 × 10^6^) and inert-miR (~1.5 × 10^6^) groups to the miR-29-ASO (~1.77 × 10^6^) and ASO-mimic (~1.93 × 10^6^), as the signal progressively augmented. Among the aged T cell cohorts, the All-miR condition exhibited the highest translation output (~2.2 × 10^6^), which was significantly elevated relative to the inert control, indicating an enhanced anabolic capacity during the early effector priming phase.

We then assessed which metabolic pathway supported the higher translation output after multiplexed miRNA delivery by comparing translation output under glycolytic or mitochondrial blockade (Figure 4B). When cells were treated with 2-deoxy-D-glucose (DG), which inhibits glycolysis, translation output converged across groups, with mean puromycin signal remaining within a relatively narrow range of ~0.9–1.2 × 10^6^, indicating that glycolytic blockade broadly constrained protein synthesis and reduced separation between conditions. Under oligomycin, which inhibits mitochondrial ATP production, aged inert and miR-29-treated cells exhibited reduced translation output (~1.0 × 10^6^ and ~0.7 × 10^6^ puromycin MFI, respectively). In contrast, miR-29-ASO, ASO-mimic, and All-miR conditions maintained higher translation levels (~1.3 × 10^6^, ~1.5 × 10^6^, and ~1.8 × 10^6^ MFI, respectively), consistent with an enhanced—potentially combinatorial—capacity to sustain protein synthesis under metabolic stress.

Here, All-miR treated aged T cells showed the highest translation output among the aged groups under oligomycin treatment, indicating that multiplexed miRNA delivery better preserved translation when mitochondrial ATP production was restricted. The derived SCENITH indices were consistent with this interpretation. Glucose dependence, fatty acid oxidation (FAO) and amino acid oxidation (AAO) capacity were not significantly affected by miRNA treatment (Figure 4C). Conversely, clear difference was seen in the balance between mitochondrial dependence and glycolysis capacity (Figure 4D). Relative to the inertmiR group, mitochondrial dependence increased to ~46% in miR-29 group and decreased from ~36% to ~30% in miR-29-ASO, ~25% in ASO-mimic, and ~19% in All-miR (*p* = 0.0005). In the opposite direction, glycolysis capacity increased from about 63% in the inert-miR group to ~70%, ~73%, and ~81% in miR-29-ASO, ASO-mimic, and All-miR (*p* = not significant (ns), 0.0135, and 0.0005, respectively). These results support that multiplexed miRNA delivery shifts activated aged CD8+ T cells toward stronger glycolytic support of translation, rather than causing a non-specific increase across all metabolic pathways. Since glycolytic support is tightly linked to rapid translation, cytokine secretion, and effector differentiation, this metabolic shift aligns with the broader effector-biased phenotype described earlier.

We next examined whether the observed metabolic enhancement was accompanied by increased proliferative capacity, a defining feature of functional T cell response. Carboxyfluorescein succinimidyl ester (CFSE) dilution analysis following 96 h of activation revealed a marked increase in cell division in T cells treated with the ASO-mimic via nanowires, and an even greater effect in cells receiving All-miR delivered via nanowires (Figure 4E). Compared with the inert control group, which displayed a higher proportion of cells in earlier generations (G2 = 23% and G3 = 44%), ASO-mimic-treated T cells showed a redistribution toward intermediate divisions (G3–G4 = 36%–40%). In contrast, All-miR-treated T cells exhibited a pronounced shift toward later generations (G5 = 42% and G6 = 25%), approaching the proliferative profile of young T cells (G5 = 20% and G6 = 62%). Consistent with these data, the fraction of undivided cells decreased substantially across both treatment groups, indicating that restoration of metabolic function supports a more young-like proliferative phenotype.

To objectively quantify these phenomena, standard proliferation metrics were calculated, including the division index (median number of divisions across all cells, including those undivided), the proliferation index (median number of divisions among dividing cells only), the expansion index (overall fold increase in cell number), and the replication index (a comprehensive measure integrating both dividing and non-dividing cell populations). All metrics exhibited a monotonic increase in the sequence: inert, ASO-mimic, and All-miR (Figure S12). Notably, aged CD8+ T cells programmed with All-miR consistently manifested the highest values, in several instances approaching those observed in youthful CD8+ T cells. These findings underscore the potential of multiplex delivery to restore proliferative capacity that is otherwise diminished in aged populations.

### Adoptive transfer of miRNA-programmed aged CD8+ T cells enhances early effector priming *in vivo* against infection

We next assessed whether *ex vivo* programming of aged CD8+ T cells via nanowires results in modified responsiveness *in vivo*. Adoptive transfer of T cells was conducted in mice, with aged recipient mice allocated into four groups (1) saline vehicle only, (2) adoptive transfer of aged CD8+ T cells programmed with inert-miR nanowires, (3) adoptive transfer of aged CD8+ T cells programmed with the multiplexed miRNA cocktail, and (4) adoptive transfer of young CD8+ T cells incubated with inert-miR nanowires as a benchmark (Figure 5A). Following adoptive transfer, the mice were challenged with H1N1 influenza virus to mimic a rapid but non-lethal infection, and blood samples were collected at early (day 4) and later (day 8) time points during the acute phase.

Body weight was used as a coarse physiological readout of disease burden. Across the infection course, recipients of All-miR-programmed aged T cells exhibited a modest but reproducible attenuation of weight loss compared with saline or aged inert-miR controls (Figure 5B). As expected, recipients of young CD8+ T cells displayed the least weight loss, serving here as a positive reference point. Although the magnitude of protection conferred by All-miR aged cells was not as pronounced as that of young donor cells, the improvement relative to aged controls suggests that multiplex programming enhances the early functional contribution of aged CD8+ T cells *in vivo*.

At day 4 post-infection, adoptive transfer of All-miR-programmed aged CD8+ T cells markedly shifted the circulating pool toward an effector-prone phenotype. Flow-cytometric profiling of CD44 and CD62L revealed a decrease in memory fractions by ~15%, and a corresponding expansion of effector subsets from ~10% to ~20% of total CD8+ T cells compared with aged inert-miR controls (Figure 5C). These compositional shifts were accompanied by a coordinated rise in activation markers. The proportion of CD69+ cells increased by 2-fold relative to saline controls and 50% over inert-miR, whereas the CD62L− fraction expanded by around 2-fold (Figure 5D); per-cell expression further indicates that CD69 intensity increased while CD62L intensity decreased. These results suggest enhanced early activation and increased migratory potential in mice receiving All-miR-programmed aged T cells compared with those receiving saline or inert-miR-programmed aged T cells.

These activation and differentiation trends persisted at day 8. The frequency of effector populations nearly doubled compared with aged inert-miR recipients, rising from ~15% to ~30% of total CD8+ T cells (Figure 5E). CD69+ and CD62L− frequencies remained significantly elevated, increasing by 3- and 2-fold, respectively, relative to saline and inert controls (Figure 5F). Correspondingly, per-cell marker intensities followed the same hierarchy: CD69 MFI rose by ~30%, and CD28 MFI by ~30% (Figure 5G). These changes reflect a sustained effector bias and heightened activation state in the circulating CD8+ pool, achieved through *ex vivo* nanowire-mediated programming.

Together, these blood phenotypes (i.e., greater CD69+/CD28+ frequencies, an expanded CD62L− compartment, enrichment of effector population, and modestly reduced weight loss) indicate that *ex vivo* multiplex miRNA programming primes aged CD8+ T cells for earlier and stronger effector responses after influenza challenge relative to saline and aged inert-miR controls. While young donor cells provide an upper benchmark, the All-miR condition consistently shifted activation and subset metrics in the desired direction. These findings suggest that miRNA pre-programming may represent a practical strategy to partially mitigate age-associated CD8+ T cell hypo-responsiveness *in vivo*, although its durability and efficacy in sustained or disease-relevant settings remain to be determined.

### Nanowire-mediated multiplexed miRNA modulation transiently enhances effector priming of aged human CD8+ T cells

To evaluate whether the effects observed in murine CD8+ T cells were recapitulated in a human aged T cell setting, we applied the nanowire delivery platform to primary human CD8+ T cells isolated from ≥65-year-old donors, testing both single and multiplexed miRNA conditions after 48 h activation. Phenotypic analysis using CD45RA/CD45RO alongside CD62L confirmed that nanowire exposure with inert-miR minimally affected post-activation subset distribution compared with the nanowire-only control (Figure 6A). In contrast, transcriptional-program-targeting miRNAs caused clear, directional shifts that mirrored the murine results. Delivery of the miR-29 mimic maintained CD62L+ populations, while miR-29-ASO shifted the balance toward CD45RO+CD62L− effector-memory-like subsets. The multiplexed All-miR combination had the most pronounced effect, reducing the CD45RA+CD62L+ fraction by about 10% and increasing the CD45RO+CD62L− fraction by ~10% compared with aged inert-miR conditions, indicating an accelerated transition into effector-competent states.

Activation-associated markers further supported this trend toward a higher activation state with combinational and multiplexed miR delivery. Although miR-29 and miR-29-ASO alone did not significantly change the activation phenotype, the frequencies of CD69+ and CD28+ cells increased notably in All-miR groups compared with inert controls (by approximately 25% and 20%, respectively), and the CD62L− fraction grew by 10% (Figure 6B). Per-cell expression also reflected these frequency changes: CD69, CD28, and CD44 levels rose by around 50%, while CD62L expression fell by more than 50% relative to the inert control (Figure 6C). The consistency between frequency and MFI in early activation (CD69), co-stimulation (CD28), and homing/differentiation markers (CD62L/CD44) suggests a comprehensive elevation of the activation set point in aged human CD8+ T cells following multiplex miRNA programming.

Tracking T-bet expression after activation further supported this trend. A gradual increase was seen across conditions (All-miR > ASO-mimic > miR-29-ASO > inert/device controls), with the lowest levels in the miR-29 mimic group (Figure 6D). Quantitatively, T-bet MFI roughly doubled in the All-miR condition compared with inert-treated cells. These findings suggest that pre-activation miRNA programming establishes a lasting transcriptional tone that persists during early human T cell activation. Functional assessments align with these phenotypic and transcriptional observations. The percentage of IFN-γ+ and perforin+ cells increased by about 20% and 10%, respectively, with All-miR treatment compared with controls (Figure 6E). Per-cell MFI for these effector molecules also increased significantly: by approximately 30% for IFN-γ, 65% for perforin, and 90% for granzyme B (Figure 6F). Importantly, analysis of activation dynamics (Figures S13A–S13D) and functional responses (Figures S14A–S14C) across different subsets showed these improvements were widespread across all differentiation states, including central memory T cells (T_CM_), effector memory T cells (T_EM_), and effector T cells (T_Eff_), despite only small changes in their frequencies. Notably, effector T cells, whose numbers remained stable, showed significant per-cell increases in granzyme B, IFN-γ, and perforin expression. This pattern indicates that multiplex miRNA programming boosts the functional capacity of existing effector T cells rather than causing excessive terminal differentiation. Since over-differentiation can lead to exhaustion or reduced lifespan, maintaining a balanced subset distribution while increasing per-cell response likely offers a more effective rejuvenation approach. Overall, these quantitative enhancements demonstrate that nanowire-enabled multiplex miRNA delivery increases both the percentage of responsive aged human CD8+ T cells and their per-cell effector molecule content, effectively mirroring the functional rejuvenation observed in mouse models.

We next investigated whether these effects extended beyond the immediate 48 h activation window. The longitudinal data support a predominantly early effect rather than a uniformly sustained difference across the entire 12-day period. Regarding differentiation state, the combined effector-like compartment (comprising T_Effector Memory_ and T_Effector_ cells) was higher in the All-miR group on day 2 (~72% compared with 55% in the inert group) and day 4 (~72% versus 60%), although this pattern was not maintained at later time points. By day 8, the inert group had largely caught up and was slightly higher (~76% versus 71%), and by day 12, both groups exhibited similar levels (both approximately 98%, not significant) (Figure 6G). T-bet expression displayed a similar temporal pattern: it was elevated in the All-miR group at day 2 (~20,000 versus 11,000 MFI) and day 4 (~30,000 versus 16,000 MFI) but showed no difference at days 8 or 12 (Figure 6H). These data suggest that multiplexed miRNA delivery enhances early post-activation effector priming; however, this enhancement was not uniformly maintained throughout the subsequent culture period. Effector readouts further revealed a decline in the effects of miRNA modulation over time, with the most pronounced differences again observed at the earlier time points. For granzyme B, while the frequency of granzyme B-secreting cells remained relatively stable, the MFI per cell was higher in the All-miR group at day 2 (~60% increase) and day 4 (~0.52 versus 0.36) but converged thereafter (Figure 6I). The most notable early differences were observed for IFN-γ and perforin. IFN-γ+ cells were more prevalent in the All-miR group at day 2 (~60% versus 20%) and day 4 (~20% versus 4%), with normalized IFN-γ MFI also increased at day 2 (by approximately 60%) and day 4 (~0.9 versus 0.6) but not at later time points (Figure 6J). Similarly, perforin-positive cells were higher in the All-miR group at day 2 (~56% versus 32%) and day 4 (~48% versus 25%), whereas normalized perforin MFI was higher at day 2 (~30% increase) but showed no significant differences from day 4 onward (Figure 6K). In summary, these longitudinal data indicate that multiplexed miRNA delivery amplifies early effector-associated output following activation, particularly during the first 2–4 days. Nonetheless, most differences diminished by day 8 and were virtually absent by day 12, suggesting that these effects represent transient activation-related modulation rather than a permanent reprogramming of cellular state.

Because increased activation and effector priming may lead to dysfunction with repeated stimulation, we investigated whether the quick activation and effector programming induced by miRNA delivery resulted in early dysfunction or cell loss. To do this, we created a multi-restimulation schedule that mimics chronic T cell stimulation, a condition known to lead to exhaustion and apoptosis (Figure S15A). Long-term monitoring of cell numbers showed that All-miR-programmed aged CD8+ T cells maintained proliferation for a longer period compared with inert-treated controls (Figure S15B). Both groups expanded similarly until day 4; however, the inert group reached its peak early and declined sharply by day 8, whereas the All-miR group continued proliferating and maintained a higher number of viable cells. On day 8, the All-miR-programmed cells had about twice as many live cells as the inert group, indicating less apoptosis and prolonged proliferative capacity. We then assessed the specific inhibitory markers T cell immunoglobulin and mucin domain-containing protein 3 (TIM-3) and lymphocyte activation gene 3 (LAG-3) at the day 8 endpoint. TIM-3 frequency and TIM-3 MFI were both lower in the All-miR group than in inert controls, while LAG-3 frequency was not increased and LAG-3 MFI was reduced (Figures S15C–S15F). These findings support a measured interpretation that multiplexed miRNA delivery enhanced activation and effector-associated function without producing an overt increase in the specific inhibitory markers examined within the tested time window.

### Multiplexed miRNA delivery enhances aged CD8+ T cell activation across healthy donors and cancer patients

To determine whether multiplexed miRNA modulation remained effective across clinically distinct aged human donor backgrounds, we applied nanowire-mediated delivery of inert-miR or the multiplexed miRNA cocktail to primary CD8+ T cells isolated from three groups of older donors: healthy older adults, non-Hodgkin lymphoma (NHL) survivors more than 2 years after completion of chemoimmunotherapy without evidence of relapse, and older donors with newly diagnosed solid tumors who had not yet received systemic cancer-directed therapy (ST) (Figure 7A). These cohorts represent different immunologic contexts, including age-associated immune decline alone, prior therapy-associated immune remodeling, and cancer-associated immune dysfunction, and therefore provided an opportunity to assess whether multiplexed miRNA delivery produced consistent effects across heterogeneous donor backgrounds.

Across all donor groups, multiplexed miRNA delivery increased T-bet expression relative to inert-miR controls, although the magnitude of the response varied between individual donors (Figure 7B). The most consistent increases were observed in healthy aged donors, while NHL and ST donors also showed upward shifts, albeit with greater inter-donor variability. These data indicate that multiplexed miRNA delivery can enhance an effector-associated transcriptional program in aged human CD8+ T cells across distinct donor backgrounds, while also highlighting that the magnitude of this response is donor dependent. Given the small number of donors in each category, these cohort-level differences are most appropriately interpreted as evidence of biological variability rather than as definitive distinctions between disease groups.

Activation-associated markers showed a similarly consistent directional pattern, albeit with donor-specific amplitude and variability across cohorts. The fraction of CD69+ cells increased in all donors following multiplex miRNA delivery, with the most prominent gains observed in NHL1 (~35%) and the healthy donors (~30%–35%). In contrast, NHL2 and ST 1–2 exhibited smaller yet statistically significant increases of approximately 15%–25% (Figure 7C). Additionally, CD69 MFI increased markedly, ranging from approximately 25% in ST donor 2 to nearly 2-fold in NHL donor 1 (Figure 7D). These findings suggest that multiplex miRNA delivery enhances early immune activation, even within immune systems affected by chronic inflammation or prior oncologic treatment, although with variable dynamic ranges. The greater variation seen in NHL and ST donors probably indicates differences between patients in their baseline TCR signaling thresholds and lingering immune issues from previous treatments. In contrast, healthy aged donors showed more consistent improvements. The co-stimulatory receptor CD28, which experiences a progressive decline during aging and prolonged antigen exposure, was similarly upregulated following multiplex programming (Figures 7E and 7F). The frequencies of CD28+ cells increased across all donors, with the most substantial increases observed in healthy 1–2 and ST 1, alongside more modest yet statistically significant changes in NHL donors. This variation may be attributable to the higher basal CD28 expression in the two NHL donors. CD28 expression measured by MFI increased by approximately 30% on average across the cohorts, attaining comparable post-activation levels among all patient groups despite differing initial baselines. After All-miRNA reprogramming, all groups converged toward a similar activation plateau, implying that the combined miRNA cocktail harmonizes activation capacity across diverse immune backgrounds.

Beyond activation-associated markers, multiplex miRNA programming significantly enhanced cytotoxic effector function across all groups (Figures 7G and 7H). Baseline perforin+ frequencies were notably lower in NHL and ST donors compared with healthy donors (~40%–60% versus ~80%–90%). After multiplex miRNA delivery, all groups showed increased perforin production, with perforin+ cell frequencies rising by approximately 20% in NHL and ST donors, and about 10% in healthy donors. Perforin MFI also increased significantly for NHL and ST donors, ranging from 50% to 100% compared with their respective inert-miR control. Importantly, these improvements narrow the baseline gap between cancer survivors and healthy donors, as nanowire-mediated miRNA delivery brings NHL/ST samples closer to the healthy benchmark in both frequency and per-cell expression. This suggests that previously therapy-conditioned T cells can re-establish cytolytic potential when treated with the multiplexed miRNA cocktail. These results are especially relevant because diminished perforin content is a major feature of impaired cytotoxic function in aged and cancer-affected T cells. Thus, increasing both the proportion of perforin+ cells and the amount of perforin expressed per cell suggests that multiplexed miRNA delivery expands the cytotoxic pool while also increasing the functional intensity of individual responder cells.

Analysis of other effector molecules revealed that granzyme B (Figures S16A and S16B) and IFN-γ (Figures S16C and S16D), which were already highly expressed at baseline in most donors, still showed a significant increase in the number of secreting cells and/or per-cell intensity. The degranulation marker CD107a also increased in both frequency (20%–40%) and expression intensity (~40%–60%) across donors (Figures S17A and S17B), indicating that the effect of the multiplexed cocktail was not limited to one cytotoxic molecule, but extended more broadly across effector-associated outputs. These trends confirm with the observation that multiplexed miRNA delivery enhances both the number of functionally engaged CD8+ T cells and the magnitude of their effector response.

Taken together, these results show that nanowire-mediated multiplex miRNA delivery enhances activation-associated and cytotoxic readouts in aged human CD8+ T cells across healthy donors and donors with current or prior cancer. Although the magnitude of improvement varied across individual donors, the direction of change was broadly consistent across cohorts, supporting the robustness of the approach in heterogeneous human samples. At the same time, the present data are most appropriately interpreted as evidence of enhanced *ex vivo* functional priming rather than full restoration of youthful function, and larger donor studies will be needed to define the sources of inter-donor variability and the relative responsiveness of specific clinical subgroups.

## DISCUSSION

Aging significantly remodels the immune system and reduces the ability of CD8+ T cells to respond effectively to infection, vaccination, and cancer. Impaired activation, reduced transcriptional flexibility, and diminished proliferative capacity collectively weaken the function of aged T cells. miRNAs offer a promising approach to modulate these age-associated defects because they can influence multiple regulatory pathways simultaneously. However, efficient delivery into primary aged T cells without pre-activation has remained a major challenge. In this study, we developed a nanowire-based multiplex miRNA delivery platform that transiently modulates aged CD8+ T cells across several functional levels. By combining miRNAs that influence transcriptional regulation, signaling sensitivity, metabolism, and co-stimulatory state, this multiplexed approach produced enhanced effects than single miRNA delivery alone. In both murine and human systems, multiplex miRNA delivery enhanced early activation-associated responses, promoted effector-biased differentiation, increased effector molecule production, and improved metabolic fitness and proliferative progression. *In vivo*, *ex vivo-*programmed aged CD8+ T cells exhibited enhanced early effector priming following adoptive transfer. However, these effects were assessed within a defined early window and may represent transient or partial reprogramming rather than durable lineage reconstitution, which remains to be established. Consistent with this time frame, the observed gains were not accompanied by a detectable increase in the inhibitory markers examined. Validation in aged human CD8+ T cells from older donors, including healthy individuals and donors with current or prior cancer, further showed that this multiplexed miRNA strategy consistently enhanced activation-associated and cytotoxic readouts across diverse donor backgrounds, supporting translational potential while also highlighting the need for longer-term studies to define durability and potential late-phase regulatory constraints.

**Table:** Healthy aged donors

PBMC donors	Age	Sex

1	78	male
2	77	female
3	72	male
4	75	female

Several important questions remain. Although cytokine production and other functional outputs were significantly improved, some responses still did not fully reach the levels observed in young counterparts, indicating that not all age-associated barriers were overcome by miRNA modulation alone. In addition, the longitudinal analyses showed that the effects of multiplexed miRNA delivery were strongest during the early post-activation period and progressively diminished over time, with many readouts showing marked convergence by later time points. These findings indicate that the treatment modulates early activation and effector trajectories without inducing durable or permanent rewiring of aged CD8+ T cells. Rather, the data support a model of transient or potentially sustained post-activation modulation within the observed window, as opposed to stable, long-term cell-state reprogramming.

While the selected miRNAs are individually linked to key aspects of T cell biology, the precise molecular targets and higher-order network interactions underlying their combined effects remain to be fully defined. The present *in vivo* studies were limited to early post-transfer responses, and further work will be needed to determine the persistence of these effects, their influence on memory formation, and their performance in chronic infection or tumor settings. Future studies may also evaluate whether multiplex miRNA delivery can be combined with complementary strategies, such as cytokine support, metabolic interventions, or checkpoint-targeted approaches, to further enhance the magnitude and durability of aged T cell responses. Overall, these findings establish multiplex miRNA nanowire programming as a promising *ex vivo* strategy to enhance the functional capacity of aged CD8+ T cells and support improved immune responsiveness in older and immunocompromised populations.

## METHODS

### Ethics oversight

The research presented in this report adheres to pertinent ethical standards and strictly follows the guidelines outlined in the Guide for the Care and Use of Laboratory Animals by the National Institutes of Health. The protocols received review and approval from the Institutional Animal Care and Use Committees at Georgia Institute of Technology (protocol no. A100365). Additionally, the human studies included in this report conform to relevant ethical standards and are conducted under protocols approved by the Institutional Review Board (IRB) of Emory University School of Medicine, with IRB approval numbers IRB00065989 for human diffuse large B cell lymphoma (DLBCL) samples and IRB00095411 for non-lymphoma samples.

**Table:** Aged NHL survivors

PBMC donors	Age	Sex

5	70	male
6	73	male

**Table:** Aged donors with newly diagnosed solid tumors

PBMC donors	Age	Sex

7	73	female
8	77	male

### Mice

C57BL/6 mice were procured from Jackson Laboratory (strain code 000664). Female mice classified as young were aged between 3 and 6 months, whereas aged female mice ranged from 18 to 24 months. All subjects were housed under pathogen-free conditions at the Georgia Institute of Technology. Spleens were harvested, dissociated by forcing through a 70 μm cell strainer (Corning, 352350) using a sterile plunger, and the resultant cell suspension was incubated in red blood cell lysis buffer for 5 min at room temperature to eliminate erythrocytes and isolate splenocytes. Pan CD8+ T cells were subsequently isolated via negative magnetic selection, as described in detail later.

### Human cancer patients

Patients diagnosed with DLBCL or other non-lymphoma malignancies were prospectively identified and consented to participate in the study. Whole-blood samples were collected with informed consent from all participants in accordance with the IRB protocols of Emory University School of Medicine. Samples from patients with DLBCL were obtained at least 24 months following the completion of frontline treatment with chemoimmunotherapy (e.g., R-CHOP [rituximab, cyclophosphamide, doxorubicin, vincristine, prednisone]) and prior to any evidence of disease relapse. Additionally, samples from patients with non-lymphoma cancers were collected prior to initiation of any systemic chemotherapy or immunotherapy. Samples from healthy aged donors were obtained from Stemcell Technologies.

### Nanowire wafer fabrication

Wet thermal oxidation: for the fabrication of nanowire devices, a prime silicon wafer measuring 100 mm in diameter, with a P/B (1–0-0) orientation, resistivity of 1–10 Ω · cm, and thickness of 525 ± 25 μm, was procured from WaferPro (SKU X040145000). This wafer was single-side polished and featured two semi-flats, with a wet thermal oxide layer of 5,000 Å ± 5% on both sides. Photoresist deposition: the silicon wafers were processed using the Gamma Automatic Coat-Develop Tool at the Cornell NanoScale Science and Technology Facility (CNF). Initially, the wafers were primed with deep-ultraviolet (DUV) 42P Primer (Brewer Science) to achieve a primer coating of 60 nm. Subsequently, UVN2300 Negative Photoresist (Rohm & Haas Electronic Materials) was coated onto each wafer at 3,000 rpm for 60 s, followed by baking at 100°C to attain a thickness of approximately 500 nm. Advanced Semiconductor Materials Lithography (ASML) DUV Stepper 4× lithography: the wafers underwent DUV photolithography in Advanced Semiconductor Materials Lithography (ASML PAS 5500/300 C DUV Wafer Stepper at CNF, with an exposure dose of 35.00 mJ cm^−2^. A quartz photomask (Photronics) featuring dot arrays of 1.2 μm diameter and 12.0 μm spacing was employed, with a 4× reduction in feature dimensions. Post-exposure development was carried out with AZ 726 MIF developer (Merck) at 110°C for 60 s in the Gamma Tool. Silicon dioxide mask etching: nanowire patterns in the exposed photoresist were transferred onto a silicon dioxide mask via anisotropic reactive ion etching (RIE) using the Plasmatherm SLR 720 MF system at the Micro/Nano Fabrication Facility of the Institute for Electronics and Nanotechnology (IEN) at Georgia Tech. The process parameters included a pressure of 5 mTorr, Ar flow of 5 sccm, CO_2_ flow of 28 sccm, C_4_F_8_ flow of 15 sccm, RF1 power of 40 W, RF2 power of 800 W, and an etching duration of 5 min. Deep silicon RIE etching: to transfer nanowire patterns into the silicon substrate and fabricate high-aspect-ratio nanopillars, deep RIE was performed using the Surface Technology Systems (STS) High Resonance Magnet (HRM) inductively coupled plasma etcher at the IEN at the Georgia Institute of Technology. The recipe is composed of passivation and etching steps: during passivation, parameters included 2 mTorr pressure, 200 sccm C_4_F_8_, RF power of 1,200 W, and a processing time of 2 s per cycle. During etching, conditions were 2 mTorr pressure, 200 sccm SF_6_ and 20 sccm O_2_, RF power of 2,000 W, platen power of 50 W, and 4 s per cycle. A total of 40 cycles were executed. Postprocess, vertical nanopillar arrays measuring 4 μm in height, with a diameter of 600 nm and profile angles exceeding 85°, were attained. Isotropic etching: to generate nanowire structures from the nanopillars, isotropic etching was conducted on the silicon wafers utilizing the Unaxis 790–10 RIE system at IEN, Georgia Tech. The etching recipe consisted of a pressure of 50 mTorr, O_2_ flow of 10 sccm, CF_4_ flow of 40 sccm, RF1 power of 250 W, and an etching duration of 20 min. Following this process, vertical nanowire arrays with a height of approximately 3.5 ± 0.1 μm, tip diameters of 20 nm, and basal diameters of 150 nm were produced.

**Table:** 

Name	5′ to 3′ sequence	3′ modification	Purification	Base modification	Vendor

miR-29a-mimic, guide strand	UAGCACCAUCUGAAAUCGGUUA	6-FAM	HPLC	N/A	Sigma-Aldrich
miR-29a-mimic, passenger strand	ACUGAUUUCUUUUGGUGUUCAG	–	HPLC	N/A	Sigma-Aldrich
miR-29a-ASO	UAACCGAUUUCAGAUGGUGCUA	6-FAM	HPLC	2′-O-methyl RNA	Sigma-Aldrich
miR-130a-mimic, guide strand	CAGUGCAAUGUUAAAAGGGCAU	cyanine5	HPLC	N/A	Sigma-Aldrich
miR-130a-mimic, passenger strand	GCUCUUUUCACAUUGUGCUACU	–	HPLC	N/A	Sigma-Aldrich
miR-150-mimic, guide strand	UCUCCCAACCCUUGUACCAGUG	cyanine5.5	HPLC	N/A	Sigma-Aldrich
miR-150-mimic, passenger strand	CUGGUACAGGCCUGGGGGACAG	–	HPLC	N/A	Sigma-Aldrich
miR-181a-mimic, guide strand	AACAUUCAACGCUGUCGGUGAGU	cyanine3	HPLC	N/A	Sigma-Aldrich
miR-181a-mimic, passenger strand	ACCAUCGACCGUUGAUUGUACC	–	HPLC	N/A	Sigma-Aldrich
inert (control) mimic, guide strand	UAAAAAUCGCGUGGAUUAAUG	6-FAM	HPLC	N/A	Sigma-Aldrich
inert (control) mimic, passenger strand	UUAAUUUACGCGGUUUUUAUU	–	HPLC	N/A	Sigma-Aldrich

### Spin coating and wafer dicing

To section the silicon wafers into individual square-shaped 1 cm × 1 cm nanowire chips, the wafers were initially spin-coated with MEGAPOSIT SPR220 photoresist at 1,500 rpm for 30 s using a Karl Suss RC8 Spinner and subsequently baked at 115°C for 10 min to achieve a photoresist layer thickness of 10 μm. This procedure was conducted at the IEN, Georgia Institute of Technology. Following this, the wafers were diced into 1 cm × 1 cm nanowire chips utilizing a Disco-DAD3360 automatic dicing saw. The chips underwent a systematic cleaning process: initially cleaned with 100% acetone, then with 100% methanol, and finally with 100% isopropyl alcohol to effectively eliminate any residual photoresist.

### Silane-PEG-NHS functionalization

For the surface modification of silicon nanowire chips, oxygen plasma treatment was performed using the Harrick Plasma PDC-001-HP Plasma Cleaner with a pressure of 400 mTorr and a power of 45 W for a duration of 3 min. This process was employed to achieve a clean chip surface with hydroxyl groups. Subsequently, a silane-functionalized NHS moiety (silane-PEG-NHS, NANOCS, catalog: PG2-NSSL-5k) was conjugated onto the oxygen plasma-treated silicon nanowire surface to establish a covalent bond between the residual oxide from silicon dioxide and the silane. This procedure effectively attached the NHS group to the nanowire surface via an amide conjugation linker. Additionally, PEG chains linking the two functional groups were utilized, as they serve to suppress non-specific binding of charged molecules to the modified surfaces and mitigate excessive PEI coating on the nanowire surfaces.

### PEI functionalization

Branched PEI (weight-averaged molecular mass, ~25,000; Sigma-Aldrich, 9002–98-6) was reconstituted at 10% w/v in deionized water and adjusted to ~pH 8.0 with HCl. Next, nanowire chips functionalized with silane-PEG-NHS were spin-coated with PEI solutions at 1,000 rpm for 30 s using a Karl Suss RC8 Spinner to achieve uniform PEI coverage on the nanowire surfaces. Spin-coated nanowire chips were then incubated for 30 min at room temperature to allow NHS ester bioconjugation with amines. All nanowire chips were then thermally annealed at 70°Con a hot plate for 5 min to improve the stability of PEI functionalization, then washed with ethanol and deionized water, and dried under a nitrogen flow to remove physically adsorbed impurities. After conjugation, residual PEI was rigorously rinsed to prevent unintended cytotoxicity from free PEI. The nanowire chips were subsequently used for transfection by placing them in 24-well plates.

### SEM imaging

SEM of the nanowire surface was conducted utilizing the Hitachi SU-8230 SEM instrument at the IEN/IMat Materials Characterization Facility (MCF), Georgia Institute of Technology.

### Oligonucleotide sequences and modifications

ASO and mimic sequences are listed below. RNA oligos were custom synthesized with fluorescent tag at the 3′ end and purified by high-performance liquid chromatography (HPLC) (Sigma). The miRNA mimics were annealed by mixing the guide and passenger strands in 1× small interfering RNA (siRNA) buffer (Dharmacon), denaturing at 95°C for 5 min and slowly cooling to room temperature.

### SA-β-gal staining

To validate the aged phenotype of murine CD8+ T cells, primary young and aged murine CD8+ T cells were analyzed either immediately after isolation (day 0) or after 48 h activation with anti-CD3/anti-CD28 Dynabeads at a 1:1 bead-to-cell ratio. Cells were stained with an SA-β-gal flow cytometry reagent (Invitrogen, C10840) according to the manufacturer’s instructions. Unstained controls were included for each condition. After staining, cells were washed, resuspended in fluorescence-activated cell sorting (FACS) buffer, and analyzed by flow cytometry. SA-β-gal signal was quantified as MFI within live CD8+ T cells.

### Nanowire-miRNA-treated CD8+ T cell cultures

A 50 μL suspension of miRNA and oligonucleotide material in PBS at a concentration of 100 nM was prepared and deposited onto the surface of the nanowires. Subsequently, the oligonucleotide was incubated for 2 h in an ambient environment at room temperature in the dark, facilitating the deposition of the oligonucleotide onto the wafer surface. Following the coating process, 500,000 CD 8 + T cells, enriched via negative magnetic selection (STEMCELL Technologies [19258 (human) and 19858 (mouse )]) and suspended in approximately 60 μL of serum- free media, were seeded onto the oligonucleotide- coated nanowires without disrupting the meniscus formation at the edge of the wafer or making contact with the wafer surface. The T cells were then incubated on the wafer for 15 min in darkness and subsequently centrifuged at 50 × *g* for 5 min with low acceleration (1) and deceleration (0). Primary human or murine CD8+ T cells were cultured on PEI-functionalized nanowires for up to 48 h within a CO_2_ incubator maintained at 37°C, in the presence of complete medium supplemented with recombinant IL-2 (5 ng/mL) (human: Gibco, 200–02; mouse; Gibco, 212–12), IL-7 (5 ng/mL) (human: Gibco, 200–07; mouse: Gibco, 217–17), and IL-15 (5 ng/mL) (human: Gibco, 200–15; mouse: Gibco, 210–15). The complete medium consisted of RPMI 1640 (Gibco, 11875093) as the basal medium, supplemented with 10.0% fetal bovine serum (FBS) (Gibco, 26140079), 1.0% penicillin-streptomycin- neomycin antibiotic mixture (Gibco, 15640055), 1.0% minimum essential medium (MEM) non-essential amino acids solution (Gibco, 11140050), 1.0% GlutaMAX supplement (Gibco, 35050061), 1.0% of 100 mM sodium pyruvate (Gibco, 11360070), and 0.1% of 2-mercaptoethanol (Gibco, 21985023). The cells were cultured for two days in a CO_2_ incubator at 37°C. To investigate the effects of individual and combined delivery of miRNAs on CD8+ T cell viability following activation, 500, 500,000 murine or human CD8+ T cells were transfected onto PEI-functionalized nanowires for 48 h. Activation was subsequently performed with anti-CD3 and anti-CD28 Dynabeads (human: Gibco, 11161 D; mouse: Gibco, 11456 D) at a bead-to-cell ratio of 1:1 for an additional 48 h after nanowire transfection.

### Benchmarking experiments

For benchmarking experiments, 100,000 young or aged murine CD8+ T cells (without pre-activation using anti-CD28/anti-CD3 beads) were incubated with 40 μL of 25 μM 6-FAM-miR-29-mimic per group. 6-FAM emits light similar to FITC in the spectrum. We selected 1 μM to ensure a detectable signal even in conditions with low transfection efficiency. T cells were incubated on nanowires for 48 h before flow cytometry.

Nucleofection involved suspending 100,000 CD8+ T cells in 100 μL of Mouse T Cell Nucleofector Kit solution (Lonza, VPA-1006). To this, 6-FAM-miR-29 was added and mixed thoroughly. The suspension was transferred into the Nucleofector cuvette (Lonza, AAB-1001) of the Nucleofector II/2b system. Cells were nucleofected using program V-024. Afterward, 100 μL of pre-warmed Opti-MEM was added, and cells were transferred to 24-well plates to recover for 15 min at 37°C. Finally, complete RPMI was added, and the T cells incubated for 48 h at 37°C in a 5% CO_2_ incubator before flow cytometry analysis.

### Endocytic inhibition assay

To assess whether nanowire-mediated miRNA delivery depended on classical endocytic uptake, primary aged murine CD8+ T cells were treated with dynasore hydrate (Sigma-Aldrich, D7693) and chlorpromazine hydrochloride (Sigma-Aldrich, C8138) at final concentrations of 40 and 20 μM, respectively, for 15 min at 37°C. Vehicle-only controls were included in parallel. Following pre-treatment, cells received 6-FAM-miR-29 under one of four delivery conditions: PEI-functionalized nanowires, nucleofection, Lipofectamine-mediated transfection, or 2D culture without a delivery reagent. For nanowire-mediated delivery, 6-FAM-miR-29 was loaded onto PEI-functionalized nanowires as described above. Nucleofection and Lipofectamine groups were prepared according to the manufacturer’s instructions using the same 6-FAM-miR-29 cargo input. For the 2D control, 6-FAM-miR-29 was added directly to cells cultured on standard tissue-culture plastic in the absence of a carrier. Cells were incubated for 4 h after delivery in the presence of dynasore and chlorpromazine, washed with PBS, stained with viability dye and anti-CD8 antibody, and analyzed by flow cytometry. Delivery efficiency was quantified as the percentage of 6-FAM+ cells within the live CD8+ T cell gate.

### Confocal imaging of T cell penetration by nanowire

To visualize nanowire penetration inside the CD8+ cells and the subsequent release of miRNA into the cell, isolated young or aged primary mouse CD8+ T cells were seeded on PEI-functionalized nanowires whose surface had been seeded with 6-FAM-miR-29-mimic. After centrifugation at 50 × *g* and incubation for 6 h at 37°C, the samples were harvested and washed 3× with PBS and fixed with 4% paraformaldehyde (Thermo Scientific Chemicals, J61899.AK) for 30 min. Following this, the nanowire samples were washed 3× with PBS before permeabilizing with 1% Triton X-100 (Invitrogen, HFH10) in PBS for 30 min. For surface staining, the cells were incubated with 0.5% anti-CD8a Alexa Fluor 647 mAbs (Invitrogen, 740029TP647) in PBS containing 1% BSA for 1 h at 4°C in the dark. The samples were washed 3× in 1% BSA PBS following surface staining and incubated with 1:500 DAPI solution for nucleus staining and then washed 3× in PBS before imaging on a ZEISS LSM 900 confocal microscope (ZEISS).

### Flow cytometry

For flow cytometry analysis, cells were spun down at 400 × *g* to achieve a single-cell suspension. Cells were subsequently washed 3× with PBS before live/dead staining for half an hour in PBS. Next, the cells were washed 3×, resuspended in FACS buffer, and incubated with surface-targeting antibodies for 1 h on ice in the dark. For intracellular staining, the cells were washed 3× in FACS buffer before being fixed and permeabilized with Foxp3/Transcription Factor Fixation/Permeabilization kit (Invitrogen, 00–5521-00) according to the manufacturer’s instructions. Following this, the cells were stained with intracellular-targeting antibodies overnight on ice in a dark environment and were washed 3× with FACS buffer before being recorded and analyzed on a 5-Laser Cytek Aurora System (Cytek) using SpectroFlo software. Flow cytometry analysis was performed using the FlowJo v.10 (Treestar) software.

A list of antibodies, clones and vendors are described below: mAbs anti-CD3 (OKT3, UCHT1), anti-CD8a (53–6.7, RPA-T8), anti-CD4 (RM4–4, RPA-T4), anti-CD45.1 (A20), anti-CD45.2 (104), anti-CD45RA (14.8), anti-CD45RO (UCHL1), anti-CD25 (PC61, M-A251), anti-CD95/Fas (Jo2, DX2), anti-CD62L (MEL-14, DREG-56), anti-CD197/CCR7 (4B12, 3D12), anti-CD44 (IM7), anti-CD28 (37.51, CD28.2), anti-CD69 (H1.2F3, FN50), anti-TNF-α (MP6-XT22, MAb11), anti-CD279/PD-1 (J43, J105), anti-CD223/LAG-3 (3DS223H), anti-TIM-3 (RMT3–23), anti-T-bet (4B10), anti-Eomes (Dan11mag, WD1928), anti-KLRG1 (MAFA), anti-CD127 (A7R34, HIL), anti-CD27 (LG.7F9), anti-IFN-γ (XMG1.2, 4S.B3), anti-perforin (dG9, eBioOMAK-D), anti-granzyme B (NGZB), anti-CD107a (eBioH4A3, 1D4B), anti-puromycin (EPR27218–173) and Fixable Viability Dye were purchased from Abcam, BioLegend, eBioscience, Invitrogen, BD Biosciences, or R&D Systems.

### SCENITH metabolic profiling

Metabolic profiling was performed using SCENITH on primary aged murine CD8+ T cells after nanowire-mediated miRNA delivery and 48 h of TCR stimulation. Briefly, aged murine CD8+ T cells were incubated on PEI-functionalized nanowires for 48 h and then activated with anti-CD3/anti-CD28 Dynabeads for the subsequent 48 h. Cells from each miRNA condition were then divided into DMSO control (Co), DG, oligomycin A (O), or combined DG + oligomycin A (DGO) treatment groups. DG (Sigma-Aldrich, D6134) and oligomycin A (Sigma-Aldrich, 75351) were used at final concentrations of 100 mM and 1 μM, respectively, and incubated with cells for 15 min at 37°C. Cells were then pulsed with puromycin (Thermo Scientific Chemicals, J67236.8EQ) for 30 min, washed with PBS, stained with a viability dye, and prepared for flow cytometry analysis. Total biogenic capacity was determined from puromycin incorporation under control conditions. Glucose dependence, FAO and AAO capacity, mitochondrial dependence, and glycolysis capacity were calculated from puromycin signal across control and inhibitor-treated groups according to the standard SCENITH workflow.

### Adoptive single transfer and H1N1 virus infection

Splenocytes were obtained from young or aged male C57BL/6 mice (Jackson, no. 000406), and CD8+ T cells were isolated via previously described negative magnetic selection. The purified CD8+ T cells were then incubated with nanowires presenting either control inert-miR or target miR-29-ASO + miR-130-mimic + miR-150-mimic + miR-181a-mimic for a duration of 48 h. Subsequently, 500,000 nanowire-incubated CD8+ T cells were adoptively transferred intravenously into aged male C57BL/6 recipient mice 1 day prior to infection with live H1N1 influenza virus. The mice were intraperitoneally infected with 500 50% tissue culture infectious doses (TCID_50_) virus particles in 50 μL PBS 24 h subsequent to the adoptive transfer of nanowire-treated CD8+ T cells. The mice were then monitored and weighed every 2 days post-infection. On days 4, 8, and 12, peripheral blood was collected from the submandibular vein for assessment of T cell activation status. The mice were euthanized on day 12, and splenocytes were harvested for further analysis.

### *In vitro* mouse T cell proliferation and cytokine response analysis

Nanowire-incubated CD8+ T cells were resuspended in PBS at a concentration of 1 × 10^7^ cells per milliliter and subsequently stained with CFSE proliferation dye (Thermo Fisher, no. C34554) at a dilution of 1:1,000 for a duration of 5 min at room temperature in darkness. This was followed by quenching with an equal volume of FBS. The nanowire-modified CD8+ T cells, stained with CFSE, were then resuspended in complete media and stimulated with anti-CD3 and anti-CD28 Dynabeads (Gibco, 11161D for human and 11456D for mouse) at a 1:1 bead-to-cell ratio within a 48-well flat-bottom plate, incubated for 48 h at 37°C. To facilitate intracellular cytokine detection, 3 μg/mL of brefeldin A (Invitrogen, 00–4506-51) was added to the culture media 6 h prior to cell collection. The cells underwent fixation and were stained with both surface and intracellular antibodies for flow cytometry analysis of cytokine secretion utilizing the Cytek Aurora System (Cytek). Additionally, culture media was collected and subjected to analysis via ELISA.

### Longitudinal post-activation analysis of human CD8+ T cells

For extended analysis of aged human CD8+ T cell responses, primary aged human CD8+ T cells were transfected with PEI-functionalized nanowires using inert-miR or All-miR for 48 h, then activated with anti-CD3/anti-CD28 Dynabeads at a 1:1 bead-to-cell ratio for 48 h. Dynabeads were removed after the initial 48 h activation period, and cells were maintained in culture until day 12. Samples were collected on days 2, 4, 8, and 12 after activation for flow cytometry analysis. Both frequency and MFI were quantified at each time point.

### Repeated restimulation/exhaustion assay

Primary aged human CD8+ T cells were transfected on PEI-functionalized nanowires for 48 h and then subjected to serial anti-CD3/anti-CD28 Dynabeads stimulation. Following the initial 48 h activation period, cells were re-stimulated every 48 h with fresh Dynabeads for a total of four rounds of stimulation before endpoint analysis with flow cytometry. Live cell counts were measured at the indicated time points to assess growth kinetics.

### Statistical analysis

Statistical analysis was performed using GraphPad Prism 10 software. Data were analyzed using an unpaired two-tailed *t* test, or one-way or two-way analysis of variance (ANOVA) with Tukey’s post hoc multiple comparisons test. All data are represented as mean ± SEM with significant differences denoted by numeric *p* values, or as *p < 0.05, **p < 0.01, ***p < 0.001, and ****p < 0.0001 unless otherwise stated. *p* < 0.05 was considered statistically significant.

### RESOURCE AVAILABILITY

#### Lead contact

Further information and requests for resources and materials should be directed to the lead contact, Ankur Singh (ankur.singh@gatech.edu).

#### Materials availability

This study did not generate new, unique materials.

#### Data and code availability

The main data supporting the findings of this study are available within the article and its supplemental information. This study did not generate any new code. Other raw data generated in this study are available from the lead contact upon reasonable request.

## Supplementary Material

1

SUPPLEMENTAL INFORMATION

Supplemental information can be found online at https://doi.org/10.1016/j.celbio.2026.100514.

## Figures and Tables

**Figure 1.**
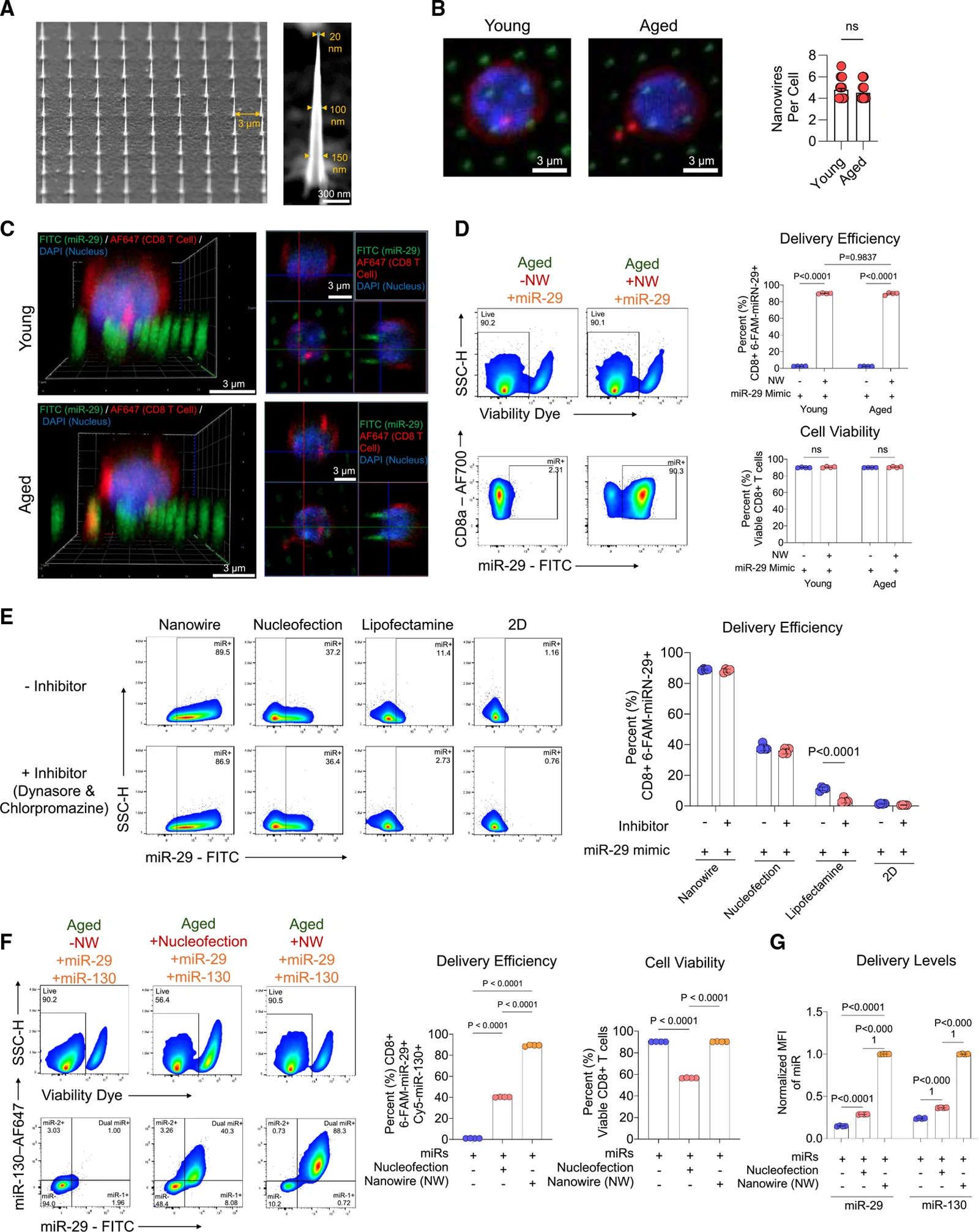
PEI-functionalized nanowires efficiently deliver miRNAs into young and aged murine CD8+ T cells (A) Scanning electron microscopy (SEM) images of a nanowire array (left) and a single nanowire (right). Scale bar represents 300 nm. (B) Quantification of the number of nanowires penetrated per cell from maximum intensity projection of confocal microscopy images of aged (18- to 24-month old) and young (3- to 6-month old) murine CD8+ T cells seeded on PEI-functionalized nanowires, which were loaded with 6-FAM-miR-29. Data are presented as mean ± SEM. Two-tailed, unpaired *t* test with Welch’s correction was performed (*n* = 45 cells). Scale bar represents 3 μm. (C) 3D projection and orthogonal projection of a confocal microscopy image of PEI-functionalized nanowires penetrating young and aged murine CD8+ T cells. Scale bar represents 3 μm. (D) Representative flow cytometry gating and bar graph of delivery efficiency and cell viability of 6-FAM-miR-29 in primary aged and young murine CD8+ T cells with and without PEI-functionalized nanowires. Data are presented as mean ± SEM. Two-way ANOVA with Tukey’s multiple comparison test (*n* = 4 nanowires). (E) Representative flow cytometry gating and bar graph of delivery efficiency of 6-FAM-miR-29 delivery in aged murine CD8+ T cells following nanowire-mediated delivery, nucleofection, Lipofectamine transfection, or 2D culture, with and without endocytic inhibition by dynasore and chlorpromazine. Data are presented as mean ± SEM. Two-way ANOVA with Tukey’s multiple comparison test (*n* = 4 nanowires). (F) Flow cytometry gating and bar graph of delivery efficiency (left) and cell viability (right) of dual miRNA (6-FAM-miR-29, cyanine5-miR-130) in primary aged murine CD8+ T cells with and without PEI-functionalized nanowires and with nucleofection. Data are presented as mean ± SEM. One-way ANOVA with Tukey’s multiple comparison test (*n* = 4 nanowires). (G) Delivery efficacy quantified as normalized median fluorescence intensity (MFI) of CD8+ T cells on 6-FAM-miR-29 and cyanine5-miR-130 delivery to primary aged murine CD8+ T cells with and without PEI-functionalized nanowires and with nucleofection. Data are presented as mean ± SEM. Two-way ANOVA with Tukey’s multiple comparison test (*n* = 4 nanowires).

**Figure 2.**
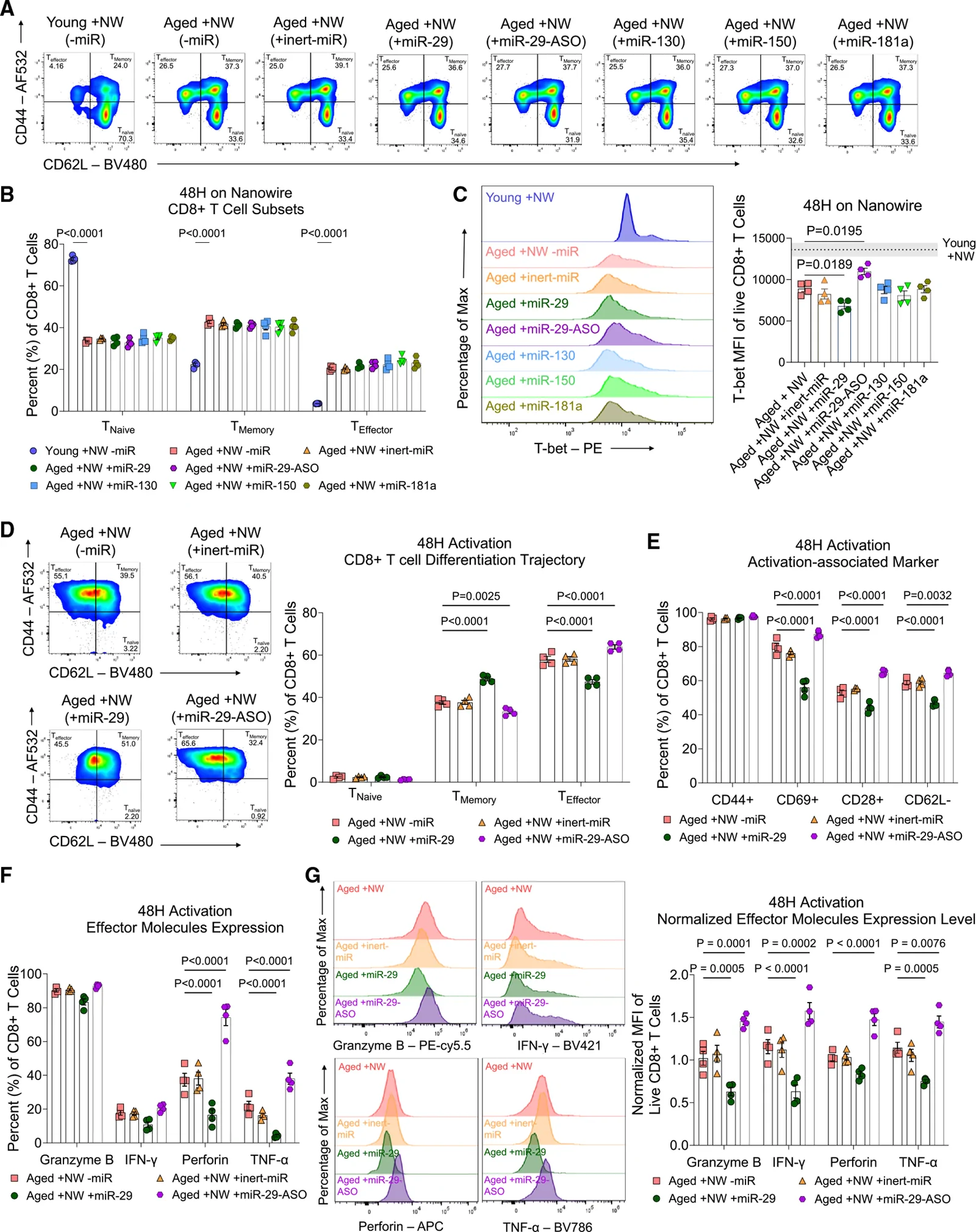
Specific miRNA delivery can alter differentiation trajectory, transcription factor expression, activation dynamics, and effector function of aged CD8+ T cells at resting and/or early activation stage (A and B) Flow cytometry gating of CD44 (AF532) versus CD62L (BV480) on viable murine CD8+ T cells to classify subsets—T_Naive_ (CD44− CD62L+), T_Memory_ (CD44+ CD62L+), T_Effector_ (CD44+ CD62L−)—across eight conditions: young +nanowire (NW) (−miR), aged +NW (−miR), aged +NW (+inert-miR), aged +NW (+miR-29), aged +NW (+miR-29-ASO), aged +NW (+miR-130), aged +NW (+miR-150), and aged +NW (+miR-181a) following nanowire incubation. Bar graph quantifying the percentage of each CD8+ T cells subset per condition. Data are presented as mean ± SEM. Two-way ANOVA with Tukey’s multiple comparison test (*n* = 4 nanowires). (C) Flow cytometry histograms (left) and bar graph (right) of the expression (MFI) of T-bet in total CD8+ T cells from eight miRNA treatment conditions following nanowire incubation. Data are presented as mean ± SEM. One-way ANOVA with Tukey’s multiple comparison test (*n* = 4 nanowires). (D) Flow cytometry gating of CD44 (AF532) versus CD62L (BV480) on viable CD8+ T cells after 48-h TCR activation to classify CD8+ T cell subsets across four miRNA treatment conditions and bar graph quantifying the percentage of each subset per condition. Data are presented as mean ± SEM. Two-way ANOVA with Tukey’s multiple comparison test (*n* = 4 nanowires). (E) Frequency of activation-associated markers CD44, CD69, CD28, and CD62L in nanowire-modulated aged murine CD8+ T cell groups treated with different miRNAs. Primary aged murine CD8+ T cells were incubated on nanowires for 48 h and TCR stimulation was done for the next 48 h. Data are presented as mean ± SEM. Two-way ANOVA with Tukey’s multiple comparison test (*n* = 4 nanowires). (F) Frequency of cells secreting effector cytokines (IFN-γ, TNF-α) and effector molecules (granzyme B, perforin) in nanowire-modulated murine aged CD8+ T cell groups treated with different miRNAs. Data are presented as mean ± SEM. Two-way ANOVA with Tukey’s multiple comparison test (*n* = 4 nanowires). (G) Flow cytometry histograms (left) and bar graph (right) of the normalized expression (MFI) of effector cytokines and effector molecules in nanowire-modulated murine CD8+ T cell groups treated with different miRNAs. Data are presented as mean ± SEM. Two-way ANOVA with Tukey’s multiple comparison test (*n* = 4 nanowires).

**Figure 3.**
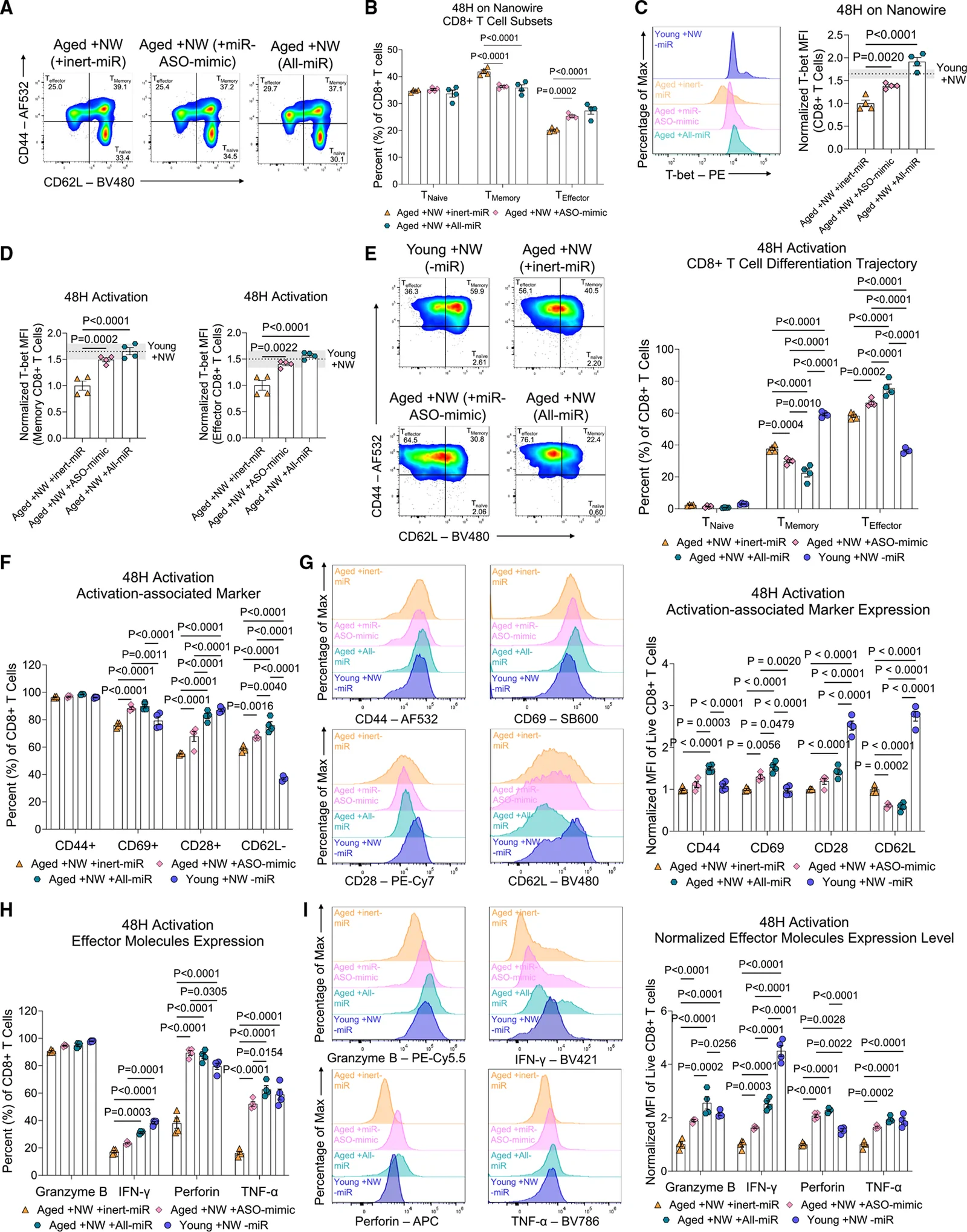
Nanowire-mediated multiplexed miRNA modulation primes aged CD8+ T cells toward a robust effector-dominant state (A and B) Flow cytometry gating of CD44 (AF532) versus CD62L (BV480) on viable CD8+ T cells to classify subsets—T_Naive_ (CD44− CD62L+), T_Memory_ (CD44+ CD62L+), T_Effector_ (CD44+ CD62L−)—across three conditions: aged +NW (+inert-miR), aged +NW (+miR-29-ASO +miR-130), and aged +NW (+miR-29-ASO +miR-130 +miR-150 +miR-181a). Bar graph quantifying the percentage of each subset per condition in (A). Data are presented as mean ± SEM. Two-way ANOVA with Tukey’s multiple comparison test (*n* = 4 nanowires). (C) Flow cytometry histograms of T-bet expression in total CD8+ T cells from nanowire-modulated groups and bar graph quantification of T-bet normalized MFI in CD8+ T cells. Data are presented as mean ± SEM. One-way ANOVA with Tukey’s multiple comparison test (*n* = 4 nanowires). (D) Quantification of normalized T-bet MFI in pan-CD8+ T cells and within the two subsets (T_Memory_, T_Effector_) across the three nanowire-modulated groups with the MFI of young CD8+ T cells as reference. Primary aged murine CD8+ T cells were incubated on nanowires for 48 h and TCR stimulation was done for the next 48 h. Data are presented as mean ± SEM. One-way ANOVA with Tukey’s multiple comparison test (*n* = 4 nanowires). (E) Flow cytometry gating of CD44 (AF532) versus CD62L (BV480) on viable murine CD8+ T cells after 48-h activation to classify CD8+ T cell subsets—T_Naive_ (CD44− CD62L+), T_Memory_ (CD44+ CD62L+), T_Effector_ (CD44+ CD62L−)—across the three miRNA-modulated conditions. Bar graph quantifying the percentage of each subset per condition. Data are presented as mean ± SEM. Two-way ANOVA with Tukey’s multiple comparison test (*n* = 4 nanowires). (F) Frequency of activation-associated markers: CD44+, CD69+, CD28+, and CD62L− cells in young and nanowire-mediated miRNA-modulated aged murine CD8+ T cells. Data are presented as mean ± SEM. Two-way ANOVA with Tukey’s multiple comparison test (*n* = 4 nanowires). (G) Flow cytometry histograms (left) and bar graph (right) of the expression (MFI) of activation-associated markers: CD44, CD69, CD28, and CD62L in young and different miRNA-modulated murine CD8+ T cells groups. Data are presented as mean ± SEM. Two-way ANOVA with Tukey’s multiple comparison test (*n* = 4 nanowires). (H) Frequency of effector cytokines (IFN-γ and TNF-α) and effector molecules (granzyme B, perforin) secreting cells in young and miRNA-modulated aged murine CD8+ T cells. Data are presented as mean ± SEM. Two-way ANOVA with Tukey’s multiple comparison test (*n* = 4 nanowires). (I) Flow cytometry histograms (left) and bar graph quantification (right) of the normalized secretion (MFI) of effector cytokines and effector molecules in young and different miRNA-modulated murine CD8+ T cells groups. Data are presented as mean ± SEM. Two-way ANOVA with Tukey’s multiple comparison test (*n* = 4 nanowires).

**Figure 4.**
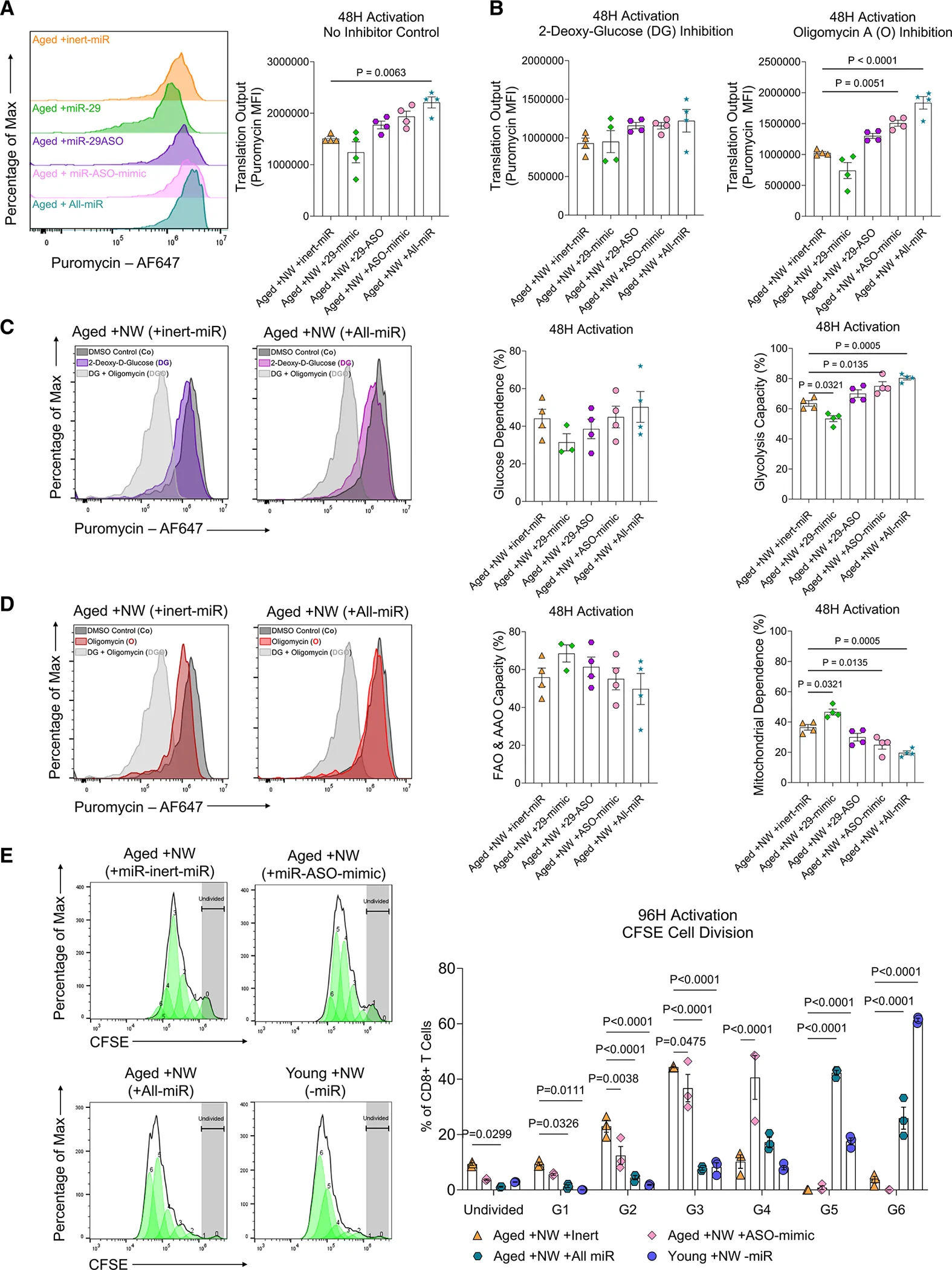
Nanowire-mediated multiplexed miRNA modulation enhances metabolic activity and cell proliferation in aged CD8+ T cells following activation (A) Flow cytometry histograms (left) and bar graph quantification (right) of SCENITH (single-cell energetic metabolism by profiling translation inhibition) total biogenic capacity (puromycin MFI) in aged murine CD8+ T cells across five miRNA treatment conditions: aged +NW (+inert-miR), aged +NW (+miR-29), aged +NW (+miR-29-ASO), aged +NW (+ASO-mimic), and aged +NW (+All-miR). Primary aged murine CD8+ T cells were incubated on nanowires for 48 h and TCR stimulation was done for the next 48 h before SCENITH analysis. Data are presented as mean ± SEM. One-way ANOVA with Tukey’s multiple comparison test. (B) Bar graph quantification of translation output (puromycin MFI) of different miRNA-modulated aged murine CD8+ T cell under the SCENITH inhibitor conditions 2-deoxy-D-glucose (DG), oligomycin (O). Data are presented as mean ± SEM. One-way ANOVA with Tukey’s multiple comparison test. (C) Representative flow cytometry histograms (left) and bar graph quantification (right) of glucose dependence,fatty acid oxidation (FAO) and amino acid oxidation (AAO) capacity in nanowire-modulated aged murine CD8+ T cell groups treated with different miRNA conditions. Data are presented as mean ± SEM. One-way ANOVA with Tukey’s multiple comparison test. (D) Representative flow cytometry histograms (left) and bar graph quantification (right) of mitochondrial dependence and glycolysis capacity in nanowire-modulated aged murine CD8+ T cell groups treated with different miRNA conditions. Data are presented as mean ± SEM. One-way ANOVA with Tukey’s multiple comparison test. (E) Proliferation analysis of nanowire-modulated aged murine CD8+ T cells assessed by carboxyfluorescein succinimidyl ester (CFSE) dilution. Left: representative histograms of CFSE fluorescence intensity showing sequential generations (G0–G6) of dividing CD8+ T cells under different nanowire delivery conditions. Right: quantification of the percentage of CD8+ T cells in each division generation. Primary aged murine CD8+ T cells were incubated on nanowires for 48 h, stained with CFSE, and TCR stimulation was done for the next 48 h. CD8+ T cells are harvested for CFSE analysis 48 h post withdrawal of TCR stimulation. Data are presented as mean ± SEM. Two-way ANOVA with Tukey’s multiple comparison test (*n* = 3 nanowires).

**Figure 5.**
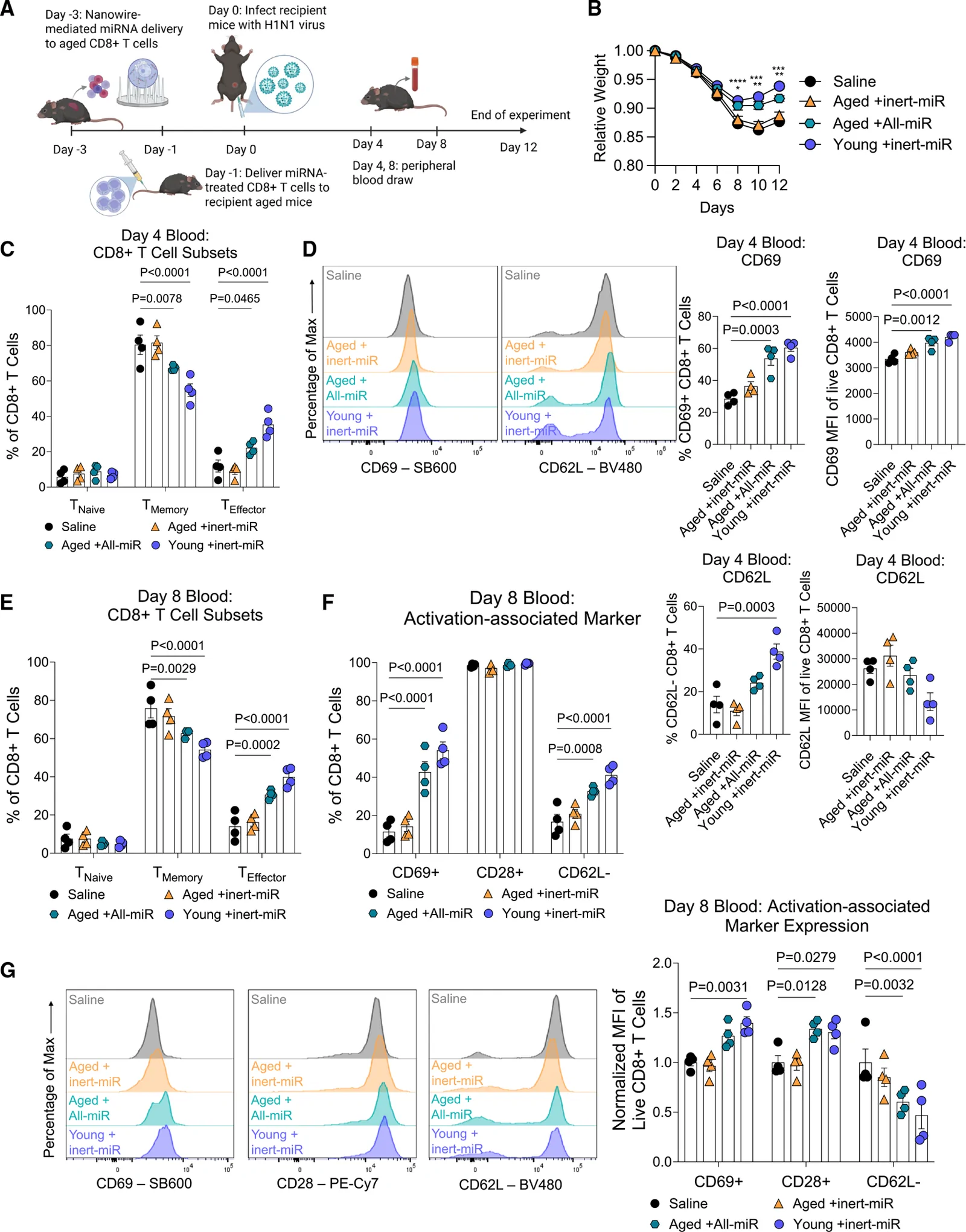
Adoptive transfer of multiplexed-miR-mediated CD8+ T cell enhances early effector priming and reduces weight loss during H1N1 infection in aged mice (A) Schematic and timeline of adoptive CD8+ T cell transfer and H1N1 virus infection on aged mice. (B) Normalized weight loss of aged mice under different treatment conditions: saline only, aged CD8+ T cell (+inert-miR), aged CD8+ T cell (+All-miR), young CD8+ T cell (+inert-miR), throughout primary H1N1 infection. Data are presented as mean ± SEM. One-way ANOVA with Tukey’s multiple comparison test at each time point (*n* = 4 mice). (C) Percentage of each CD8+ T cell subsets (T_Naive_ [CD44− CD62L+], T_Memory_ [CD44+ CD62L+], T_Effector_ [CD44+ CD62L−]) in peripheral blood of aged mice under different treatment conditions on day 4 after H1N1 primary infection. Data are presented as mean ± SEM. Two-way ANOVA with Tukey’s multiple comparison test (*n* = 4 mice). (D) Flow cytometry histograms and bar graph quantification of the frequency and expression (MFI) of activation-associated markers (CD69 and CD62L) in peripheral blood CD8+ T cells on day 4 after H1N1 primary infection. Data are presented as mean ± SEM. One-way ANOVA with Tukey’s multiple comparison test (*n* = 4 mice). (E) Quantification of the percentage of CD8+ T cell subset in peripheral blood CD8+ T cells of aged mice under different treatment conditions on day 8 after H1N1 primary infection. Data are presented as mean ± SEM. Two-way ANOVA with Tukey’s multiple comparison test (*n* = 4 mice). (F) Frequency of activation-associated markers in peripheral blood CD8+ T cells of aged mice under different treatment conditions on day 8 after H1N1 primary infection. Data are presented as mean ± SEM. One-way ANOVA with Tukey’s multiple comparison test (*n* = 4 mice). (G) Flow cytometry histograms (left) and bar graph (right) of the normalized expression (MFI) of activation-associated markers in peripheral blood CD8+ T cells of aged mice under different treatment conditions on day 8 after H1N1 primary infection. Data are presented as mean ± SEM. One-way ANOVA with Tukey’s multiple comparison test (*n* = 4 mice).

**Figure 6.**
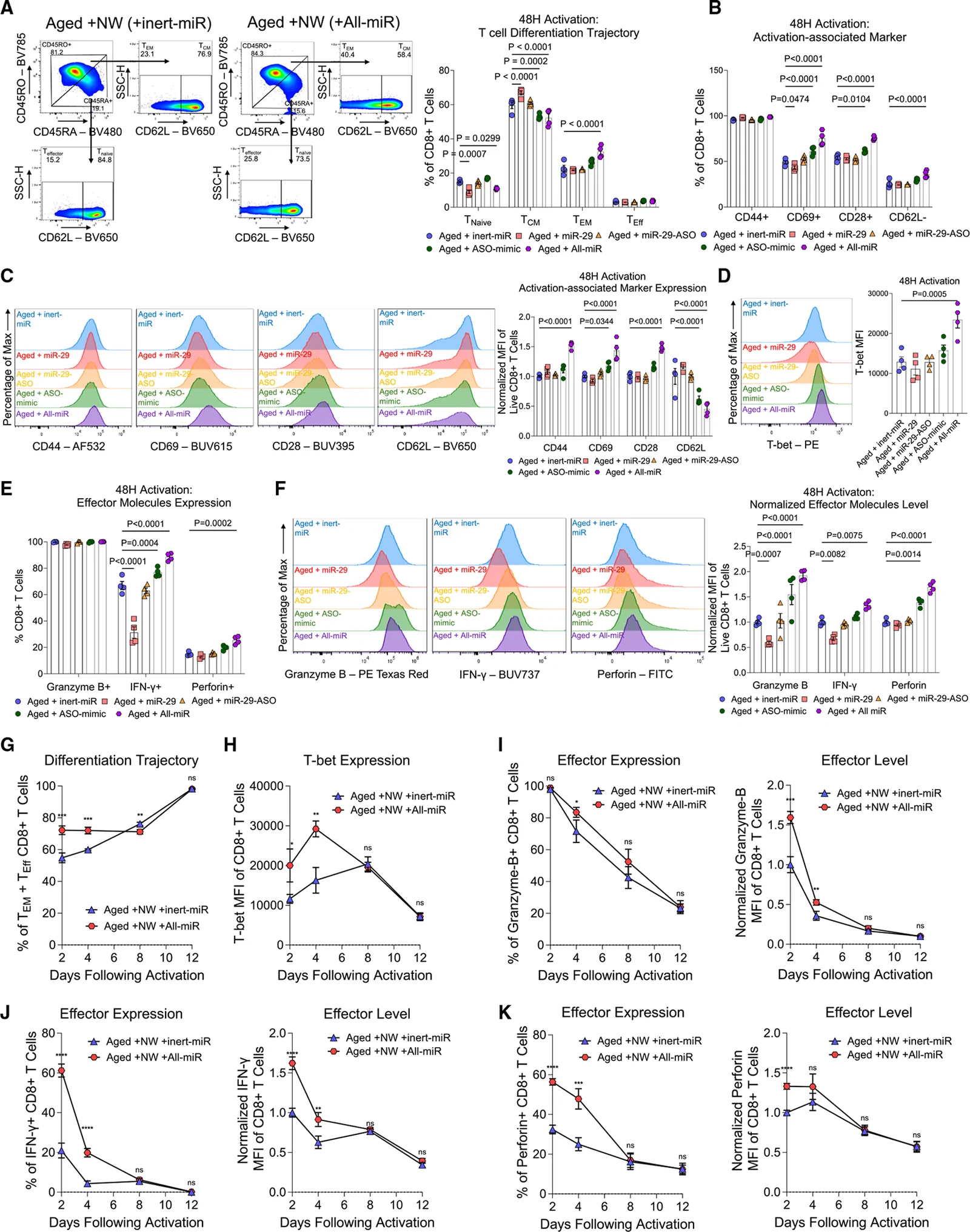
Nanowire-mediated multiplexed miRNA modulation sustains effector priming of aged human CD8+ T cells across early activation and expansion stages (A) Representative flow cytometry gating of CD45RA (BV480) versus CD45RO (BV785) and CD62L (BV650) on viable aged human CD8+ T cells after 48 h activation to classify CD8+ T cell subsets (T_Naive_ [CD45RA+ CD62L+], T_Central Memory_ [CD45RO+ CD62L+], T_Effector Memory_ [CD45RO+ CD62L−], and T_Effector_ [CD45RA+ CD62L−]) in aged human CD8+ T cells across five miRNA treatment conditions: aged +NW (+inert-miR), aged +NW (+miR-29), aged +NW (+miR-29-ASO), aged +NW (+ASO-mimic), and aged +NW (+All-miR). Bar graph quantification of the percentage of each CD8+ T cell subset per condition. Primary aged human CD8+ T cells were incubated on nanowires for 48 h and TCR stimulation was done for the next 48 h. Data are presented as mean ± SEM. Two-way ANOVA with Tukey’s multiple comparison test (*n* = 4 nanowires). (B) Bar graph quantification on frequency of activation-associated markers CD44+, CD69+, CD28+, and CD62L− cells from different miRNA-modulated aged human CD8+ T cells conditions. Data are presented as mean ± SEM. Two-way ANOVA with Tukey’s multiple comparison test (*n* = 4 nanowires). (C) Flow cytometry histograms (left) and bar graph (right) quantification of the normalized expression (MFI) of activation-associated markers CD44, CD69, CD28, and CD62L from different miRNA-modulated aged human CD8+ T cells conditions. Data are presented as mean ± SEM. Two-way ANOVA with Tukey’s multiple comparison test (*n* = 4 nanowires). (D) Flow cytometry histograms of T-bet expression in human CD8+ T cells from different miRNA-modulated groups and bar graph quantification of normalized T-bet expression (MFI) in human CD8+ T cells. Data are presented as mean ± SEM. One-way ANOVA with Tukey’s multiple comparison test (*n* = 4 nanowires). (E) Frequency of effector cytokines and effector molecules (granzyme B, IFN-γ, and perforin) expressing cells from different miRNA-modulated aged human CD8+ T cells conditions. Data are presented as mean ± SEM. Two-way ANOVA with Tukey’s multiple comparison test (*n* = 4 nanowires). (F) Flow cytometry histograms (left) and bar graph quantification (right) of the normalized expression (MFI) of effector cytokines and effector molecules (granzyme B, IFN-γ, and perforin) from different miRNA-modulated aged human CD8+ T cells conditions. Data are presented as mean ± SEM. Two-way ANOVA with Tukey’s multiple comparison test (*n* = 4 nanowires). (G) Longitudinal quantification of percentage of effector-like cells (T_Effector Memory_ + T_Effector_) in aged human CD8+ T cells treated with inert-miR or All-miR at different time points following activation. Primary aged human CD8+ T cells were incubated on nanowires for 48 h and TCR stimulation was done for the next 48 h. Dynabeads were withdrawn after 48 h activation, and cells were maintained in culture and analyzed on days 2, 4, 8, and 12 following activations. Data are presented as mean ± SEM. Two-tailed, unpaired *t* test with Welch’s correction was performed at each time point (*n* = 4 nanowires), *p < 0.05, **p < 0.01, ***p < 0.001, and ****p < 0.0001. (H) Longitudinal quantification of T-bet expression (MFI) in aged human CD8+ T cells treated with (1) inert-miR or (2) All-miR at different time point following activation. Data are presented as mean ± SEM. Two-tailed, unpaired *t* test with Welch’s correction was performed at each time point (*n* = 4 nanowires), *p < 0.05, **p < 0.01, ***p < 0.001, and ****p < 0.0001. (I–K) Longitudinal quantification of effector molecules expressing cells and normalized expression (MFI) of effector molecules (granzyme B, I; IFN-γ, J; perforin, K) in aged human CD8+ T cells treated with inert-miR or All-miR at different time point following activation. Data are presented as mean ± SEM. Two-tailed, unpaired *t* test with Welch’s correction was performed at each time point (*n* = 4 nanowires), *p < 0.05, **p < 0.01, ***p < 0.001, and ****p < 0.0001.

**Figure 7.**
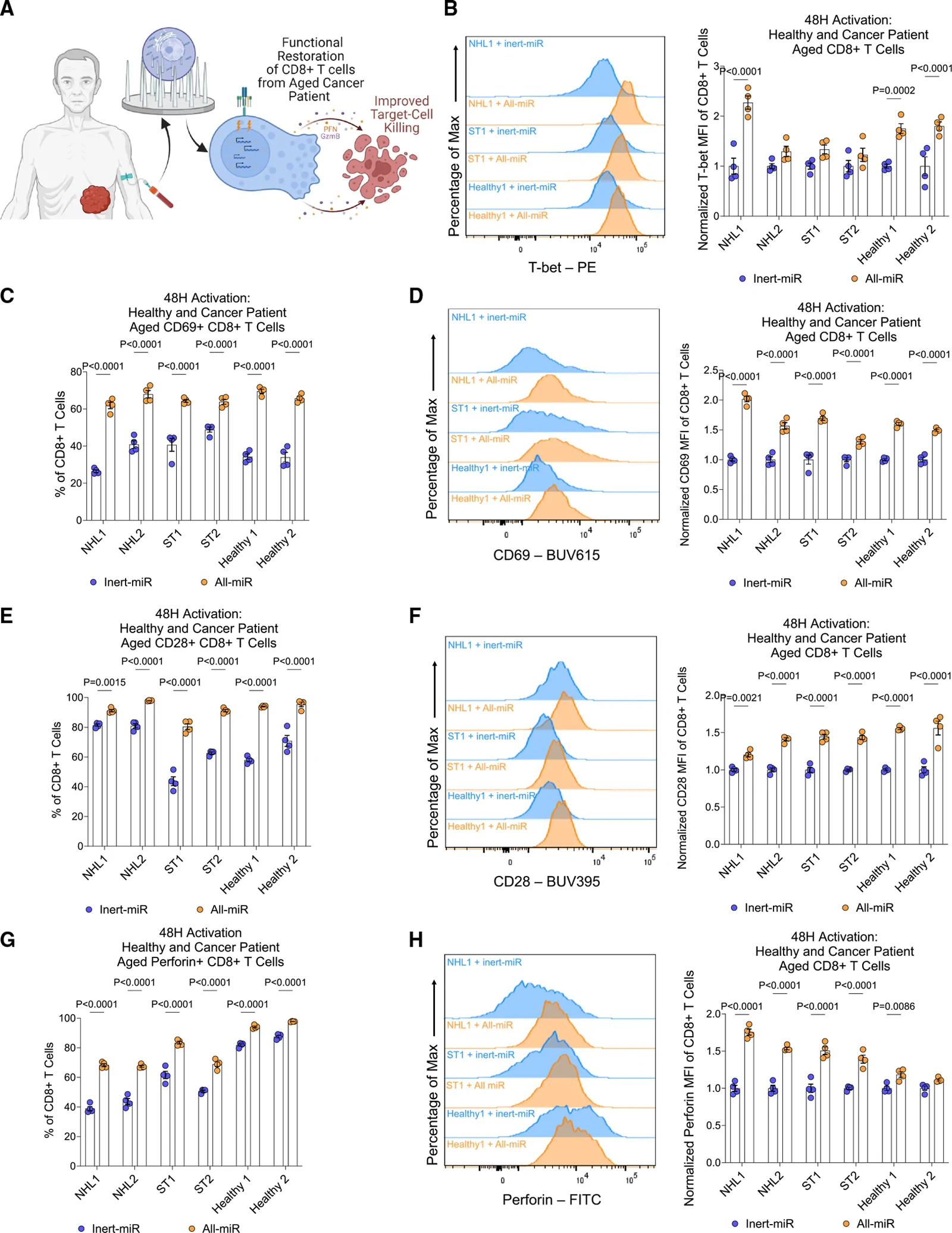
Combinational miRNA programming enhances activation dynamics of aged CD8+ T cells from healthy individuals and cancer patients (A) Schematics for nanowire-mediated multiplexed delivery of miRNA to aged CD8+ T cells from healthy individuals and cancer patients for T cell enhancement and improved target-cell killing capabilities of T cells. (B) Flow cytometry histograms of T-bet expression in nanowire-modulated aged human CD8+ T cells from healthy individuals, donors with current or prior cancer treated with inert-miR or multiplexed miRNAs, and bar graph quantification of normalized T-bet expression (MFI) in CD8+ T cells. Primary aged human CD8+ T cells are from non-Hodgkin lymphoma (NHL) survivors, solid tumor cancer patients who had not received systemic cancer-directed therapy (ST), and healthy aged patients (healthy). Cells were incubated on nanowires for 48 h and TCR stimulation was done for the next 48 h. Data are presented as mean ± SEM. Two-way ANOVA with Tukey’s multiple comparison test (*n* = 4 nanowires). (C) Quantification on frequency of activation-associated marker CD69+ cells in nanowire-modulated human CD8+ T cells from healthy individuals and donors with current or prior cancer treated with inert-miR or multiplexed miRNAs. Data are presented as mean ± SEM. Two-way ANOVA with Tukey’s multiple comparison test (*n* = 4 nanowires). (D) Flow cytometry histograms (left) and bar graph (right) quantification of the normalized expression (MFI) of activation-associated marker CD69 in nanowire-modulated human CD8+ T cells from healthy individuals and donors with current or prior cancer treated with inert-miR or multiplexed miRNAs. Data are presented as mean ± SEM. Two-way ANOVA with Tukey’s multiple comparison test (*n* = 4 nanowires). (E) Quantification on frequency of activation-associated marker CD28+ cells in nanowire-modulated human CD8+ T cells from healthy individuals and donors with current or prior cancer treated with inert-miR or multiplexed miRNAs. Data are presented as mean ± SEM. Two-way ANOVA with Tukey’s multiple comparison test (*n* = 4 nanowires). (F) Flow cytometry histograms (left) and bar graph (right) quantification of the normalized expression (MFI) of activation-associated marker CD28 in nanowire-modulated human CD8+ T cells from healthy individuals and donors with current or prior cancer treated with inert-miR or multiplexed miRNAs. Data are presented as mean ± SEM. Two-way ANOVA with Tukey’s multiple comparison test (*n* = 4 nanowires). (G) Quantification on frequency of effector molecules perforin+ secreting cells in nanowire-modulated human CD8+ T cells from healthy individuals and donors with current or prior cancer treated with inert-miR or multiplexed miRNAs. Data are presented as mean ± SEM. Two-way ANOVA with Tukey’s multiple comparison test (*n* = 4 nanowires). (H) Flow cytometry histograms (left) and bar graph (right) quantification of the normalized expression (MFI) of effector molecules perforin in nanowire-modulated human CD8+ T cells from healthy individuals and donors with current or prior cancer treated with inert-miR or multiplexed miRNAs. Data are presented as mean ± SEM. Two-way ANOVA with Tukey’s multiple comparison test (*n* = 4 nanowires).

## THE BIGGER PICTURE

As individuals age, the immune system undergoes a transformation rather than merely experiencing decline; it is actively reshaped. CD8+ T cells, frontline defenders against infection and cancer, are particularly affected. In youth, these cells display diversity, adaptability, and strong responsiveness. With age, however, they become less abundant, less flexible, and more prone to dysfunction, weakening responses to new pathogens, tumors, and vaccines. Part of this decline arises from dysregulation of microRNAs (miRNAs), small regulatory molecules that fine-tune gene expression. In healthy T cells, miRNAs coordinate activation, growth, metabolism, differentiation, and cytotoxic function. In aged cells, this balance is disrupted, contributing to impaired immunity. Yet efficient delivery of miRNAs into primary human T cells, especially from older donors, remains a significant technical challenge.

This study addresses this barrier using a biomaterial-based silicon nanowire platform. These engineered nanoscale interfaces interact with cell membranes and physically deliver miRNAs directly into aged CD8+ T cells, avoiding viral vectors. This enables transient modulation of intracellular regulatory networks while preserving viability. Single miRNA delivery induces targeted shifts in early activation, whereas multiplexed delivery produces broader coordinated effects, including enhanced activation, metabolism, proliferation, and cytotoxic capacity. In murine models, treated T cells show improved early responses after antigen exposure. In human CD8+ T cells from older donors, including those with current or prior cancer, this approach enhances activation and cytotoxic potential while supporting an effector-ready state during expansion. Conceptually, this work shifts emphasis from cellular replacement or non-specific stimulation toward precise tuning of internal regulatory circuitry. More broadly, it illustrates how engineered biomaterials can deliver regulatory cues and temporarily reshape immune cell function in a controlled manner. These findings provide a foundation for translational strategies to rejuvenate aging immunity and improve vaccine efficacy and cancer immunotherapy outcomes in vulnerable elderly populations in future settings.

## Highlights

- Nanowire enables efficient delivery of miRNAs into resting aged CD8+ T cells
- Multiplexed miRNA delivery enhances activation and effector responses in T cells
- *Ex vivo* miRNA-programmed aged T cells exhibit enhanced effector priming *in vivo*
- Multiplexed miRNA enhances T cell function of healthy and cancer aged donors
