# Supplementary material for: miRNA delivery via nanowires restores functional responses in aged T cells
Source: Cell Biomater. Author manuscript; Available in PMC 2026 Nov 4. (PMC13730167; doi:10.1016/j.celbio.2026.100514)
Supplement: Supplementary file 1 [file NIHMS2210992-supplement-1.pdf]

CELBIO, Volume *2*

Supplemental information

miRNA delivery via nanowires restores functional responses in aged T cells

Zhonghao Dai, Shaylyn Grier, Zhe Zhong, Ameya Dravid, Joscelyn Mejías, Jean L. Koff, Andrés J. García, and Ankur Singh

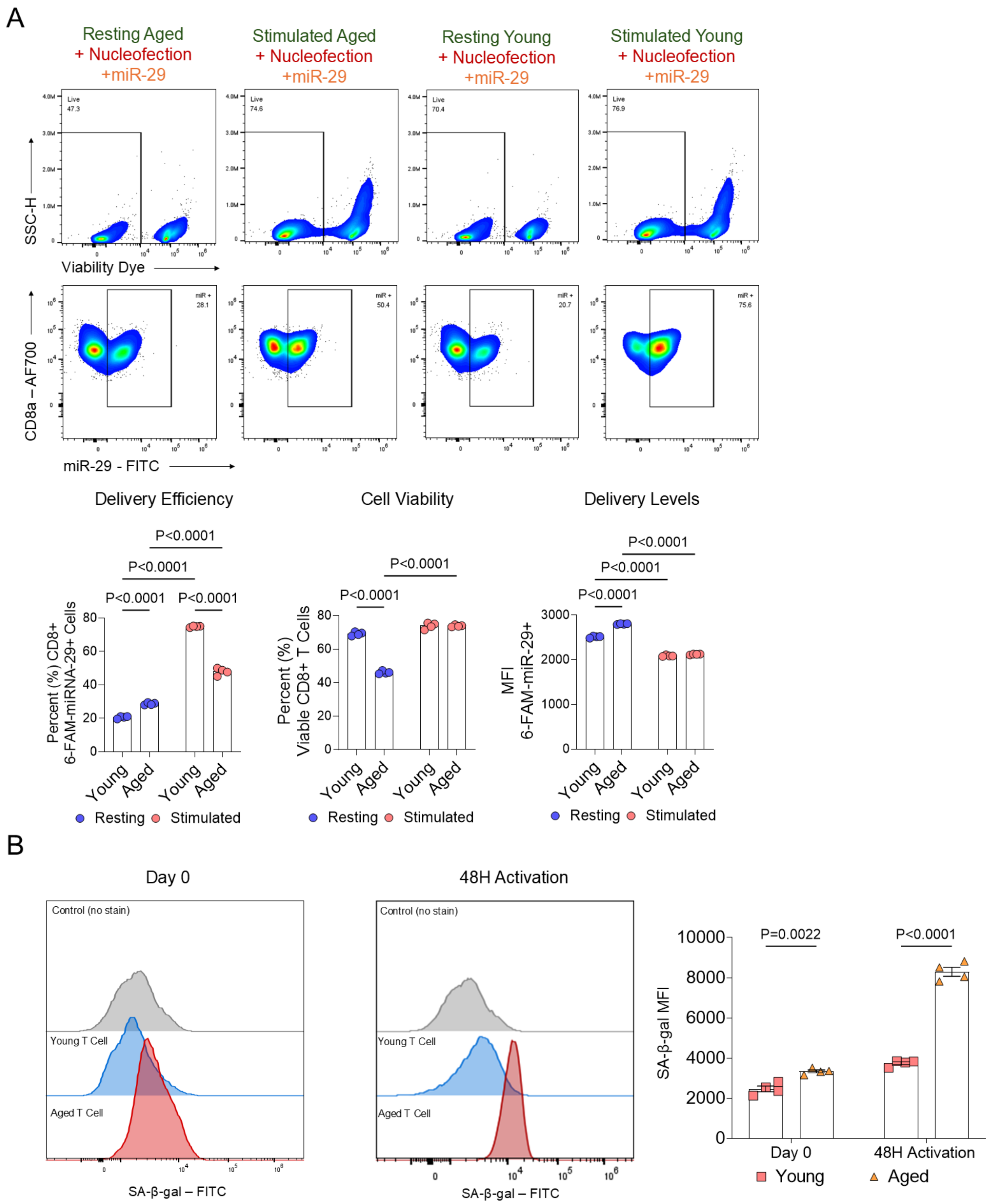

Figure S1. Nucleofection benchmarking and SA-β-gal staining establish the delivery limitations and aged phenotype of murine CD8+ T cells.

A, Flow cytometry gating, and bar graph of delivery efficiency, cell viability, and delivery level of 6-FAM-miR-29 in primary aged and young murine CD8+ T cells via nucleofection with and without prior TCR stimulation. Data are presented as mean ± s.e.m. Two-way ANOVA with Tukey's multiple comparison test (n = 4 nanowires). **B**, Representative flow cytometry histograms of Senescence-associated beta-galactosidase (SA-β-gal) staining in unstained control, young murine CD8+ T cells, and aged murine CD8+ T cells at Day 0 and after 48 h activation. And Bar graph quantification of SA-β-gal level (MFI) in young and aged murine CD8+ T cells at Day 0 and after 48 h activation. Data are presented as mean ± s.e.m. Two-way ANOVA with Tukey's multiple comparison test (n = 4 wells).

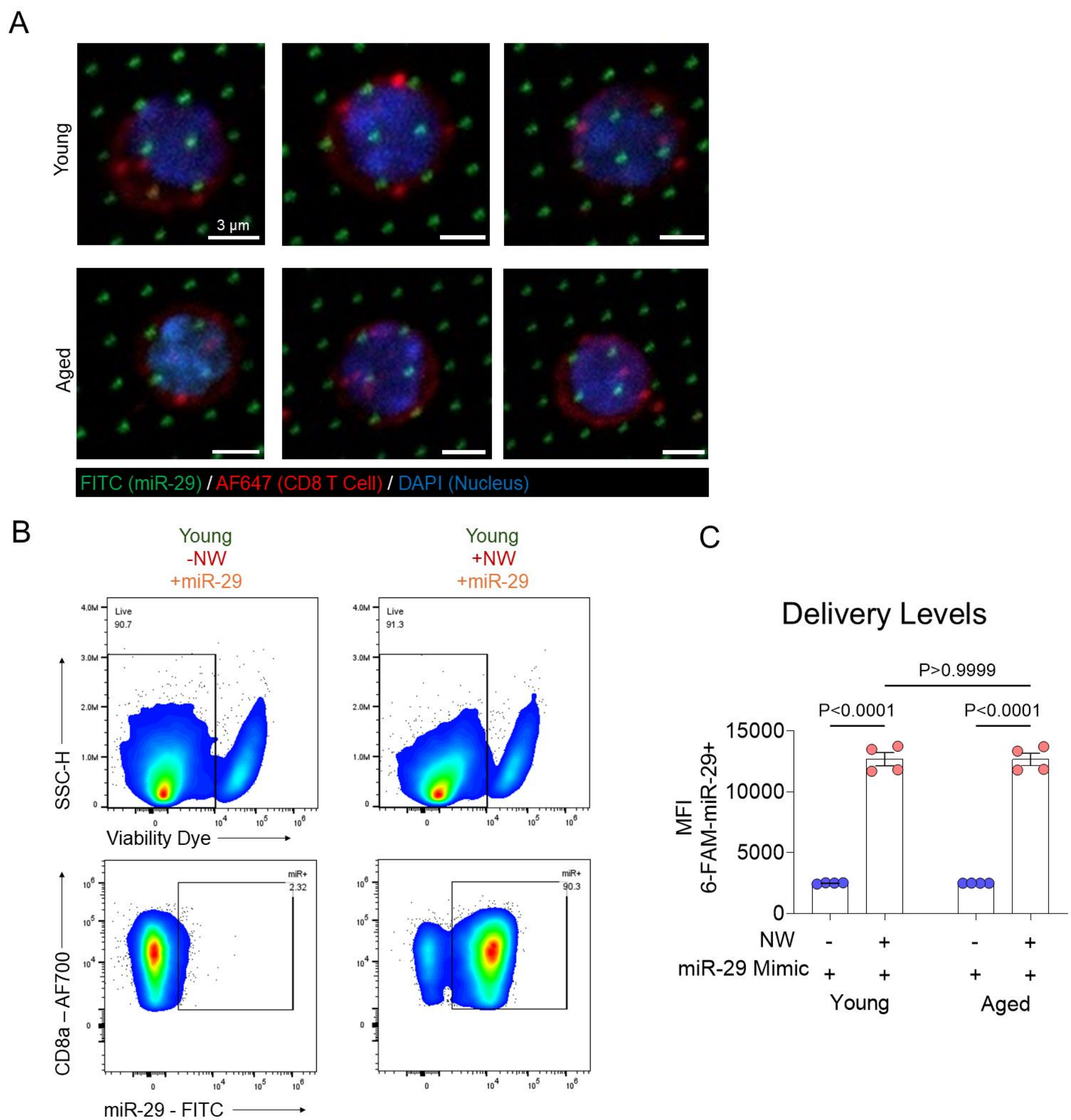

Figure S2. PEI-functionalized nanowires support efficient 6-FAM–miR-29 delivery into young and aged murine CD8+ T cells.

A, Additional maximum intensity projection of confocal microscopy images of aged and young murine CD8+ T cells seeded on PEI-functionalized nanowires were loaded with 6-FAM-miR-29. **B**, Representative flow cytometry gating of 6-FAM-miR-29 in primary young murine CD8+ T cells with and without PEI-functionalized nanowires. **C**, Delivery efficacy quantified as 6-FAM MFI of CD8+ T cells on 6-FAM-miR-29 delivery to primary aged and young murine CD8+ T cells with and without PEI-functionalized nanowires. Data are presented as mean ± s.e.m. Two-way ANOVA with Tukey's multiple comparison test (n = 4 nanowires).

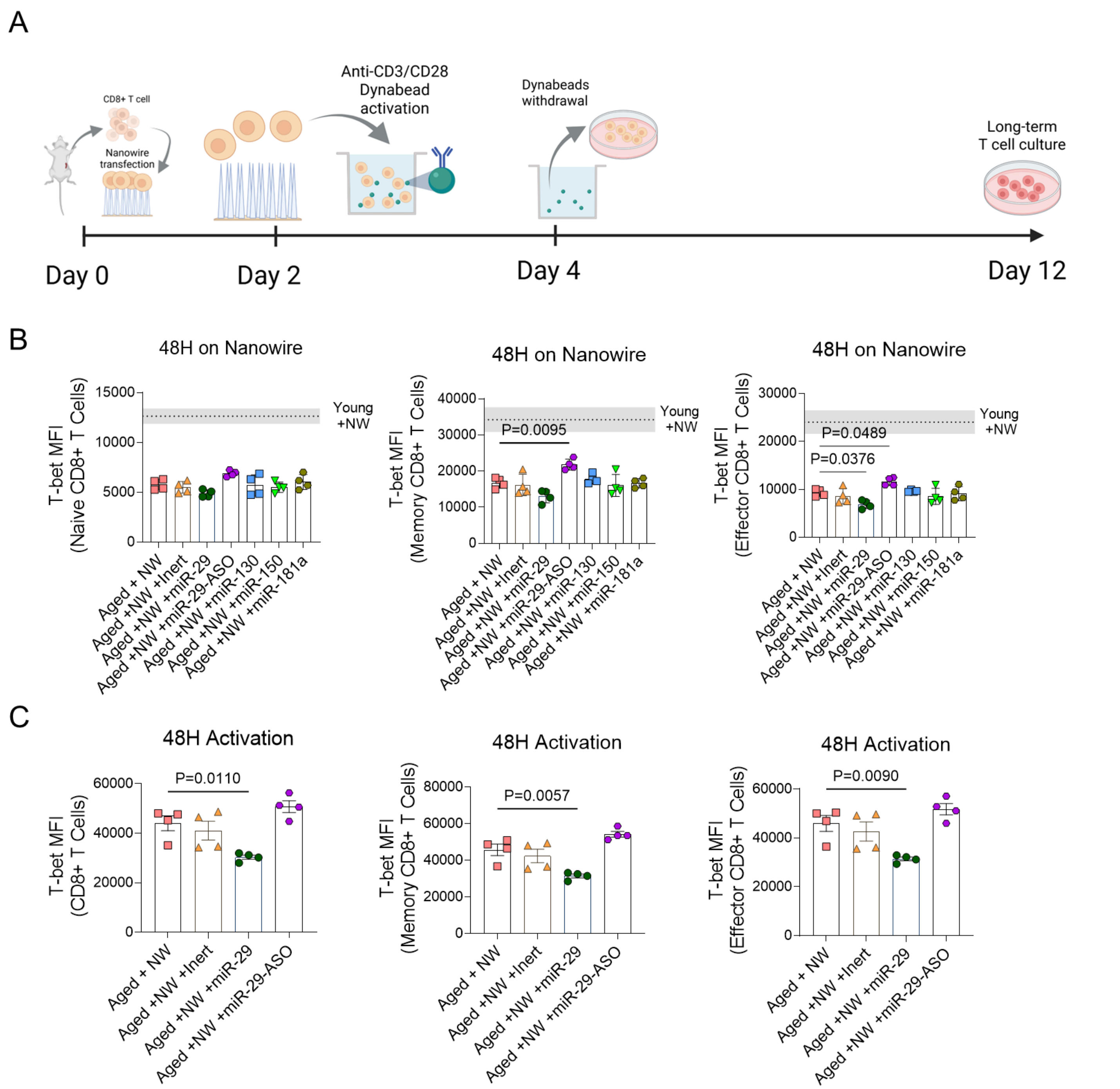

Figure S3. Nanowire-mediated miRNA delivery modulates T-bet expression in aged murine CD8+ T-cell subsets before and after activation.

A, Schematic and experimental timeline illustrating the workflow for nanowire-mediated miRNA transfection and subsequent T-cell activation. Primary CD8+ T cells were seeded onto polyethyleneimine (PEI)-functionalized silicon nanowires for miRNA delivery (Day 0–2), followed by activation using anti-CD3/CD28 Dynabeads (Day 2–4). Dynabeads were withdrawn on Day 4, and cells were maintained in long-term culture until Day 12 for downstream functional analyses. **B**, Quantification of T-bet MFI within the three subsets (T_{Naive}, T_{Memory}, $T_{Effector}$) of CD8+ T cells from eight miRNA treatment conditions following nanowire incubation. Data are presented as mean ± s.e.m. One-way ANOVA with Tukey's multiple comparison test (n = 4 nanowires). **C**, Quantification of T-bet MFI after 48-hour activation in pan-CD8+ T cells and within the two subsets (T_{Memory}, and $T_{Effector}$) across aged CD8+ T cell groups treated with different miRNAs. Data are presented as mean ± s.e.m. One-way ANOVA with Tukey's multiple comparison test (n = 4 nanowires).

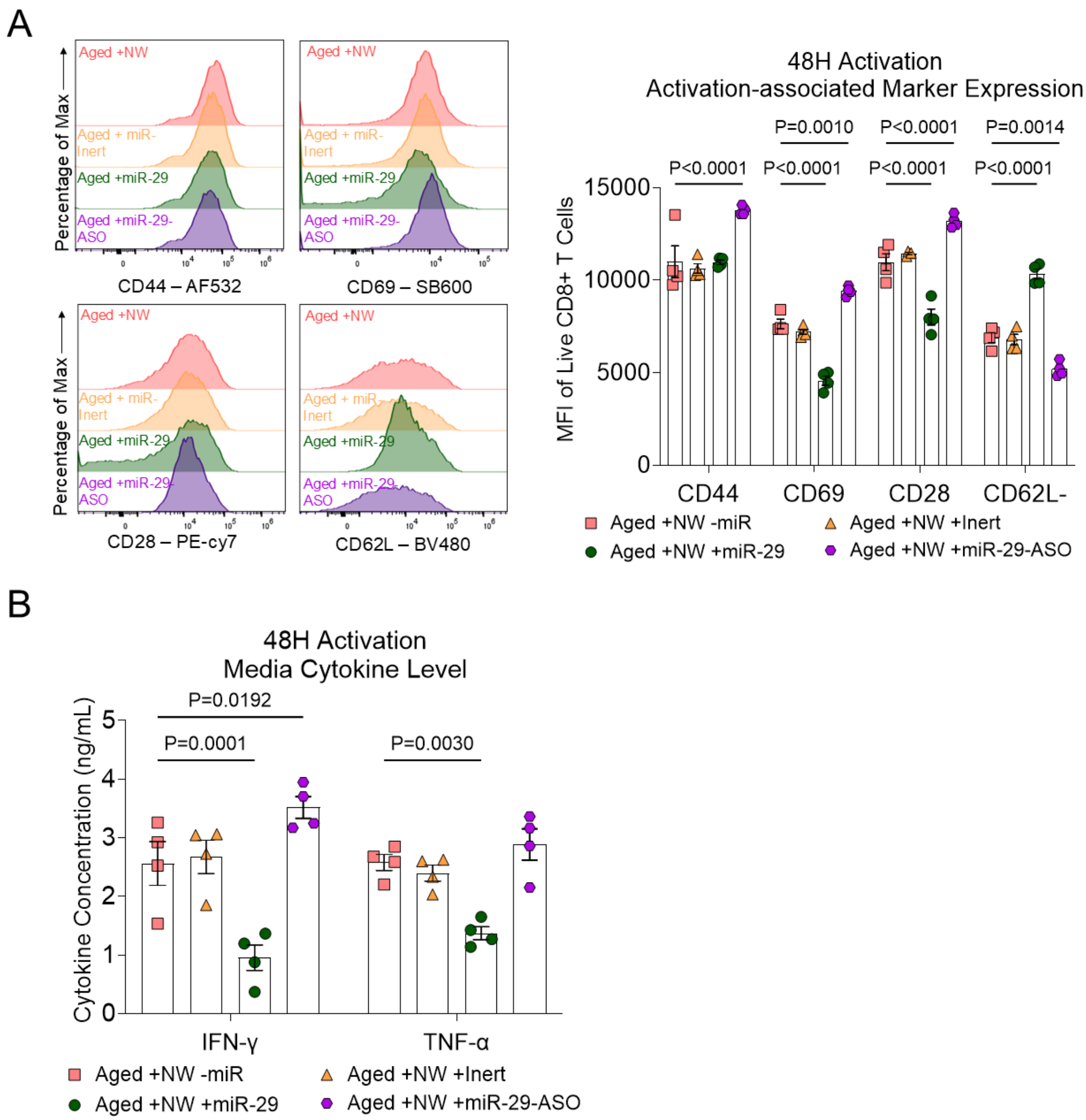

Figure S4. Single miR-29-axis modulation differentially regulates activation-marker expression and secreted cytokine output in aged murine CD8+ T cells.

A, Flow cytometry histograms (Left) and bar graph (Right) of the expression (MFI) of activation-associated markers CD44, CD69, CD28, and CD62L in nanowire-modulated murine CD8+ T cell groups treated with different miRNAs. Data are presented as mean ± s.e.m. Two-way ANOVA with Tukey's multiple comparison test (n = 4 nanowires). **B**, Quantification of secreted effector cytokines IFN-γ and TNF-α in culture supernatants of nanowire-modulated aged murine CD8+ T cell groups treated with different miRNAs. Primary aged CD8+ T cells were incubated on nanowires for 48 h, followed by 48 h of TCR stimulation, and cytokine concentrations were measured in the media. Data are presented as mean ± s.e.m. Two-way ANOVA with Tukey's multiple comparison test (n = 4 nanowires).

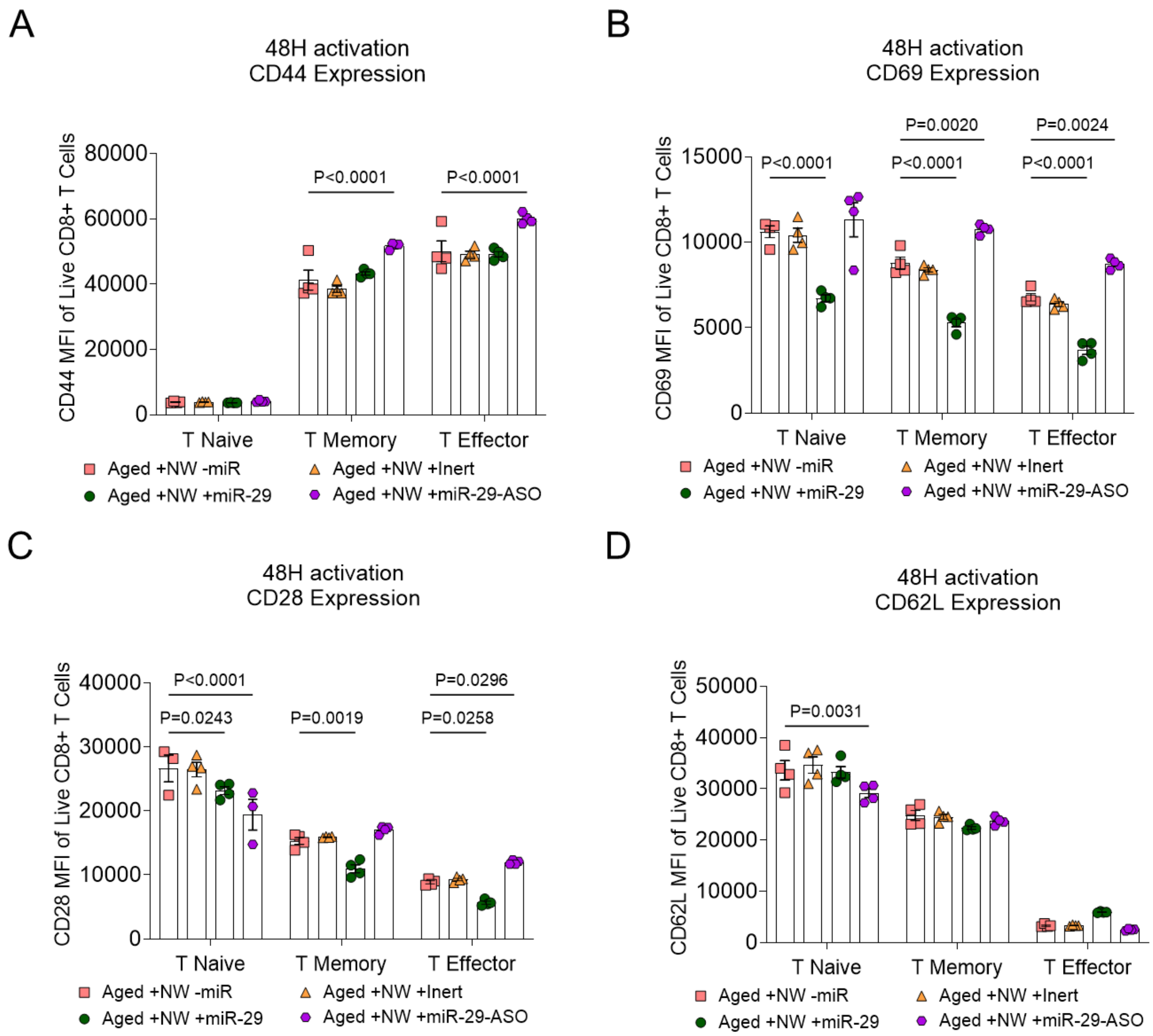

Figure S5. Single miR-29-axis modulation alters activation-marker expression across aged murine CD8+ T-cell differentiation subsets.

A-D, Bar graph quantification of median fluorescence intensity (MFI) of activation associated marker CD44 (**A**), CD69 (**B**), CD28 (**C**), and CD62L (**D**) after 48-hour activation within the three CD8+ T cell subsets (T_{Naive}, T_{Memory}, $T_{Effector}$) across aged murine CD8+ T cell groups treated with different miRNAs. Data are presented as mean ± s.e.m. One-way ANOVA with Tukey's multiple comparison test (n = 4 nanowires).

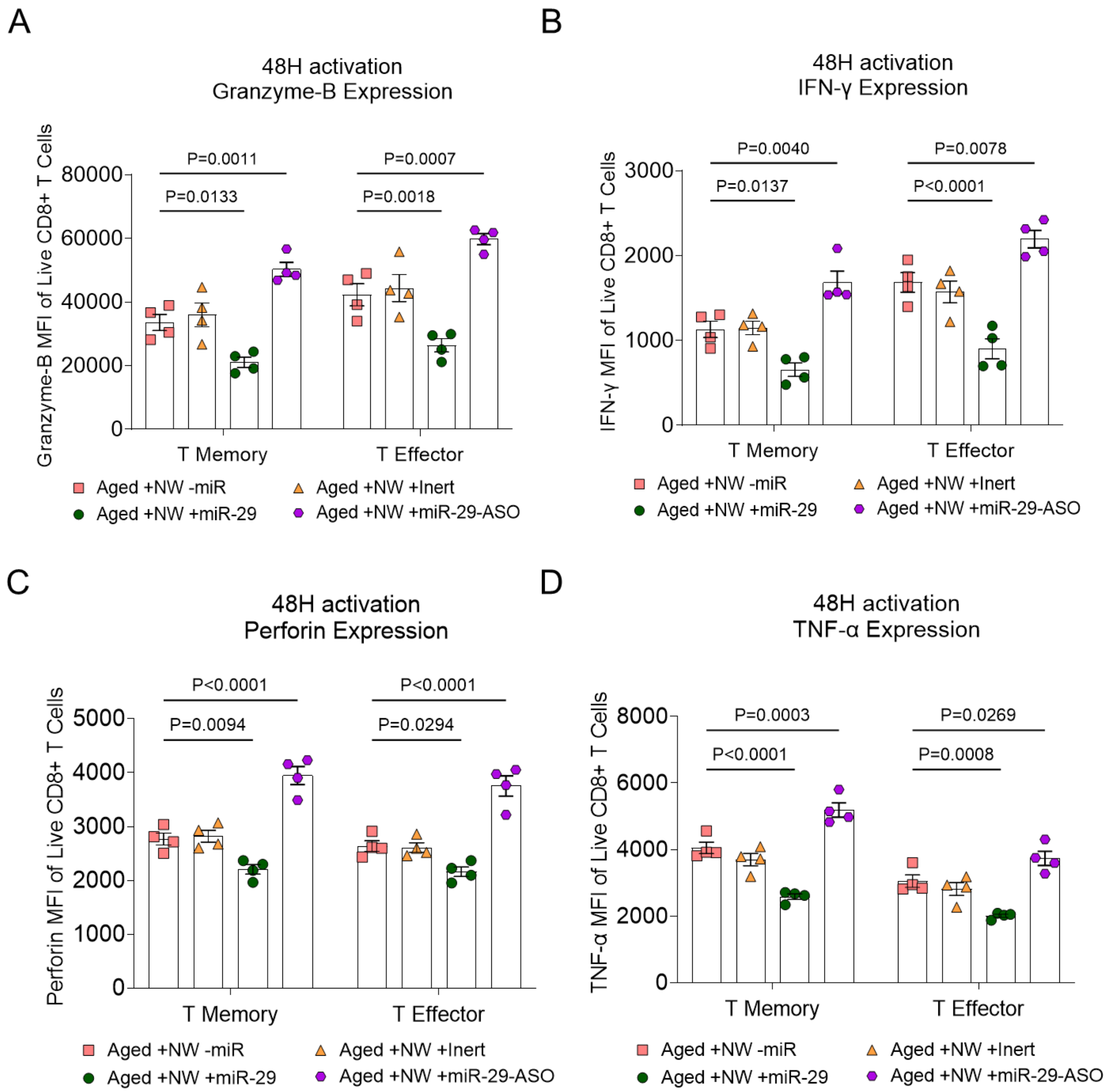

Figure S6. Single miR-29-axis modulation reshapes effector-molecule expression across aged murine memory and effector CD8+ T-cell subsets.

A-D, Bar graph quantification of median fluorescence intensity (MFI) for the expression of effector molecules Granzyme B (**A**), IFN-γ (**B**), Perforin (**C**), and TNF-α (**D**) after 48-hour activation within the two CD8+ T cell subsets (T_{Memory}, $T_{Effector}$) across aged murine CD8+ T cell groups treated with different miRNAs. Data are presented as mean ± s.e.m. One-way ANOVA with Tukey's multiple comparison test (n = 4 nanowires).

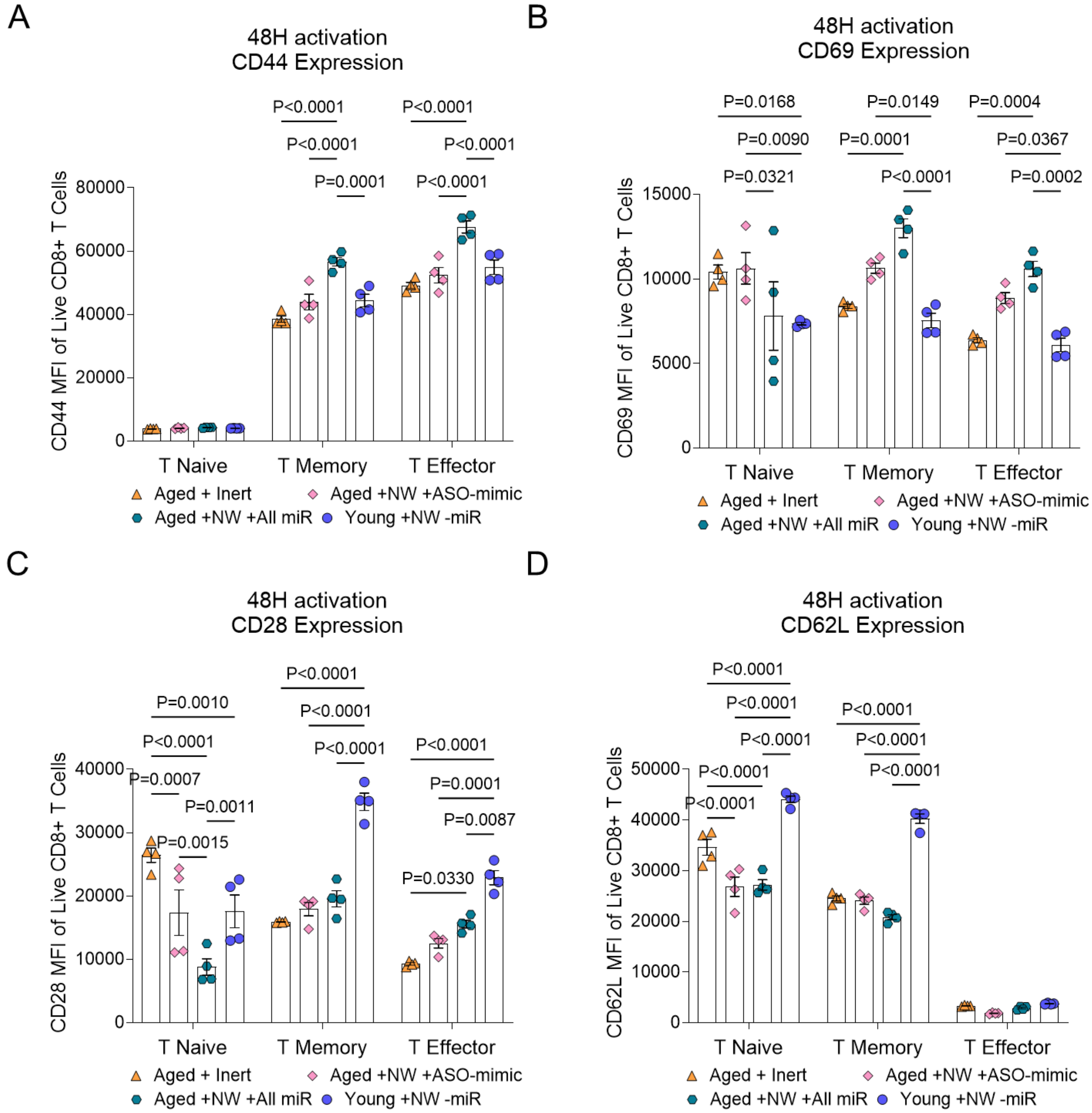

Figure S7. Multiplexed miRNA delivery broadly enhances activation-associated marker expression across nanowire-programmed aged murine CD8+ T-cell subsets.

A-D, Bar graph quantification of median fluorescence intensity (MFI) of activation associated marker CD44 (**A**), CD69 (**B**), CD28 (**C**), and CD62L (**D**) after 48-hour activation within the three CD8+ T cell subsets (T_{Naive}, T_{Memory}, $T_{Effector}$) across young and in young and nanowire-modulated aged murine CD8+ T cell groups treated with different miRNAs. Data are presented as mean ± s.e.m. One-way ANOVA with Tukey's multiple comparison test (n = 4 nanowires).

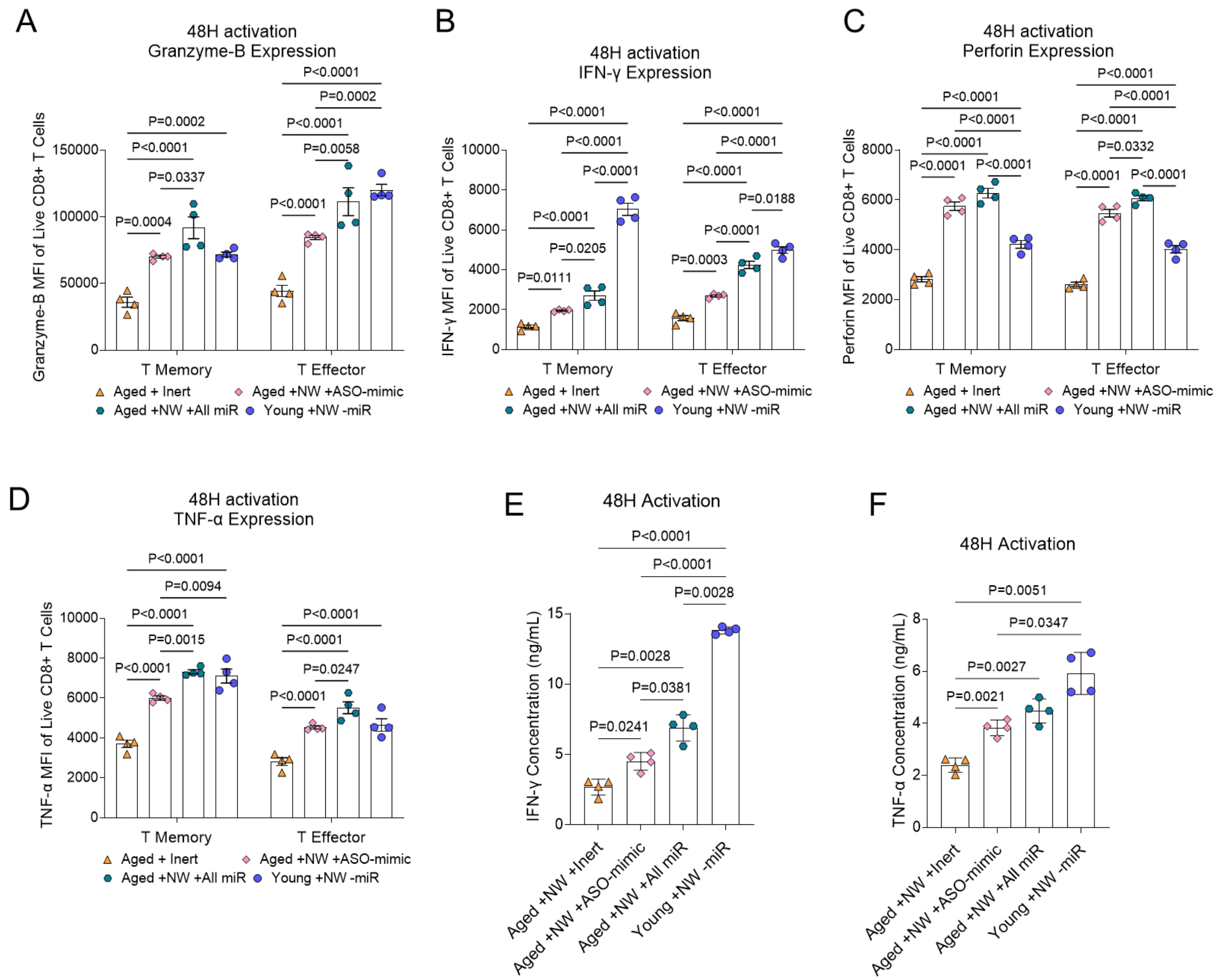

Figure S8. Multiplexed miRNA delivery increases effector-molecule expression and cytokine secretion in nanowire-programmed aged murine CD8+ T cells.

A-D, Bar graph quantification of median fluorescence intensity (MFI) for the expression of effector molecules Granzyme B (**A**), IFN-γ (**B**), Perforin (**C**), and TNF-α (**D**) after 48-hour activation within the two CD8+ T cell subsets (T_{Memory}, $T_{Effector}$) across young and in young and nanowire-modulated aged murine CD8+ T cell groups treated with different miRNAs. Data are presented as mean ± s.e.m. One-way ANOVA with Tukey's multiple comparison test (n = 4 nanowires). **E-F**, Bar graph quantification of secreted effector cytokines IFN-γ (**E**) and TNF-α (**F**) in culture supernatants of nanowire-modulated aged murine CD8+ T cells. Primary aged CD8+ T cells were incubated on nanowires for 48 h, followed by 48 h of TCR stimulation, and cytokine concentrations were measured in the media. Data are presented as mean ± s.e.m. Two-way ANOVA with Tukey's multiple comparison test (n = 4 nanowires).

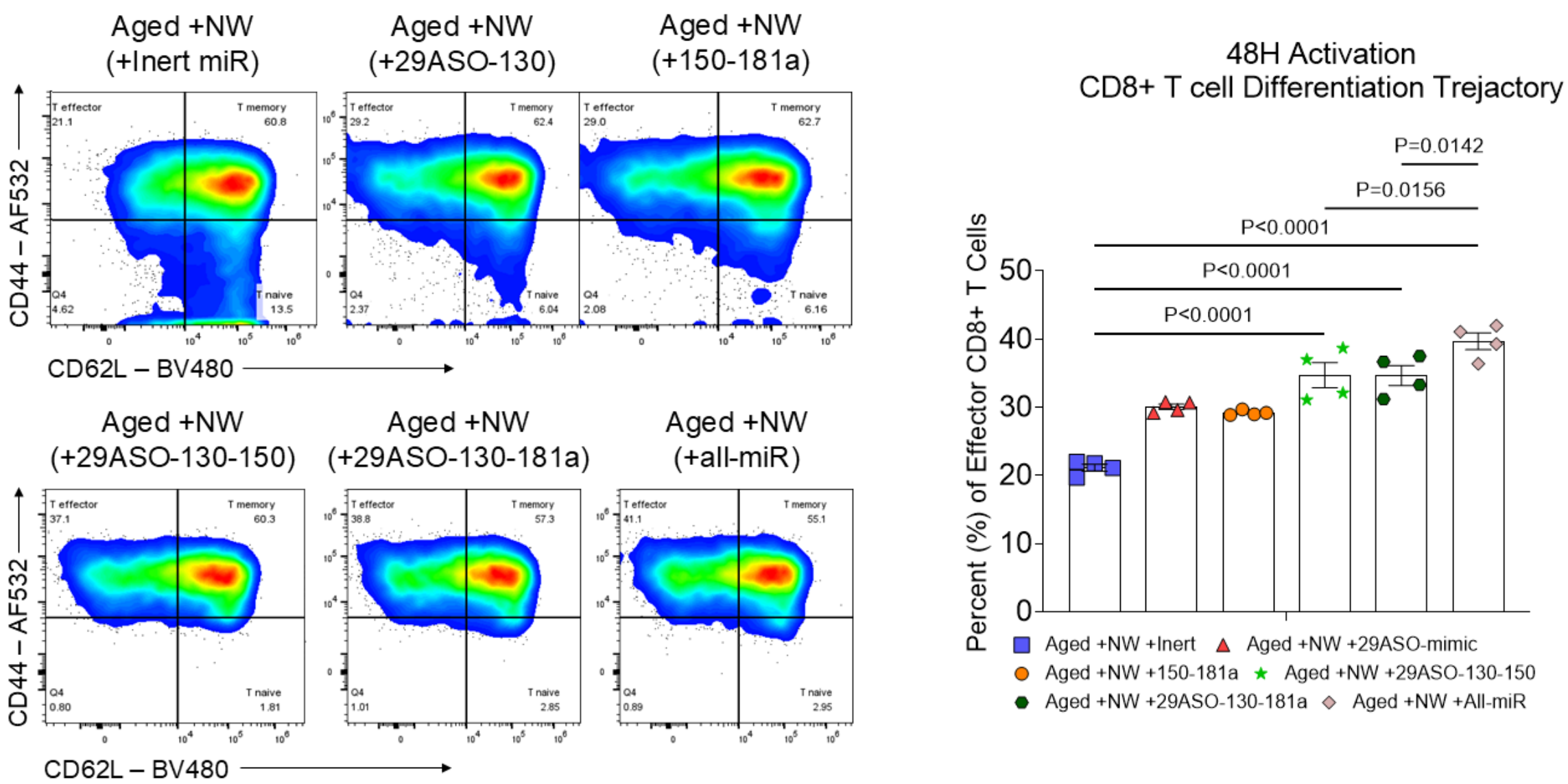

Figure S9. Deconvolution of the multiplexed miRNA cocktail identifies progressive effector-skewing effects in aged murine CD8+ T cells.

Flow cytometry gating of CD44 (AF532) vs CD62L (BV480) on aged murine CD8+ T cells after 48-hour activation to classify CD8+ T cell subsets — T_{Naive} (CD44- CD62L+), T_{Memory} (CD44+ CD62L+), $T_{Effector}$ (CD44+ CD62L-)— across six conditions: Aged +NW (+Inert miR), Aged +NW (+miR-29-ASO +miR-130), Aged +NW (+miR-150 +miR-181a), Aged +NW (+miR-29-ASO +miR-130 +miR-150), Aged +NW (+miR-29-ASO +miR-130 +miR-181a), and Aged +NW (+all-miR). And bar graph quantifying the percentage of $T_{Effector}$ in each reduced or full multiplexed miRNA combinations. Data are presented as mean ± s.e.m. Two-way ANOVA with Tukey's multiple comparison test (n = 4 nanowires).

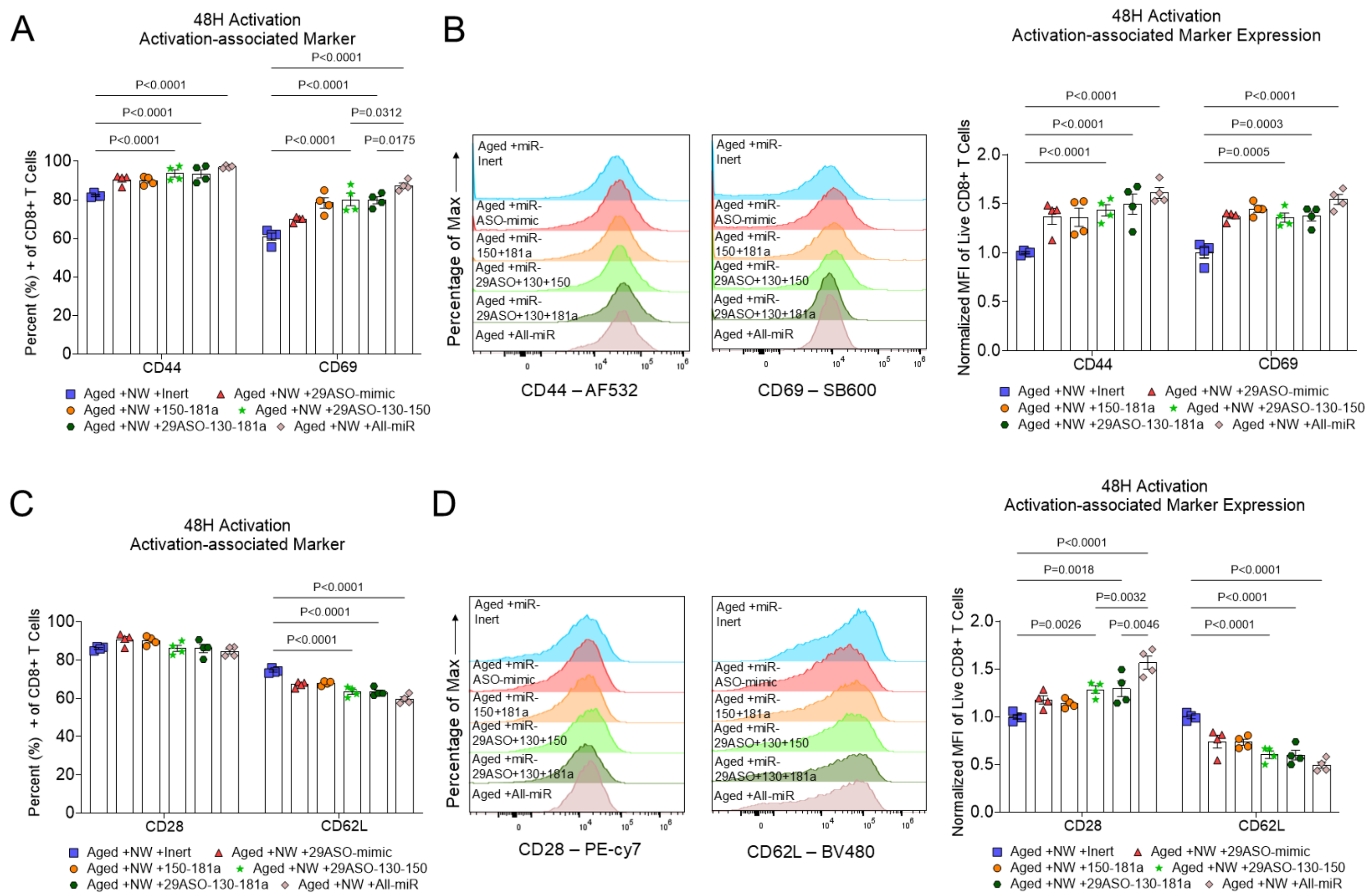

Figure S10. Reduced and full multiplexed miRNA combinations differentially enhance activation-marker frequency and expression in aged murine CD8+ T cells.

A, Frequency of activation-associated markers CD44+, and CD69+ in nanowire-modulated aged murine CD8+ T cell groups treated with reduced or full multiplexed miRNA combinations. Data are presented as mean ± s.e.m. Two-way ANOVA with Tukey's multiple comparison test (n = 4 nanowires). **B**, Flow cytometry histograms (Left) and bar graph (Right) of the normalized expression (MFI) of activation-associated markers: CD44, and CD69 in nanowire-modulated aged murine CD8+ T cell groups treated with reduced or full multiplexed miRNA combinations. Data are presented as mean ± s.e.m. Two-way ANOVA with Tukey's multiple comparison test (n = 4 nanowires). **C**, Frequency of activation-associated markers CD28+, and CD62L- in nanowire-modulated aged murine CD8+ T cell groups treated with reduced or full multiplexed miRNA combinations. Data are presented as mean ± s.e.m. Two-way ANOVA with Tukey's multiple comparison test (n = 4 nanowires). **D**, Flow cytometry histograms (Left) and bar graph (Right) of the normalized expression (MFI) of activation-associated markers: CD28, and CD62L in nanowire-modulated aged murine CD8+ T cell groups treated with reduced or full multiplexed miRNA combinations. Data are presented as mean ± s.e.m. Two-way ANOVA with Tukey's multiple comparison test (n = 4 nanowires).

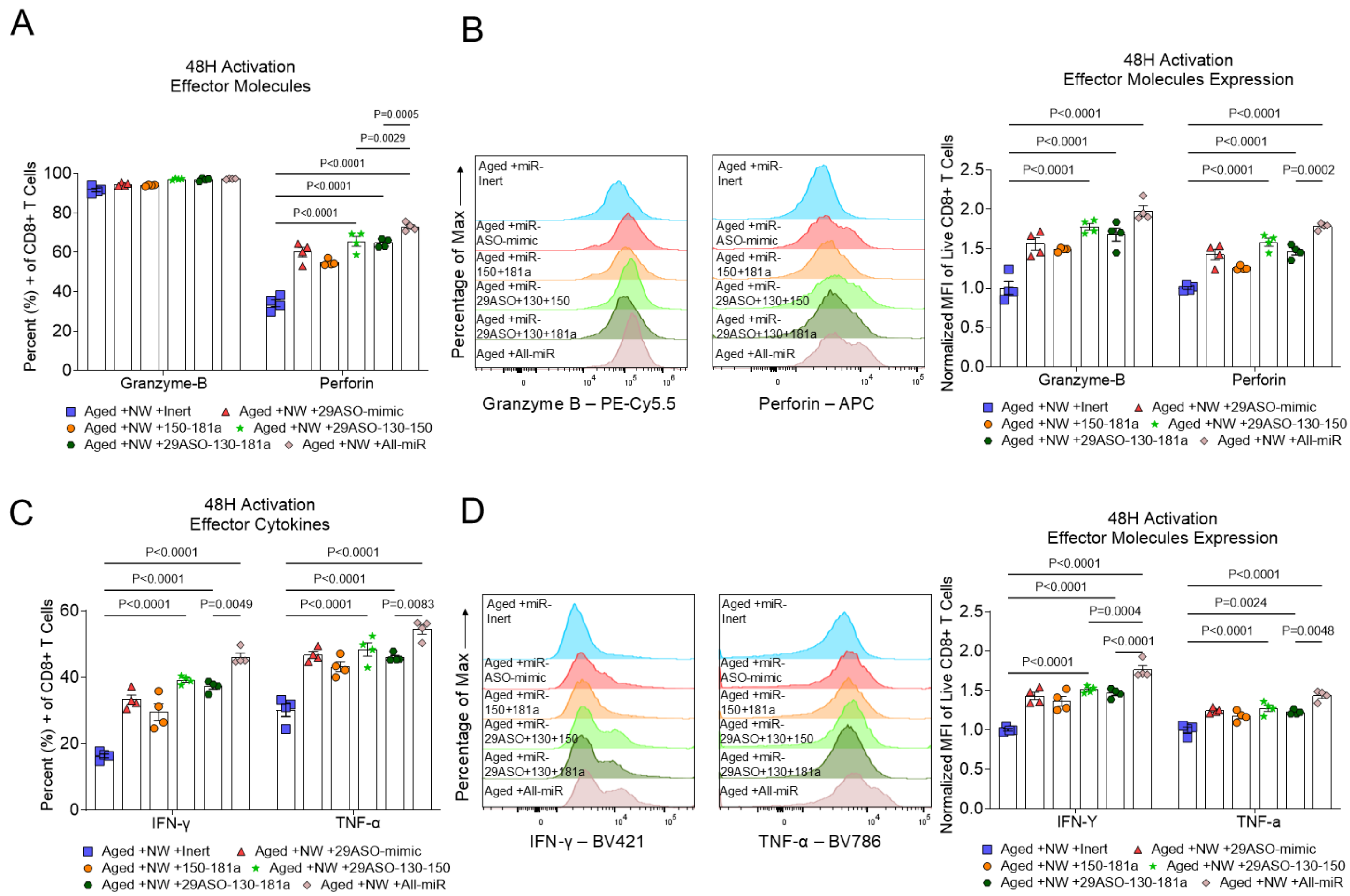

Figure S11. Reduced and full multiplexed miRNA combinations differentially increase cytotoxic molecule and effector cytokine production in aged murine CD8+ T cells.

A, Frequency of effector molecules (Granzyme B, Perforin) secreting cells in nanowire-modulated aged murine CD8+ T cell groups treated with reduced or full multiplexed miRNA combinations. Data are presented as mean ± s.e.m. Two-way ANOVA with Tukey's multiple comparison test (n = 4 nanowires). **B**, Flow cytometry histograms (Left) and bar graph (Right) of the normalized secretion (MFI) of effector molecules (Granzyme B, Perforin) in nanowire-modulated aged murine CD8+ T cell groups treated with reduced or full multiplexed miRNA combinations. Data are presented as mean ± s.e.m. Two-way ANOVA with Tukey's multiple comparison test (n = 4 nanowires). **C**, Frequency of effector cytokines (IFN-γ, TNF-α) secreting cells in nanowire-modulated aged murine CD8+ T cell groups treated with reduced or full multiplexed miRNA combinations. Data are presented as mean ± s.e.m. Two-way ANOVA with Tukey's multiple comparison test (n = 4 nanowires). **D**, Flow cytometry histograms (Left) and bar graph (Right) of the normalized secretion (MFI) of effector cytokines (IFN-γ, TNF-α) in nanowire-modulated aged murine CD8+ T cell groups treated with reduced or full multiplexed miRNA combinations. Data are presented as mean ± s.e.m. Two-way ANOVA with Tukey's multiple comparison test (n = 4 nanowires).

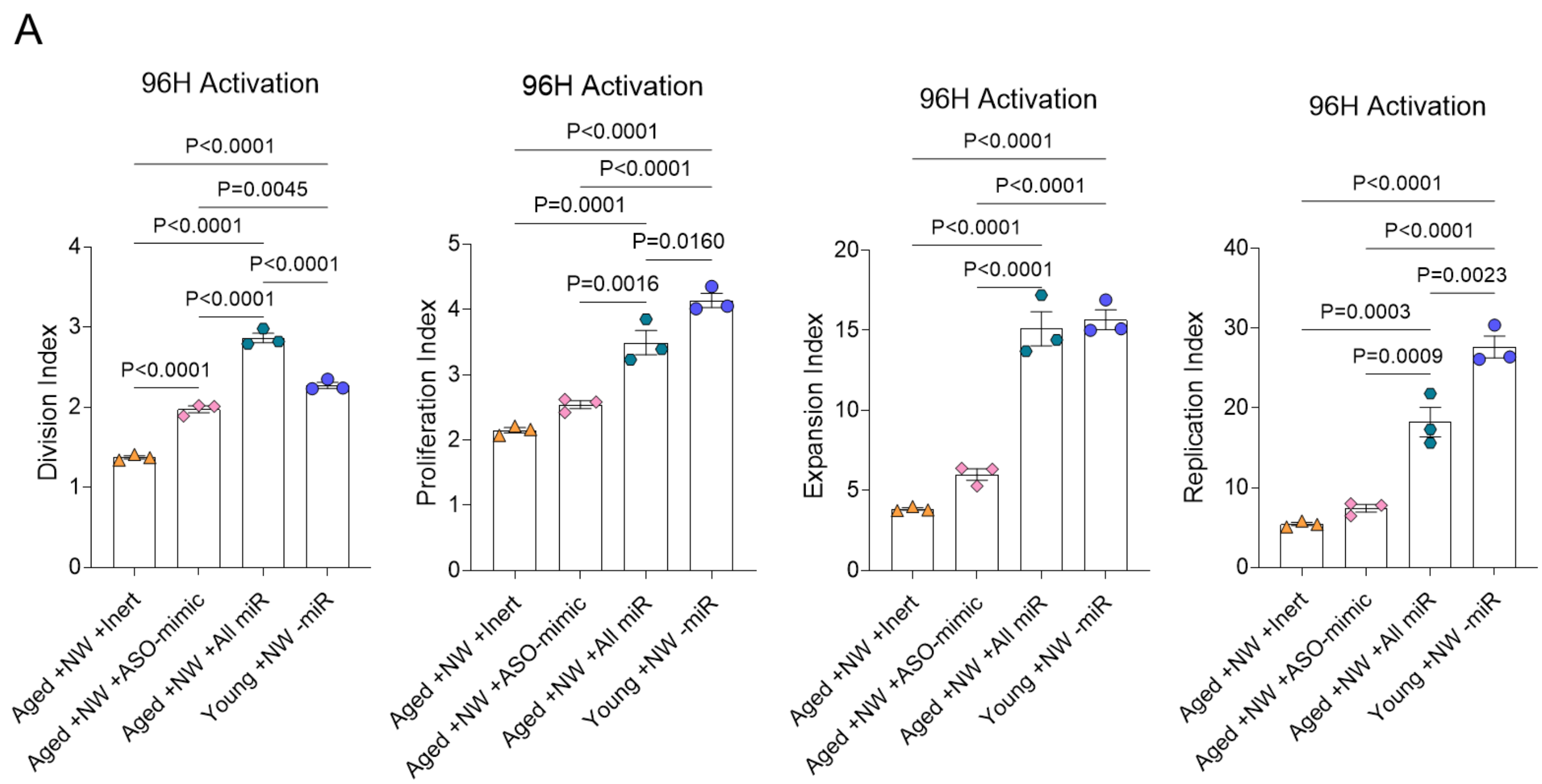

Figure S12. Multiplexed miRNA delivery improves CFSE-based proliferation metrics in aged murine CD8+ T cells after activation.

A, Quantitative analysis of CFSE-based proliferation metrics of nanowire-modulated aged murine CD8+ T cells at day 4 post activation. Division Index: average number of divisions undergone by all cells, including undivided. Proliferation Index: average number of divisions among only dividing cells. Expansion Index: fold increase in total cell number. Replication Index: average number of cell divisions, accounting for both dividing and non-dividing populations. Data are presented as mean ± s.e.m. One-way ANOVA with Tukey's multiple comparison test (n = 3 nanowires).

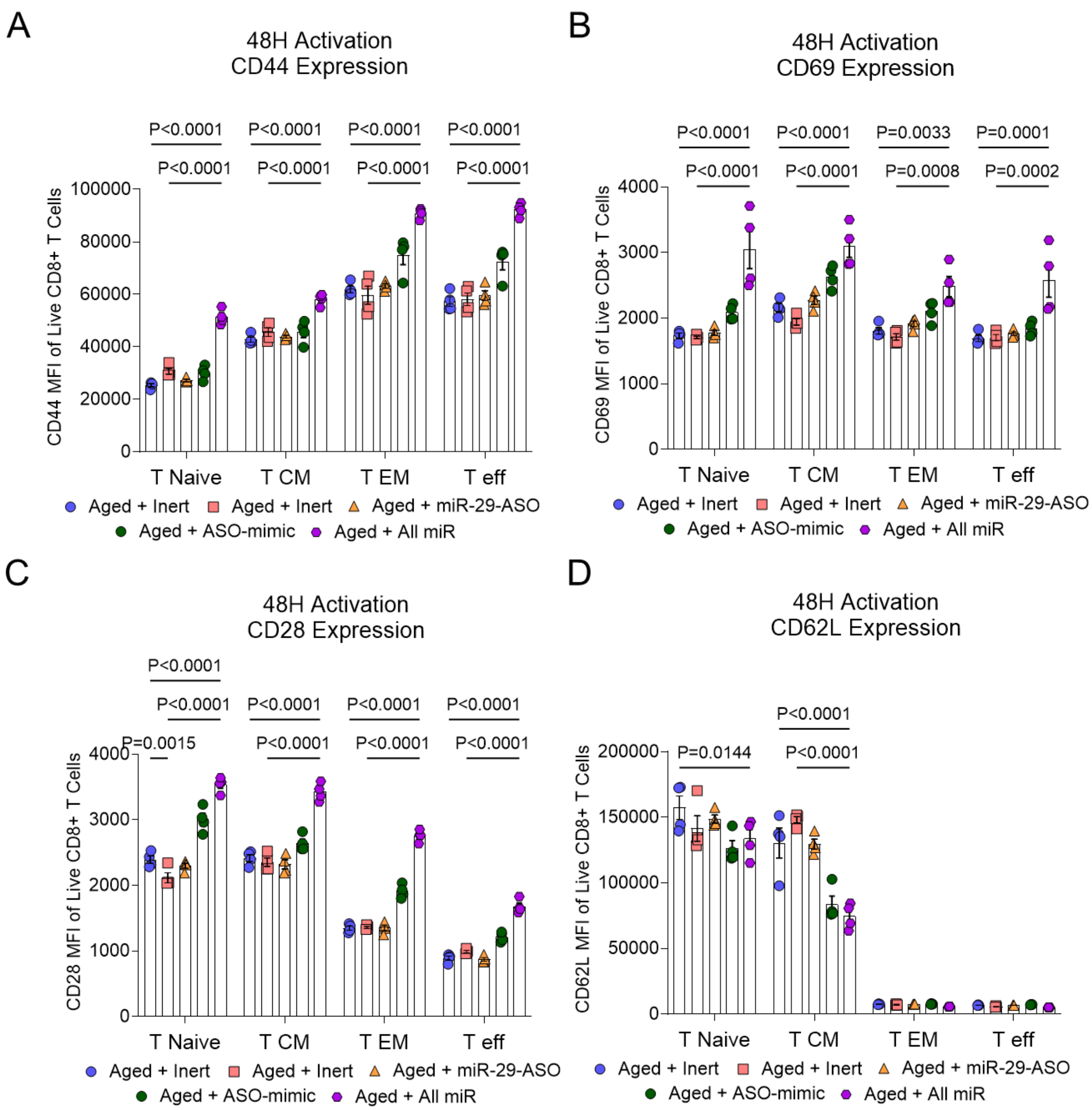

Figure S13. Nanowire-mediated miRNA delivery modulates activation-marker expression across aged human CD8+ T-cell differentiation subsets.

A-D, Bar graph quantification of median fluorescence intensity (MFI) of activation associated marker CD44 (**A**), CD69 (**B**), CD28 (**C**), and CD62L (**D**) after 48-hour activation within the four CD8+ T cell subsets (T_{Naive} (CD45RA+ CD62L+), $T_{Central\ Memory}$ (CD45RO+ CD62L+), $T_{Effector\ Memory}$ (CD45RO+ CD62L-), and $T_{Effector}$ (CD45RA+ CD62L-)) in aged human CD8+ T cells across five miRNA treatment conditions: Aged +NW (+Inert miR), Aged +NW (+miR-29), Aged +NW (+miR-29-ASO), Aged +NW (+ASO-mimic), and Aged +NW (+All-miR). Data are presented as mean ± s.e.m. Two-way ANOVA with Tukey's multiple comparison test (n = 4 nanowires).

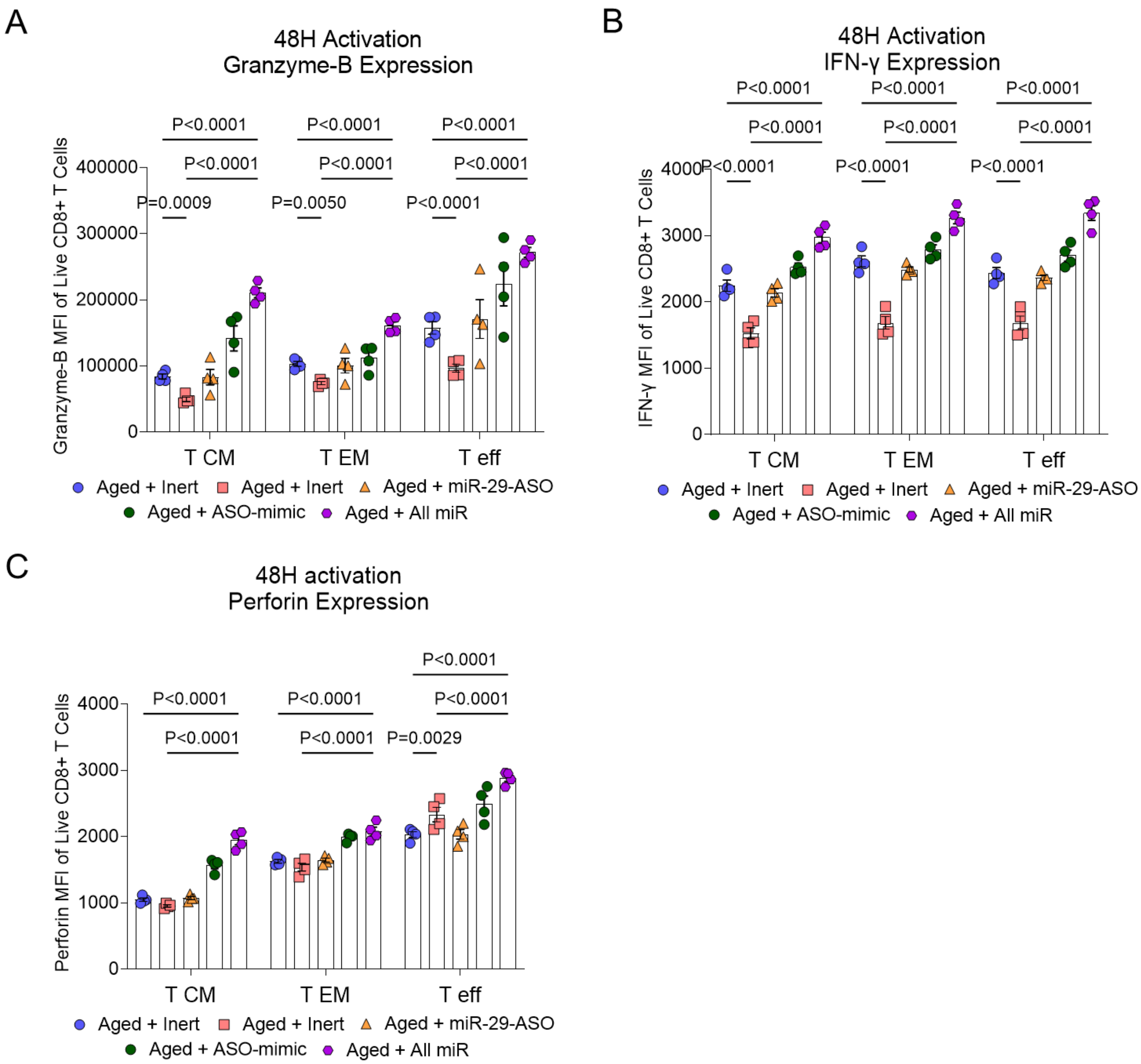

Figure S14. Nanowire-mediated miRNA delivery enhances effector-molecule expression across aged human CD8+ T-cell differentiation subsets.

A-C, Bar graph quantification of median fluorescence intensity (MFI) for the expression of effector molecules Granzyme B (**A**), IFN-γ (**B**), and Perforin (**C**) after 48-hour activation within the three CD8+ T cell subsets ($T_{\text{Central Memory}}$, $T_{\text{Effector Memory}}$, and T_{Effector}) in aged human CD8+ T cells across five miRNA treatment conditions. Data are presented as mean ± s.e.m. One-way ANOVA with Tukey's multiple comparison test (n = 4 nanowires).

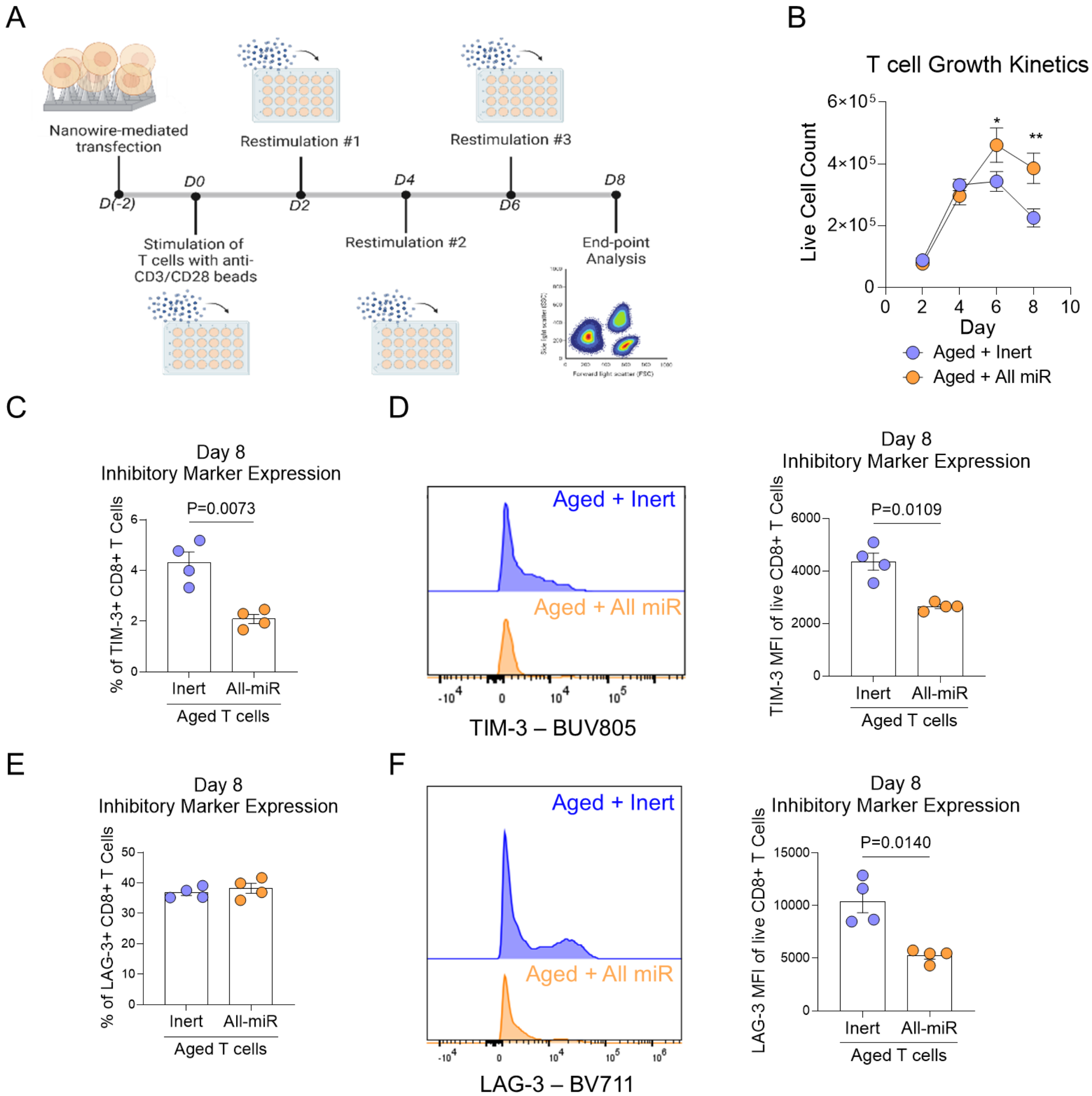

Figure S15. Multiplexed miRNA delivery sustains aged human CD8+ T-cell expansion without increasing selected exhaustion markers after repeated stimulation.

A, Schematic and timeline of human CD8+ T cell exhaustion study through multiple TCR restimulation. Primary aged human CD8+ T cells were incubated on nanowires for 48 h and TCR stimulation was done every 48 h for a total of 4 times before the cells are harvested for end-point analysis. **B**, Growth kinetics analysis of nanowire-modulated aged human CD8+ T cells treated with Inert-miR or All-miR at different timepoint. Data are presented as mean ± s.e.m. Two-tailed, unpaired t-test with Welch's correction was performed at each timepoint (n = 4 nanowires). **C**, Quantification of frequency of exhaustion markers Tim-3+ cells of nanowire-modulated aged human CD8+ T cells treated with Inert-miR or All-miR on Day 8. Data are presented as mean ± s.e.m. Two-tailed, unpaired t-test with Welch's correction was performed (n = 4 nanowires). **D**, Flow cytometry histogram (Left) and bar graph quantification (Right) of the expression (MFI) Tim-3 of nanowire-modulated aged human CD8+ T cells treated with Inert-miR or All-miR on Day 8. Data are presented as mean ± s.e.m. Two-tailed, unpaired t-test with Welch's correction was performed (n = 4 nanowires). **E**, Quantification of frequency of exhaustion markers LAG-3+ cells of nanowire-modulated aged human CD8+ T cells treated with Inert-miR or All-miR on Day 8. Data are presented as mean ± s.e.m. Two-tailed, unpaired t-test with Welch's correction was performed (n = 4 nanowires). **F**, Flow cytometry histogram (Left) and bar graph quantification (Right) of the expression (MFI) LAG-3 of nanowire-modulated aged human CD8+ T cells treated with Inert-miR or All-miR on Day 8. Data are presented as mean ± s.e.m. Two-tailed, unpaired t-test with Welch's correction was performed (n = 4 nanowires).

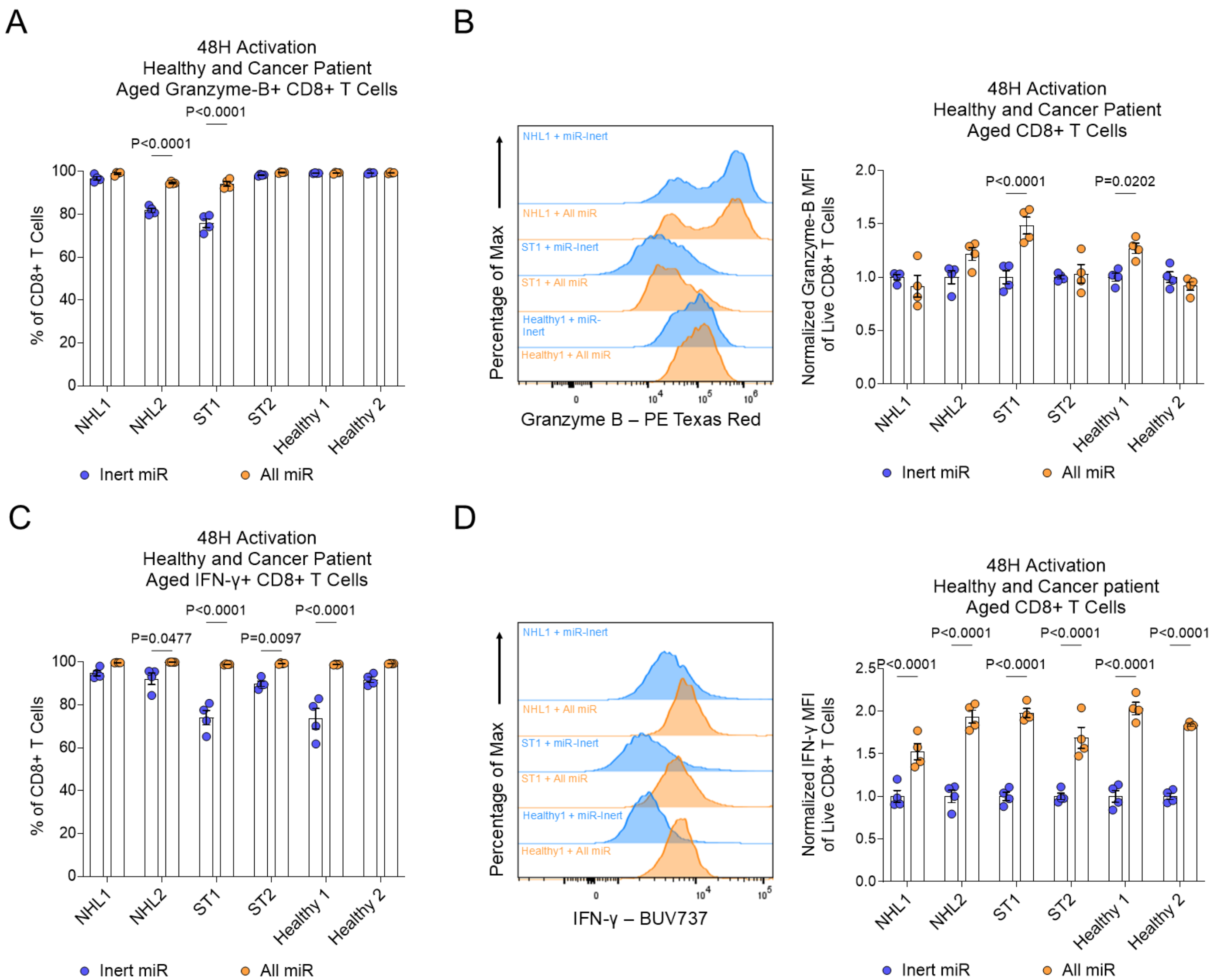

Figure S16. Multiplexed miRNA delivery enhances granzyme B and IFN-γ responses in aged human CD8+ T cells from healthy donors and cancer patients.

A, Quantification on frequency of effector molecules Granzyme-B+ secreting cells in nanowire-modulated human CD8+ T cells from healthy individuals, and donors with current or prior cancer treated with inert miRNA or multiplexed miRNAs. Primary aged human CD8+ T cells are from non-Hodgkin lymphoma survivors (NHL), solid tumor cancer patients who had not received systemic cancer-directed therapy (ST), and healthy aged patients (Healthy). Cells were incubated on nanowires for 48 h and TCR stimulation was done for the next 48 h. Data is presented as mean ± s.e.m. Two-way ANOVA with Tukey's multiple comparison test (n = 4 nanowires). **B**, Flow cytometry histograms (Left) and bar graph (Right) quantification of the normalized expression (MFI) of effector molecules Granzyme in nanowire-modulated human CD8+ T cells from healthy individuals, and donors with current or prior cancer treated with inert miRNA or multiplexed miRNAs. Data are presented as mean ± s.e.m. Two-way ANOVA with Tukey's multiple comparison test (n = 4 nanowires). **C**, Quantification on frequency of effector molecules IFN-γ + secreting cells in nanowire-modulated human CD8+ T cells from healthy individuals, and donors with current or prior cancer treated with inert miRNA or multiplexed miRNAs. Data are presented as mean ± s.e.m. Two-way ANOVA with Tukey's multiple comparison test (n = 4 nanowires). **D**, Flow cytometry histograms (Left) and bar graph (Right) quantification of the normalized expression (MFI) of effector molecules IFN-γ in nanowire-modulated human CD8+ T cells from healthy individuals, and donors with current or prior cancer treated with inert miRNA or multiplexed miRNAs. Data are presented as mean ± s.e.m. Two-way ANOVA with Tukey's multiple comparison test (n = 4 nanowires).

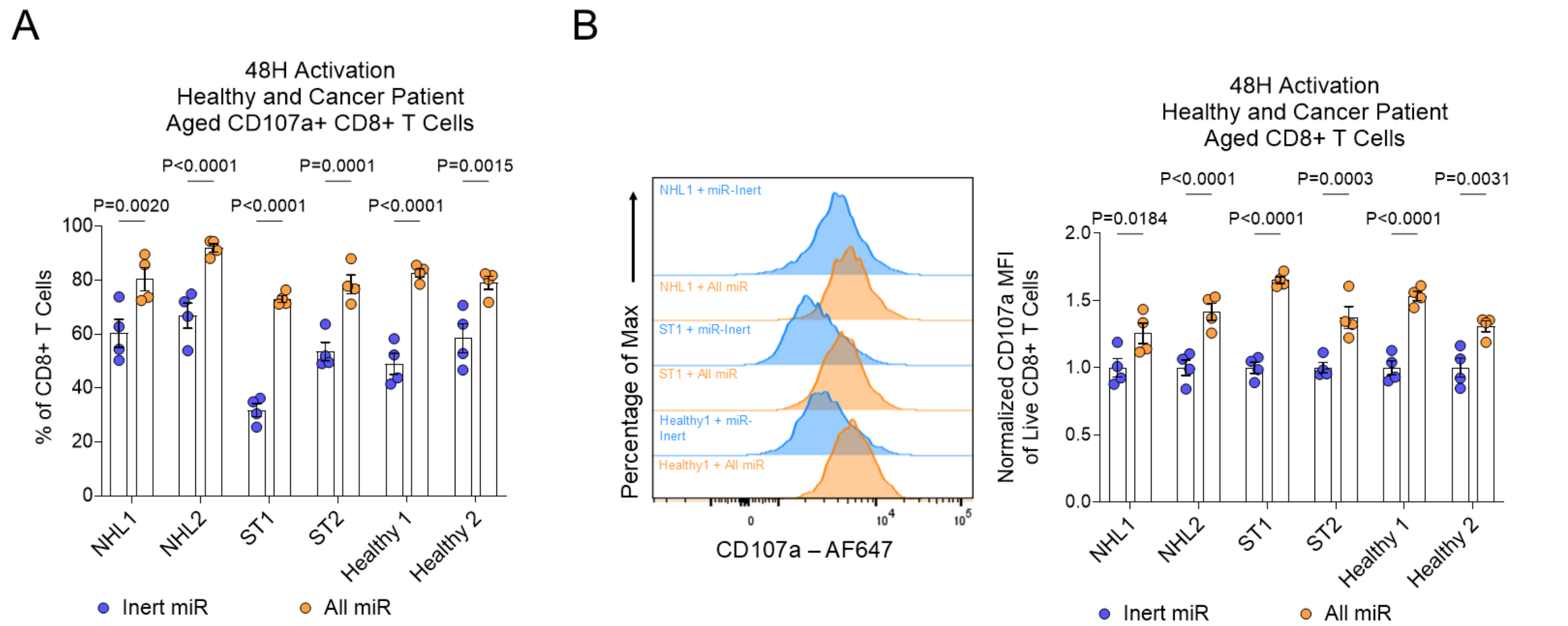

Figure S17. Multiplexed miRNA delivery enhances CD107a degranulation responses in aged human CD8+ T cells from healthy donors and cancer patients.

A, Quantification on frequency of degranulation marker CD107a+ cells in nanowire-modulated human CD8+ T cells from healthy individuals, and donors with current or prior cancer treated with inert miRNA or multiplexed miRNAs. Data are presented as mean ± s.e.m. Two-way ANOVA with Tukey's multiple comparison test (n = 4 nanowires). **B**, Flow cytometry histograms (Left) and bar graph (Right) quantification of the normalized expression (MFI) of degranulation marker CD107a in nanowire-modulated human CD8+ T cells from healthy individuals, and donors with current or prior cancer treated with inert miRNA or multiplexed miRNAs. Primary aged human CD8+ T cells are from non-Hodgkin lymphoma survivors (NHL), solid tumor cancer patients who had not received systemic cancer-directed therapy (ST), and healthy aged patients (Healthy). Cells were incubated on nanowires for 48 h and TCR stimulation was done for the next 48 h. Data is presented as mean ± s.e.m. Two-way ANOVA with Tukey's multiple comparison test (n = 4 nanowires).
